# mTORC1 activity negatively regulates human hair follicle growth and pigmentation

**DOI:** 10.15252/embr.202256574

**Published:** 2023-05-22

**Authors:** Takahiro Suzuki, Jérémy Chéret, Fernanda Dinelli Scala, Aysun Akhundlu, Jennifer Gherardini, Dana‐Lee Demetrius, James D B O'Sullivan, Gorana Kuka Epstein, Alan J Bauman, Constantinos Demetriades, Ralf Paus

**Affiliations:** ^1^ Dr Phillip Frost Department of Dermatology & Cutaneous Surgery University of Miami Miller School of Medicine Miami FL USA; ^2^ Foundation for Hair Restoration Miami FL USA; ^3^ Bauman Medical Group Boca Raton FL USA; ^4^ Max Planck Institute for Biology of Ageing Cologne Germany; ^5^ Cologne Excellence Cluster on Cellular Stress Responses in Aging‐Associated Diseases (CECAD) University of Cologne Cologne Germany; ^6^ Monasterium Laboratory Muenster Germany; ^7^ CUTANEON Hamburg Germany

**Keywords:** alpha‐MSH/MC1R, melanocyte, mTORC1, rapamycin, tuberous sclerosis, Molecular Biology of Disease, Signal Transduction, Skin

## Abstract

Dysregulation of the activity of the mechanistic target of rapamycin complex 1 (mTORC1) is commonly linked to aging, cancer, and genetic disorders such as tuberous sclerosis (TS), a rare neurodevelopmental multisystemic disease characterized by benign tumors, seizures, and intellectual disability. Although patches of white hair on the scalp (poliosis) are considered as early signs of TS, the underlying molecular mechanisms and potential involvement of mTORC1 in hair depigmentation remain unclear. Here, we have used healthy, organ‐cultured human scalp hair follicles (HFs) to interrogate the role of mTORC1 in a prototypic human (mini‐)organ. Gray/white HFs exhibit high mTORC1 activity, while mTORC1 inhibition by rapamycin stimulated HF growth and pigmentation, even in gray/white HFs that still contained some surviving melanocytes. Mechanistically, this occurred via increased intrafollicular production of the melanotropic hormone, α‐MSH. In contrast, knockdown of intrafollicular TSC2, a negative regulator of mTORC1, significantly reduced HF pigmentation. Our findings introduce mTORC1 activity as an important negative regulator of human HF growth and pigmentation and suggest that pharmacological mTORC1 inhibition could become a novel strategy in the management of hair loss and depigmentation disorders.

## Graphical abstract

Gray/white hair follicles exhibit high mTORC1 activity. mTORC1 inhibition by rapamycin stimulates hair follicle growth and pigmentation via increased intrafollicular production of the melanotropic hormone, α‐MSH.

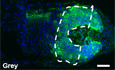

## Introduction

The mechanistic target of rapamycin complex 1 (mTORC1) is a signaling hub that senses the cellular environment to coordinately regulate multiple fundamental physiological processes, including cell growth and proliferation/apoptosis (Ben‐Sahra & Manning, 2017; Kim & Guan, 2019; Cho *et al*, 2021), autophagy (Noda, 2017; Fukuda & Shiozaki, 2021; Mohanasundaram *et al*, 2022), and Wnt signaling (Zeng *et al*, 2018; Evans *et al*, 2021). mTORC1 activity also impinges on cell death regulation by coordinating defined metabolic pathways (Zhu *et al*, 2022). Instead, dysregulated mTORC1 activity can promote aging (Fernandes & Demetriades, 2021; Lei *et al*, 2022; Simcox & Lamming, 2022) and is associated with cancer (Mrozek *et al*, 2021; McClellan *et al*, 2022; Morales *et al*, 2022), autism spectrum disorders (Chaudry & Vasudevan, 2022; Teles e Silva *et al*, 2022), and the neurocutaneous disorder, tuberous sclerosis (hereafter referred to as TS to distinguish it from the TS protein complex, TSC).

TS is caused by loss‐of‐function mutations in TSC1 or TSC2, which comprise the TSC complex, the main endogenous mTORC1 negative regulator (Demetriades *et al*, 2014; Wataya‐Kaneda, 2015; Henske *et al*, 2016; Rehbein *et al*, 2021; Curatolo *et al*, 2022; Ferreri *et al*, 2022). TS is typically associated with hypopigmentation of skin (hypomelanotic macules; also referred to as “ash leaf spots”) and hair (poliosis), which is often used as an early diagnostic sign of TS (Cartron *et al*, 2021; Islam, 2021; Takahashi *et al*, 2022). Other manifestations of TS are the formation of various tumors and severe neurodevelopmental and renal abnormalities (Carmignac *et al*, 2021; Girodengo *et al*, 2022; Luo *et al*, 2022). Given the poliosis phenotype associated with TS, we hypothesized that mTORC1 activity may regulate human hair follicle (HF) pigmentation and hair graying (canities)—a complex, temporarily reversible, and stressor‐sensitive process that ultimately shuts off melanin production (melanogenesis) in the HF pigmentary unit (HFPU), typically long before it becomes irreversible due to a depletion of HF melanocyte stem cells (O'Sullivan *et al*, 2021; Rosenberg *et al*, 2021).

While topical application of rapamycin, the prototypic mTORC1 inhibitor (Chen & Zhou, 2020), may partially reverse epidermal hypopigmentation in macules of selected TS patients (Wataya‐Kaneda *et al*, 2012; Wataya‐Kaneda, 2015), it is unknown whether and how rapamycin impacts on HF pigmentation. This is important, since epidermal and HF melanocytes represent very different cell populations, underlie partially distinct molecular controls, and display many differential features (Slominski *et al*, 2005; Gáspár *et al*, 2011; Paus, 2011; Tobin, 2011; O'Sullivan *et al*, 2021). Also, TSC loss‐of‐function in mouse tissues and cultured fibroblasts shows upregulated BMAL‐1 and/or PER‐1 expression (Lipton *et al*, 2017; Ramanathan *et al*, 2018). However, we had previously shown that downregulating the expression of these core elements of the peripheral clock in human HFs *ex vivo* actually *promotes* HF pigmentation (Hardman *et al*, 2015). Thus, the role of mTORC1 signaling in the control of human HF pigmentation and HF melanocytes remains unknown.

In the current study, we have probed our working hypothesis by characterizing the impact of mTORC1 activity and of its key negative regulator, TSC2, on human HF pigmentation *ex vivo*. Given that melanocyte activities in the HFPU are entirely controlled by their epithelial and mesenchymal tissue environment (Slominski *et al*, 2005; Paus, 2011; Tobin, 2011; Nicu *et al*, 2021; O'Sullivan *et al*, 2021; Tiede *et al*, 2021), the *physiologically and clinically relevant* controls of melanocyte activity can only be elucidated directly in the human target organ itself (Sevilla *et al*, 2022). For this, we used the organ culture of microdissected, healthy human scalp HFs (Langan *et al*, 2015) as a uniquely instructive model system for both identifying novel controls of human HF pigmentation (Gáspár *et al*, 2011; Samuelov *et al*, 2013; Hardman *et al*, 2015; Chéret *et al*, 2020; Nicu *et al*, 2021; Tiede *et al*, 2021) and for interrogating the functions of mTORC1 and TSC2 in human organ physiology. mTORC1 activity was pharmacologically inhibited by rapamycin or enhanced by selective TSC2 knockdown *ex vivo*, and the impact of these manipulations on key pigmentation read‐out parameters was quantitatively assessed in organ‐cultured pigmented or graying/white human scalp HFs.

These *ex vivo* analyses revealed that mTORC1 inhibition by rapamycin prolongs the duration of the phase of active hair growth (anagen) and stimulates intrafollicular pigmentation, even in selected gray/white human HFs with surviving melanocytes, via increased production of the melanotropic hormone, α‐MSH by the anagen HF epithelium. In contrast, TSC2 silencing significantly reduced HF pigmentation while spontaneously aged gray/white HFs exhibited high mTORC1 activity. Taken together, these analyses confirm our working hypothesis and demonstrate that mTORC1 activity is a previously underappreciated and therapeutically targetable key regulator of human hair growth *and* pigmentation.

## Results and Discussion

### Hair graying is associated with a significant increase in intrafollicular mTORC1 activity

To probe our working hypothesis that mTORC1 activity may negatively regulate human HF pigmentation, we first asked whether mTORC1 activity differs between fully pigmented and graying/white human scalp HFs *in vivo*, since hair graying (canities) constitutes an excellent model system for exploring human tissue aging in an organ context (O'Sullivan *et al*, 2021; Rosenberg *et al*, 2021). To do so, we assessed phosphorylation of the ribosomal protein S6 (p‐S6), which is commonly used as an indicator of mTORC1 activity in immunohistochemistry (Ding *et al*, 2016; Moustafa‐Kamal *et al*, 2020; Cho *et al*, 2021), and the protein levels of its most important negative upstream regulator, TSC2 (Holz & Blenis, 2005; Wataya‐Kaneda, 2015; Rehbein *et al*, 2021). This was done by standardized quantitative immunohistomorphometry (Hardman *et al*, 2015; Chéret *et al*, 2018; Hawkshaw *et al*, 2018; Purba *et al*, 2019) in freshly frozen pigmented and gray/white HFs obtained from four patients undergoing hair transplant surgery (age range 54–78 years old).

**Figure 1:**
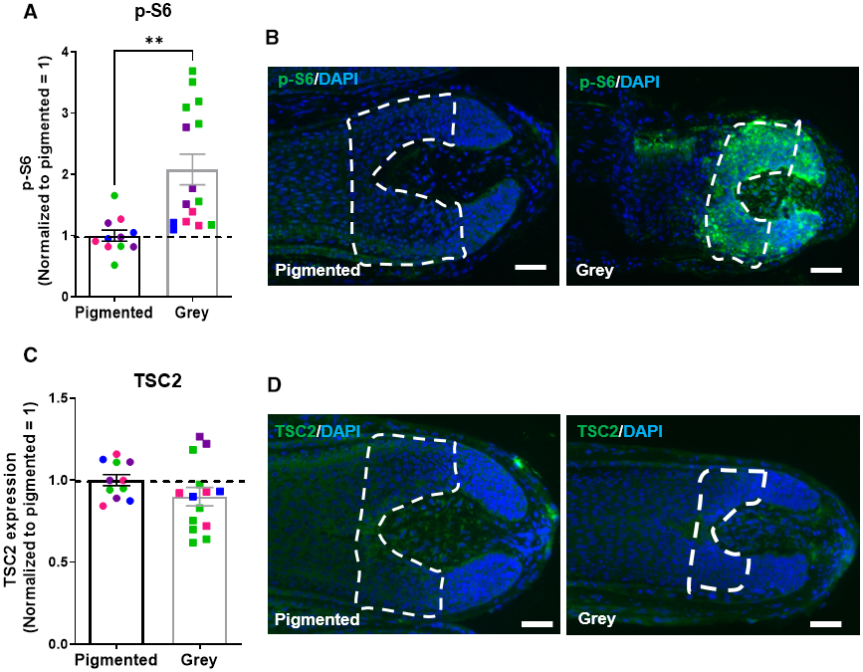
mTORC1 activity is significantly upregulated while TSC2 protein expression is not altered in gray/white human anagen hair follicles *in vivo* Quantitative analysis of phosphorylated S6 (p‐S6; mTORC1 activity read‐out) immunoreactivity. *N* = 11–15 pigmented or gray HFs from four different donors.Representative images of p‐S6 immunofluorescence.Quantitative analysis of TSC2 protein levels. *N* = 11–14 pigmented or gray HFs from four different donors.Representative images of TSC2 immunofluorescence. Data information: Only anagen VI HFs were investigated and analyzed immediately after surgery. Analyses were performed in defined reference areas (dotted areas) in the HFPU. Mean ± SEM, Student's *t*‐test, **P < 0.01. Scale bar: 50 μm. Samples from each donor represented by a different color. Nuclei stained with DAPI. Source data are available online for this figure.

These analyses showed a significant increase in p‐S6 immunoreactivity within the HFPU of human anagen VI scalp HFs affected by canities, while TSC2 protein expression itself was unchanged in gray/white HFs (Fig 1A–D). This provided a first, clinically relevant phenomenological indication that the upregulation of intrafollicular mTORC1 activity (Fig 1A and B), independently from changes in TSC2 protein levels (Fig 1C and D), might somehow be involved in hair graying. Moreover, as cell and tissue aging typically is associated with increased mTORC1 activity levels (Fernandes & Demetriades, 2021; Mota‐Martorell *et al*, 2022), the increased p‐S6 protein expression supports the concept that the human HFPU undergoes premature aging processes during canities (Tobin & Paus, 2001; O'Sullivan *et al*, 2021).

### Rapamycin promotes human HF pigmentation and stimulates hair growth by anagen prolongation *ex vivo*

**Figure 2:**
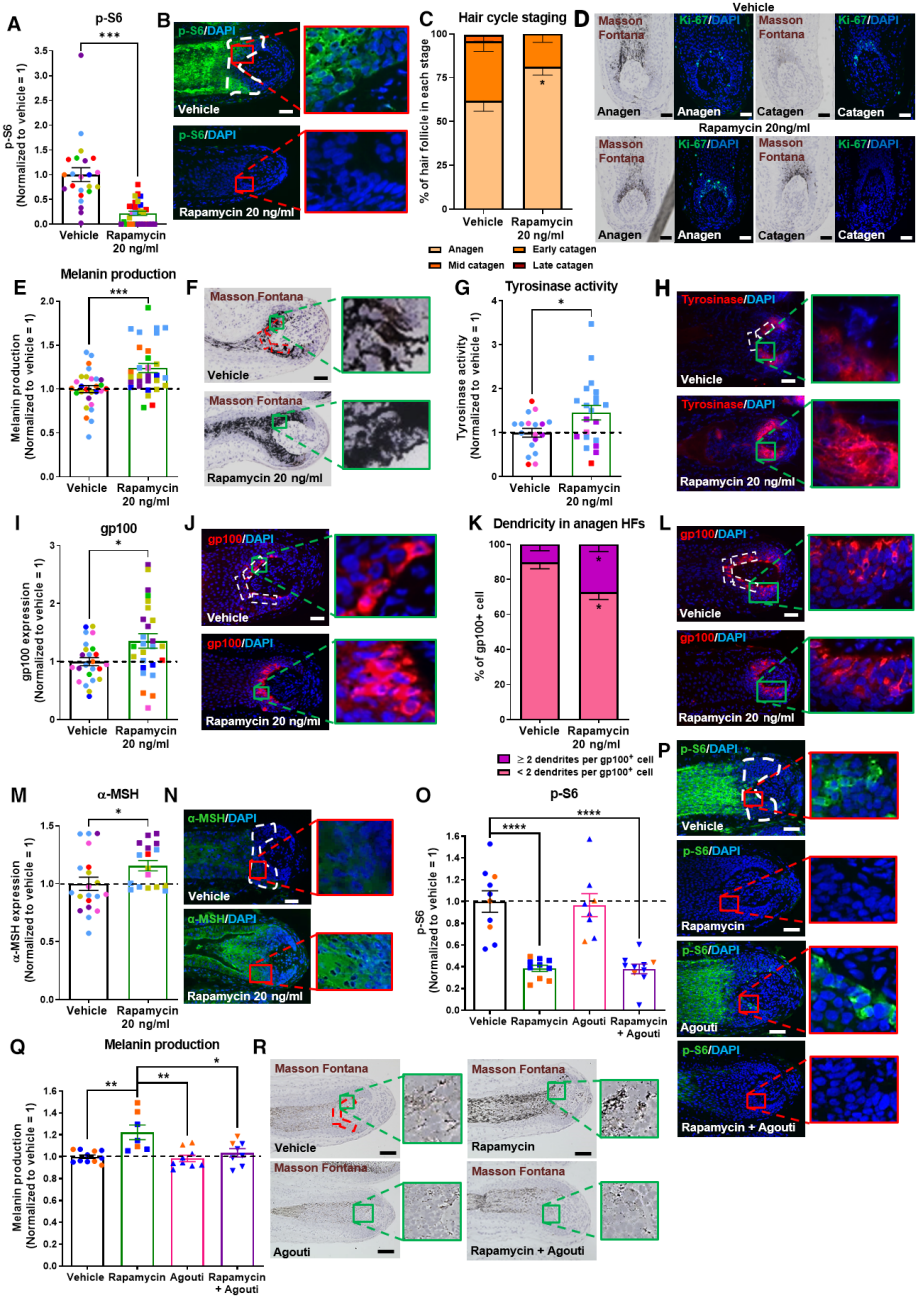
mTORC1 inhibition stimulates human scalp hair follicle growth and pigmentation through MC1R activation Quantitative analysis of phosphorylated S6 (p‐S6; mTORC1 activity read‐out) immunoreactivity. *N* = 23–30 anagen VI HFs from eight different donors treated with Rapamycin 20 ng/ml or untreated (vehicle) for 7 days.Representative images of p‐S6 immunofluorescence.Hair cycle staging was performed using Ki‐67 and Masson–Fontana histochemistry (Kloepper *et al*, 2010). Mean ± SEM; *N* = 36–39 HFs per group from six different donors treated with Rapamycin 20 ng/ml or vehicle (control) for 7 days.Representative fluorescence images of Ki‐67 and bright‐field microscopic images of Masson–Fontana.Quantitative histomorphometry of melanin production by Masson–Fontana histochemistry. *N* = 28–30 anagen VI HFs from eight different donors treated with Rapamycin 20 ng/ml or untreated (vehicle) for 7 days.Representative bright‐field microscopic images of Masson–Fontana histochemistry.Quantitative analysis of tyrosinase activity. *N* = 18–21 anagen VI HFs from five different donors treated with Rapamycin 20 ng/ml or untreated (vehicle) for 7 days.Representative images of tyrosinase activity immunofluorescence.Quantitative analysis of gp100 expression. *N* = 24–26 anagen VI HFs from eight different donors treated with Rapamycin 20 ng/ml or untreated (vehicle) for 7 days.Representative images of gp100 immunofluorescence.Quantitative analysis of melanocyte dendricity. *N* = 22–24 anagen VI HFs from six different donors treated with Rapamycin 20 ng/ml or untreated (vehicle) for 7 days.Representative images of gp100 immunofluorescence.Quantitative analysis of α‐MSH expression. *N* = 17–20 anagen VI HFs from five different donors treated with Rapamycin 20 ng/ml or untreated (vehicle) for 7 days.Representative images of α‐MSH immunofluorescence.Quantitative analysis of phosphorylated S6 (p‐S6; mTORC1 activity read‐out) immunoreactivity. *N* = 8–10 anagen VI HFs from two different donors treated with Rapamycin 20 ng/ml, 2 μg/ml Agouti, Rapamycin + Agouti or untreated (vehicle) for 7 days.Representative images of p‐S6 immunofluorescence.Quantitative histomorphometry of melanin production by Masson–Fontana histochemistry. *N* = 8–11 anagen VI HFs from two different donors treated with Rapamycin 20 ng/ml, 2 μg/ml Agouti, Rapamycin + Agouti or untreated (vehicle) for 7 days.Representative bright‐field microscopic images of Masson–Fontana histochemistry. Data information: Only anagen VI HFs (except for C and D where all HFs were analyzed) were investigated and analyses performed in defined reference areas (dotted areas) in the HFPU. Mean ± SEM, unpaired Student's *t*‐test (A, C, E, G, I, K, M), *P < 0.05, **P < 0.01, ***P < 0.001, ****P < 0.0001 and one‐way ANOVA (O, Q). Scale bar: 50 μm. Samples from each donor represented by a different color. Nuclei stained with DAPI. Source data are available online for this figure.

Next, we asked whether pharmacological inhibition of intrafollicular mTORC1 activity using the specific mTORC1 inhibitor, rapamycin (Chen & Zhou, 2020; Karalis & Bateup, 2021; Wang & Eisen, 2022), further stimulates pigment production in healthy, fully pigmented human scalp anagen VI HFs in organ culture (Gáspár *et al*, 2011; Hardman *et al*, 2015; Langan *et al*, 2015). As expected, but never previously documented, rapamycin significantly inhibited mTORC1 activity in the hair matrix of human scalp HFs in anagen VI, which harbors the HFPU (Tobin, 2011), as shown by significantly decreased intrafollicular S6 phosphorylation after 7 days of culture (Fig 2A and B). This confirmed that our *ex vivo* assay system is well‐suited for interrogating and experimentally manipulating mTORC1 activity directly in a human model (mini‐)organ, under physiologically and clinically relevant conditions.

**Figure EV1:**
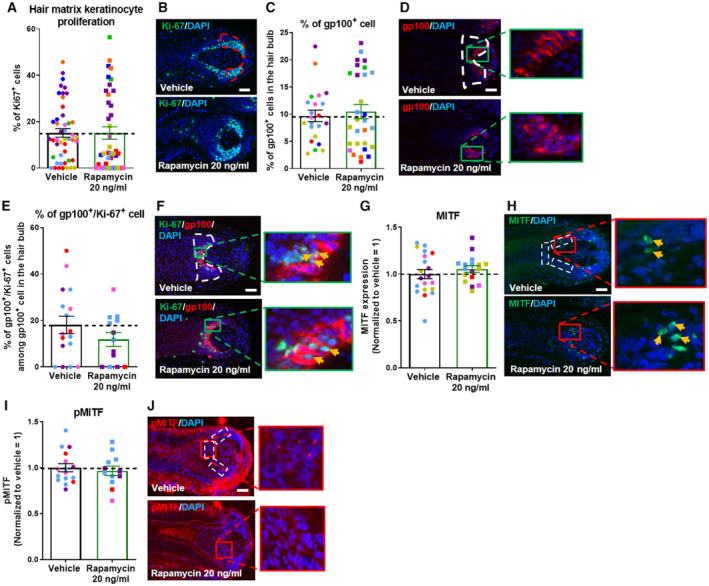
mTORC1 inhibition did not affect MITF expression or the number and proliferation state of melanocytes in human scalp hair follicles Quantitative analysis of Ki‐67^+^ cell number. *N* = 40–48 anagen VI HFs from eight different donors treated with Rapamycin 20 ng/ml or untreated (vehicle) for 7 days.Representative images of Ki‐67 immunofluorescence.Quantitative immunohistomorphometry of the number of gp100^+^ cells. *N* = 22–29 anagen VI HFs from eight different donors treated with Rapamycin 20 ng/ml or untreated (vehicle) for 7 days.Representative images of gp100 immunofluorescence.Quantitative analysis of gp100^+^/Ki‐67^+^ cell number. *N* = 13–17 anagen VI HFs from four different donors treated with Rapamycin 20 ng/ml or untreated (vehicle) for 7 days.Representative images of gp100/Ki‐67 immunofluorescence. Yellow arrows indicate gp100^+^/Ki‐67^+^ cells.Quantitative analysis of MITF expression. *N* = 17–20 anagen VI HFs from five different donors treated with Rapamycin 20 ng/ml or untreated (vehicle) for 7 days.Representative images of MITF immunofluorescence. Yellow arrows indicate MITF^+^ cells.Quantitative analysis of MITF phosphorylation (pMITF). *N* = 12–16 anagen VI HFs from four different donors treated with Rapamycin 20 ng/ml or untreated (vehicle) for 7 days.Representative images of pMITF immunofluorescence. Data information: Only anagen VI HFs (except for A and B where all HFs were analyzed) were investigated and analyses performed in defined reference areas (dotted areas) in the HFPU. Mean ± SEM, Student's *t*‐test (A, C, G, I) or Mann–Whitney U‐test (E). Scale bar: 50 μm. Samples from each donor represented by a different color. Nuclei stained with DAPI. Source data are available online for this figure.

Since HF pigmentation is strictly coupled to the cyclic growth and regression activity of the HF and occurs only during active hair growth (anagen) (Paus & Cotsarelis, 1999; Slominski *et al*, 2005; Tobin, 2011; O'Sullivan *et al*, 2021), we also assessed whether mTORC1 inhibition by rapamycin alters HF cycling *ex vivo* and thus may affect HF pigmentation also via prolonging the duration of anagen, which is the only hair cycle phase during which the HFPU synthesizes melanin (Slominski *et al*, 2005; Tobin, 2011; Hardman *et al*, 2015; O'Sullivan *et al*, 2021). Quantitative hair cycle histomorphometry (Kloepper *et al*, 2010) revealed that rapamycin indeed significantly prolongs the duration of anagen along with anagen‐associated intrafollicular melanogenesis (Fig 2C and D), yet without affecting hair matrix keratinocyte proliferation significantly (as measured by quantitative Ki‐67 (immuno‐)histomorphometry; Fig EV1A and B).

These data indicate that rapamycin promotes human hair growth by prolonging anagen, which is, the hair cycle phase during which the HF actively generates a pigmented hair shaft (Slominski *et al*, 2005; Tobin, 2011). Moreover, this also suggests that mTORC1 inhibition can expand the therapeutic window of opportunity during which repigmentation of graying/white HFs may be induced via reactivation of the HFPU, since this is exclusively possible during anagen III‐VI (Slominski *et al*, 2005; O'Sullivan *et al*, 2021; Rosenberg *et al*, 2021). That mTORC1 inhibition prolongs anagen is also clinically relevant for the management of the vast majority of hair loss disorders, since these show premature anagen termination/catagen induction (Paus & Cotsarelis, 1999; Paus & Foitzik, 2004; Qi & Garza, 2014). Thus, if anagen prolongation occurs also after topical application *in vivo*, rapamycin may reduce the daily rate of hair shedding in these alopecias by inhibiting catagen development.

### mTORC1 inhibition stimulates melanogenesis in the human hair follicle pigmentary unit in a hair cycle‐*independent* manner

We next investigated by quantitative Masson–Fontana histomorphometry (Gáspár *et al*, 2011; Hardman *et al*, 2015) how rapamycin affects intrafollicular melanin production in pigmented human anagen VI scalp HFs *ex vivo*. This demonstrated that rapamycin treatment significantly increased melanin content within the HFPU compared with vehicle‐treated control HFs (Fig 2E and F). Rapamycin also stimulated tyrosinase activity *in situ*, the rate‐limiting enzyme of melanogenesis (Slominski *et al*, 2005), as visualized and quantified directly in the HFPU by fluorescent enzyme histochemistry (Han *et al*, 2002; Fig 2G and H). No tyrosinase activity was noted outside of the HFPU. Importantly, these analyses were performed only in HFs that were all in anagen VI, as verified by applying well‐defined hair cycle staging criteria (Fig 2C and D; Kloepper *et al*, 2010). Therefore, the pigmentation‐stimulatory effects of rapamycin were hair cycle‐*independent*.

The above data suggest that rapamycin impacts melanogenesis directly within the HFPU (Slominski *et al*, 2005; Tobin, 2011). Indeed, quantitative (immuno‐)histomorphometry for the premelanosomal marker gp100, which demarcates premelanomes and is a sensitive tracker of melanosome transfer between melanocytes and keratinocytes (Singh *et al*, 2008), showed that rapamycin significantly increased both the overall expression of gp100 protein within the HFPU (Fig 2I and J) and the dendricity of individual gp100‐positive melanocytes (Fig 2K and L). This indicates that mTORC1 inhibition also enhances melanosome transfer via melanocyte dendrites to keratinocytes of the precortical hair matrix (Tobin, 2011), thus altering the biology of HFPU melanocytes in a more complex manner than by just stimulating melanogenesis. Double‐immunostaining with Ki‐67 revealed that the pigmentation‐stimulatory effect of rapamycin was not mediated by an increase in the number (Fig EV1C and D) or proliferative activity of gp100‐positive HFPU melanocytes (Fig EV1E and F).

Therefore, rapamycin‐induced mTORC1 inhibition increases melanogenesis in the human HFPU primarily by (i) stimulating intrafollicular tyrosinase activity and (ii) increasing melanocyte dendricity, thus facilitating the transfer of melanin‐loaded melanosomes to a larger number of neighboring HF keratinocytes (Tobin, 2011; independent confirmation of this melanosome transfer effect by transmission electron microscopy would be desirable). Once—as yet unavailable—truly selective markers for human HF melanocyte stem cells have been identified, it will deserve systematic scrutiny if mTORC1 inhibition also promotes the intrafollicular differentiation of melanocyte stem cells located in the bulge and/or of amelanotic melanoblasts located in the periphery of the proximal hair bulb epithelium (Tobin, 2011; O'Sullivan *et al*, 2021).

### Rapamycin promotes intrafollicular melanogenesis by enhancing α‐MSH production and melanocortin‐1 receptor stimulation

Melanocyte‐inducing transcription factor (MITF) operates as the master regulator of pigmentation and drives the expression of tyrosinase, gp100, and other key genes involved in melanogenesis (Hida *et al*, 2020; Rachmin *et al*, 2020; Arora *et al*, 2021; Vu *et al*, 2021; Yardman‐Frank & Fisher, 2021; Zhou *et al*, 2021), also in human HFs (Nishimura *et al*, 2005; Gáspár *et al*, 2011; Samuelov *et al*, 2013; Hardman *et al*, 2015). *In vitro*, mTORC1 functions as an important regulator of MITF activity (Ohguchi *et al*, 2005; Ho *et al*, 2011; Yun *et al*, 2016; Slade & Pulinilkunnil, 2017; Napolitano *et al*, 2022). *In vitro*, rapamycin treatment of primary epidermal melanocytes silenced for TSC1/2, which is increasingly used in clinical TS management (Balestri *et al*, 2022), can restimulate melanin production, mainly by restoring MITF function and MITF target gene expression (Cao *et al*, 2017). While mTOR inhibition by rapamycin in cultured murine and human melanocytes or melanoma cells was shown to increase and/or restore MITF expression levels, promote the expression of MITF target genes such as *TYR*, *TYRP1*, and *PMEL*, and increase tyrosinase activity *in vitro* (Buscà *et al*, 1996; Ohguchi *et al*, 2005; Ho *et al*, 2011; Yun *et al*, 2016), active mTORC1 blocks the nuclear translocation of MITF (Roczniak‐Ferguson *et al*, 2012; Martina & Puertollano, 2013).

Therefore, we tested the plausible hypothesis that mTORC1 activity may regulate melanogenesis primarily via its impact on MITF. However, quantitative (immuno‐)histomorphometry showed that rapamycin does not significantly alter either total intrafollicular MITF protein levels or its phosphorylation in the human HFPU (Fig EV1G–J). This renders it unlikely that the pigmentation‐stimulatory effect of rapamycin in human HFs is primarily MITF‐controlled.

Given these unexpected findings, we next investigated whether melanocortin‐1 receptor (MC1R) activation by α‐MSH, the prototypic HF pigmentation‐stimulatory neurohormone (Paus, 2011; Paus *et al*, 2014; Swope & Abdel‐Malek, 2018), is involved in rapamycin‐induced HF pigmentation. α‐MSH is prominently produced by human HF keratinocytes *in vivo* and *ex vivo* (Ito *et al*, 2005; Kauser *et al*, 2005), while it is almost absent in graying/white HFs (O'Sullivan *et al*, 2021). Of note, α‐MSH can either stimulate melanogenesis indirectly via upregulating MITF (Hartman & Czyz, 2015) or p38/USF‐1 expression (Beaumont *et al*, 2011), or directly via inhibiting 6‐tetrahydrobiopterin (6BH(4)/7BH(4)), a known inhibitor of tyrosinase activity (Wood *et al*, 1995; Peters *et al*, 2000; Spencer *et al*, 2005).

Quantitative (immuno‐)histomorphometry showed that α‐MSH neuropeptide immunoreactivity was significantly upregulated within the HFPU of anagen VI HFs treated for 7 days with rapamycin *ex vivo* (Fig 2M and N). Mechanistically, this suggested that mTORC1 inhibition promotes tyrosinase‐driven melanogenesis and melanocyte dendrite formation, at least in part, by enhancing α‐MSH production by human hair matrix keratinocytes (Ito *et al*, 2005) and activating MC1R on HFPU melanocytes.

To probe this novel hypothesis, organ‐cultured human anagen VI HFs were treated for 7 days with rapamycin in the presence or absence of the specific MC1R antagonist, Agouti protein (also known as Agouti Signaling Protein; ASP; Böhm *et al*, 2005, 2012; Sharov *et al*, 2005; Jarrett *et al*, 2015). Unlike rapamycin, which downregulated S6 phosphorylation, Agouti alone or in combination with rapamycin did not influence mTORC1 activity (Fig 2O and P). Yet, MC1R blocking by Agouti blunted the pigmentation‐stimulatory effect of rapamycin (Fig 2Q and R). This strongly suggests that the melanogenic effect of rapamycin is largely mediated by stimulating the α‐MSH/MC1R pathway.

Mechanistically, the pigmentary effects thus appear to mainly result from an increased intrafollicular production of the key melanotropic neurohormone, α‐MSH, and subsequent stimulation of its cognate high‐affinity receptor, MC1R. It would be intriguing to assess in a follow‐up study whether rapamycin or TSC2 siRNA also impact the levels of MC1R protein expression and/or signaling activity. The upregulation of α‐MSH by rapamycin revealed here is clinically interesting beyond HF pigmentation: This immune‐inhibitory melanocortin also operates as a potent guardian of the HF's physiological immune privilege (Ito *et al*, 2004; Harries *et al*, 2013) and thereby protects the HF from immunologically mediated damage, while failure to do so results in inflammatory hair diseases like alopecia areata, lichen planopilaris, and frontal fibrosing alopecia (Harries *et al*, 2018; Gilhar *et al*, 2019; Bertolini *et al*, 2020). Moreover, α‐MSH can mitigate chemotherapy‐induced HF damage *ex vivo* (Böhm *et al*, 2014). Therefore, the pharmacological inhibition of mTORC1 activity may also be a promising novel strategy for enhancing the endogenous production of the potent anti‐inflammatory and tissue‐protective neurohormone, α‐MSH, within a human tissue. While our data do not formally rule out some contributory role of MITF, they suggest that mTORC1 activity does not control human HF pigmentation primarily via MITF, but mainly via α‐MSH/MC1R‐mediated signaling. Yet, the signaling pathway responsible for increased α‐MSH protein levels within the HF epithelium upon mTORC1 inhibition remains unknown to date.

These findings further caution against conceptually extrapolating from *in vitro* findings in isolated melanocytes to the regulatory controls these cells underlie within their physiological tissue habitat (Sevilla *et al*, 2022). In fact, melanocytes never operate in isolation, but typically are integral components of specialized pigmentary tissue units. Namely, the functional activities of melanocytes in the human HFPU appear to be largely controlled by hair matrix keratinocytes, dermal papilla fibroblasts, and even perifollicular adipocytes (Samuelov *et al*, 2013; Nicu *et al*, 2021; Tiede *et al*, 2021). Thus, our demonstration that, contrary to most of the published cell culture literature, the inhibition of mTORC1 activity primarily stimulates human HF melanocytes via α‐MSH/MC1R‐mediated signaling underscores the instructiveness and indispensability of human HF organ culture as a discovery tool for elucidating previously unknown *physiological* controls of human pigmentation and melanocyte function (Gáspár *et al*, 2011; Samuelov *et al*, 2013; Hardman *et al*, 2015; Nicu *et al*, 2021; Tiede *et al*, 2021).

### mTORC1 overactivation decreases human scalp hair follicle pigmentation *ex vivo*

**Figure 3:**
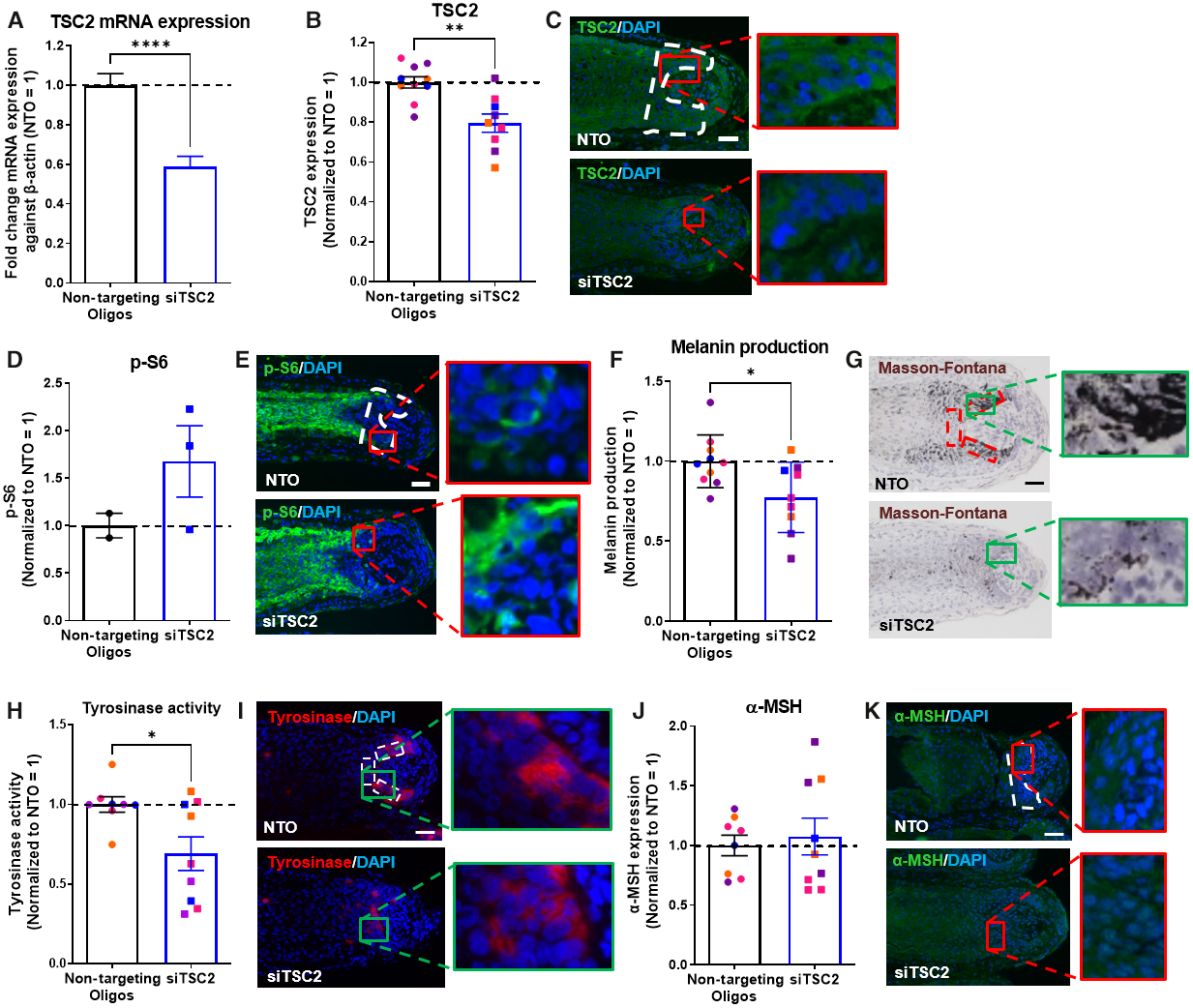
mTORC1 overactivation by TSC2 silencing *ex vivo* inhibits human scalp hair follicle pigmentation A, BQuantitative analysis of TSC2 mRNA (A) and protein expression (B). *N* = 8–10 anagen VI HFs from four different donors treated with siTSC2 or nontargeting oligos (NTO) for 6 days.CRepresentative images of TSC2 immunofluorescence.DQuantitative analysis of phosphorylated S6 (p‐S6; mTORC1 activity read‐out) immunoreactivity. *N* = 2–3 anagen VI HFs from one donor treated with siTSC2 or nontargeting oligos for 6 days.ERepresentative images of p‐S6 immunofluorescence.FQuantitative histomorphometry of melanin production. *N* = 9–10 anagen VI HFs from four different donors treated with siTSC2 or nontargeting oligos for 6 days.GRepresentative bright‐field microscopic images of Masson–Fontana histochemistry.HQuantitative analysis of tyrosinase activity. *N* = 8–9 anagen VI HFs from four different donors treated with siTSC2 or nontargeting oligos for 6 days.IRepresentative images of tyrosinase activity immunofluorescence.JQuantitative analysis of α‐MSH expression. *N* = 8–9 anagen VI HFs from four different donors treated with siTSC2 or nontargeting oligos for 6 days.KRepresentative images of α‐MSH immunofluorescence. Data information: Only anagen VI HFs were investigated and analyses performed in defined reference areas (dotted areas) in the HFPU. Mean ± SEM, Unpaired Student's *t*‐test, *P < 0.05, **P < 0.01, ****P < 0.0001. Scale bar: 50 μm. Samples from each donor are represented by a different color. Nuclei stained with DAPI. Source data are available online for this figure.

In order to generate additional, independent evidence that endogenous mTORC1 activity functions as an important inhibitor of human HF pigmentation, we also asked whether the specific overactivation of mTORC1 activity via silencing of its key upstream negative regulator, TSC2 (Demetriades *et al*, 2014; Wataya‐Kaneda, 2015; Rehbein *et al*, 2021; Curatolo *et al*, 2022; Ferreri *et al*, 2022), inhibits human HF pigmentation *ex vivo*. Using our well‐established gene silencing methodology in human HF organ culture (Samuelov *et al*, 2013; Hardman *et al*, 2015; Chéret *et al*, 2018; Tiede *et al*, 2021), TSC2 siRNA significantly reduced TSC2 mRNA and protein expression in the HFPU of human anagen VI HFs compared with transient *ex vivo* transfection with non‐targeting oligonucleotides (NTO; Fig 3A–C).

As expected, TSC2 silencing upregulated intrafollicular mTORC1 activity as documented by enhanced S6 phosphorylation (Fig 3D and E). Incidentally, this introduces a novel, previously unavailable preclinical assay system for functionally studying TSC physiology and pathobiology directly in an easily accessible and experimentally pliable human (mini‐)organ, the scalp HF, which also happens to be one of the prominent clinical signature target organs in TS (Apibal *et al*, 2008; Cartron *et al*, 2021). In this new TS research assay, the functional impact of reduced TSC2 activity on multiple epithelial, neural crest‐derived, and mesenchymal human cell populations can now be interrogated directly within a physiological cell–cell and tissue interaction context.

The moderate TSC2 protein knockdown efficiency of the employed methodology (which is expected during gene silencing in an intact organ) nevertheless sufficed to exert major functional effects: both intrafollicular melanogenesis (Fig 3F and G) and tyrosinase activity *in situ* (Fig 3H and I) were significantly decreased upon intrafollicular TSC2 knockdown. Given the relatively modest knockdown efficiency, this highlights how tightly mTORC1 activity is regulated by TSC2 in human scalp HFs.

**Figure EV2:**
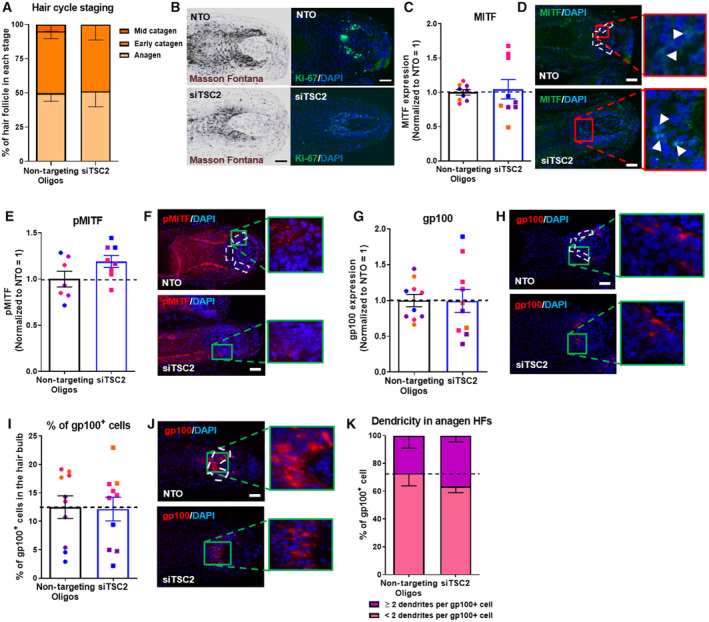
mTORC1 overactivation does not affect melanocyte dendricity and number in human scalp hair follicles Hair cycle staging was performed using Ki‐67 and Masson–Fontana histochemistry. Mean ± SEM; *N* = 21–22 HFs per group from four different donors treated with siTSC2 or nontargeting oligos for 6 days; Unpaired Student's *t*‐test.Representative fluorescence images of Ki‐67 and bright‐field microscopic images of Masson–Fontana.Quantitative analysis of MITF expression. *N* = 9 anagen VI HFs from four different donors treated with siTSC2 or nontargeting oligos (NTO) for 6 days.Representative images of MITF immunofluorescence. White arrows show MITF^+^ cells.Quantitative analysis of MITF phosphorylation (pMITF). *N* = 8 anagen VI HFs from four different donors treated with siTSC2 or nontargeting oligos for 6 days.Representative images of pMITF immunofluorescence.Quantitative analysis of gp100 expression. *N* = 10 anagen VI HFs from four different donors treated with siTSC2 or nontargeting oligos for 6 days.Representative images of gp100 immunofluorescence.Quantitative analysis of gp100^+^ cell number. *N* = 9 anagen VI HFs from four different donors treated with siTSC2 or nontargeting oligos for 6 days.Representative images of gp100 immunofluorescence.Quantitative analysis of melanocyte dendricity. *N* = 10 anagen VI HFs from four different donors treated with siTSC2 or nontargeting oligos for 6 days. Data information: Only anagen VI HFs (except for A and B where all HFs were analyzed) were investigated and analyses performed in defined reference areas (dotted areas) in the HFPU. Mean ± SEM, Student's *t*‐test. Scale bar: 50 μm. Samples from each donor represented by a different color. Nuclei stained with DAPI.

However, the relatively short period of TSC2 silencing *ex vivo* (6 days) did not suffice to induce significant changes in intrafollicular α‐MSH protein expression levels, total gp100 immunoreactivity, melanocyte number, proliferation and dendricity, or pMITF and MITF (Figs 3J and K, and EV2C–K) or HF cycling/anagen duration (Fig EV2A and B). Longer‐term mTORC1 overactivation may be required to significantly alter these read‐outs. Yet, the observed downregulation of melanin production and tyrosinase activity in the HFPU by TSC2 knockdown *ex vivo* (Fig 3F–I) independently confirmed that human HF pigmentation is negatively controlled by mTORC1 activity, which, in turn, is kept in check by TSC2. This provides a plausible, previously unavailable mechanistic explanation for the characteristic hair depigmentation (poliosis) seen in TS patients (Wataya‐Kaneda, 2015; Cartron *et al*, 2021; Takahashi *et al*, 2022).

### Rapamycin may prime selected white HFs, in which graying is still reversible, for repigmentation

Contrary to a widespread and often reverberated misconception, human hair graying does not primarily result from the depletion of HF melanocyte stem cells, but is initiated by a dysfunction of differentiated melanocytes in the HFPU (Paus, 2011; O'Sullivan *et al*, 2021; Rosenberg *et al*, 2021); ultimately, human hair greaying becomes irreversible when HF melanocyte stem cells have become depleted (Nishimura *et al*, 2005; Nishimura, 2011). Human scalp HF depigmentation is also more reversible than widely appreciated (O'Sullivan *et al*, 2021; Rosenberg *et al*, 2021), and white/graying HFs can contain some residual melanin‐producing HF melanocytes (Arck *et al*, 2006; O'Sullivan *et al*, 2021). Therefore, we finally probed whether rapamycin treatment can also restimulate pigmentation in some gray/white HFs, despite the very short duration of HF organ culture (note that, clinically, spontaneous or drug/hormone‐induced repigmentation of gray/white hair typically takes weeks or months; O'Sullivan *et al*, 2021; Rosenberg *et al*, 2021). We focused on the analysis of those gray/white HFs that still had at least one gp100‐positive cell/HF present in their HFPU, as visualized by gp100 immunofluorescence microscopy, which permits one to detect even melanocytes that are not yet engaged in full melanin synthesis (Singh *et al*, 2008).

**Figure 4:**
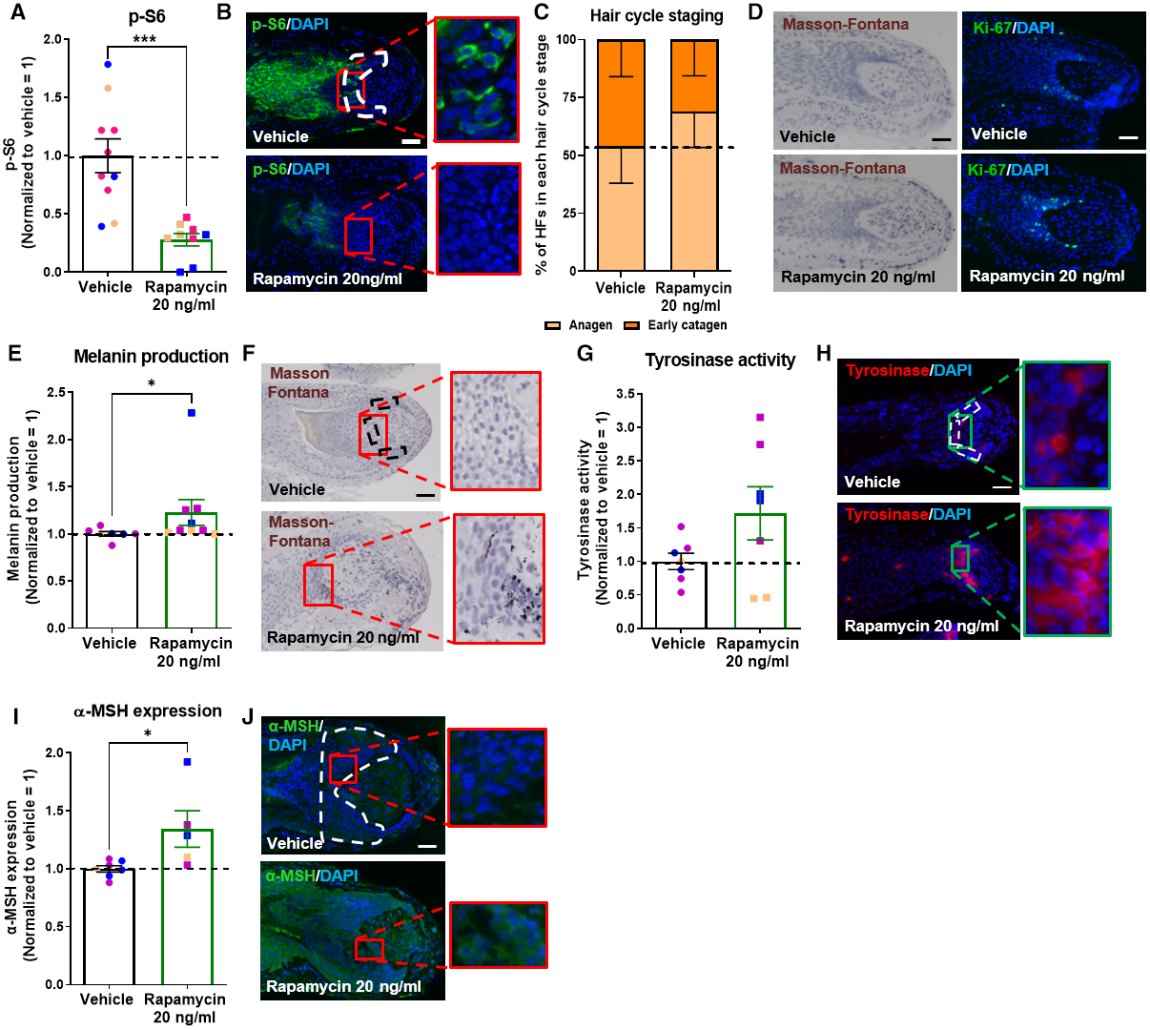
Rapamycin stimulates repigmentation‐associated parameters in gray/white anagen scalp hair follicles of responding donors Quantitative analysis of phosphorylated S6 (p‐S6; mTORC1 activity read‐out) immunoreactivity. *N* = 9–10 gray anagen VI HFs from three different donors treated with Rapamycin 20 ng/ml or untreated (vehicle) for 7 days.Representative fluorescence images of p‐S6 immunofluorescence.Hair cycle staging was performed using Ki‐67 and Masson–Fontana histochemistry. Mean ± SEM; *N* = 16–17 gray HFs per group from three different donors treated with Rapamycin 20 ng/ml or untreated (vehicle) for 7 days.Representative fluorescence images of Ki‐67 and bright‐field microscopic images of Masson–Fontana.Quantitative histomorphometry of melanin production by Masson–Fontana histochemistry. *N* = 7–9 gray anagen VI HFs from three different donors treated with Rapamycin 20 ng/ml or untreated (vehicle) for 7 days.Representative pictures of Masson–Fontana histochemistry.Quantitative analysis of tyrosinase activity. *N* = 7 gray anagen VI HFs from three different donors treated with Rapamycin 20 ng/ml or untreated (vehicle) for 7 days.Representative images of tyrosinase activity immunofluorescence.Quantitative analysis of α‐MSH expression in defined reference area within the bulb. *N* = 5–7 gray anagen VI HFs from three different donors treated with Rapamycin 20 ng/ml or untreated (vehicle) for 7 days.Representative images of α‐MSH immunofluorescence. Data information: Only anagen VI HFs (except for C and D where all HFs were analyzed) were investigated and analyses performed in defined reference areas (dotted areas) in the HFPU. Mean ± SEM, Student's *t*‐test (A, C) or Mann–Whitney U‐test (E, G, I), *P < 0.05, ***P < 0.001. Scale bar: 50 μm. Samples from each donor represented by a different color. Nuclei stained with DAPI. Source data are available online for this figure.

**Figure EV3:**
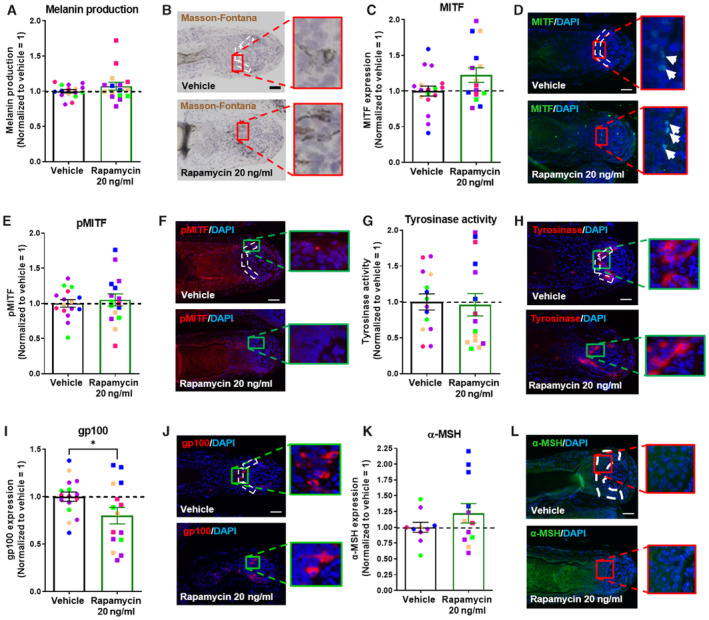
mTORC1 inhibition stimulates repigmentation of gray/white human scalp hair follicles only in certain hair follicles Quantitative histomorphometry of melanin production by Masson–Fontana histochemistry in defined reference area in the bulb. *N* = 16 gray anagen VI HFs from five different donors treated with Rapamycin 20 ng/ml or untreated (vehicle) for 7 days.Representative bright‐field microscopy images of Masson–Fontana histochemistry.Quantitative analysis of MITF expression in defined reference area in the bulb. *N* = 15–17 gray anagen VI HFs from five different donors treated with Rapamycin 20 ng/ml or untreated (vehicle) for 7 days.Representative images of MITF immunofluorescence. White arrows showed MITF^+^ cells.Quantitative analysis of MITF phosphorylation (pMITF) in defined reference area in the bulb. *N* = 16 gray anagen VI HFs from five different donors treated with Rapamycin 20 ng/ml or untreated (vehicle) for 7 days.Representative fluorescence images of pMITF immunofluorescence. White arrows showed pMITF^+^ cells.Quantitative analysis of tyrosinase activity in defined reference area in the bulb. *N* = 12–13 gray anagen VI HFs from four different donors treated with Rapamycin 20 ng/ml or untreated (vehicle) for 7 days.Representative images of tyrosinase activity immunofluorescence.Quantitative analysis of gp100 expression in defined reference area within the bulb. *N* = 15–16 gray anagen VI HFs from five different donors treated with Rapamycin 20 ng/ml or untreated (vehicle) for 7 days.Representative images of gp100 immunofluorescence.Quantitative analysis of α‐MSH expression in defined reference area within the bulb. *N* = 10–12 gray anagen VI HFs from five different donors treated with Rapamycin 20 ng/ml or untreated (vehicle) for 7 days.Representative images of α‐MSH immunofluorescence. Data information: Only anagen VI HFs were investigated and analyses performed in defined reference areas (dotted areas) in the HFPU. Mean ± SEM, Mann–Whitney U‐test (A) or Student's *t*‐test (C, E, G, I, K), *P < 0.05. Scale bar: 50 μm. Samples from each donor represented by a different color. Nuclei stained with DAPI.

**Figure EV4:**
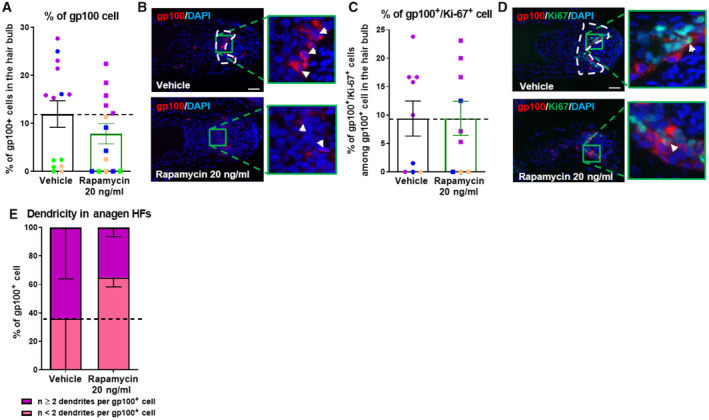
mTORC1 inhibition does not significantly alter melanocyte number, proliferation state, and dendricity in gray/white human scalp gray hair follicles Quantitative immunohistomorphometry of the number of gp100^+^ cells. *N* = 14 gray anagen VI HFs from four different donors treated with Rapamycin 20 ng/ml or untreated (vehicle) for 7 days.Representative images of gp100 immunofluorescence. Arrows indicate gp100^+^ cells.Quantitative analysis of gp100^+^/ki‐67^+^ cell number. *N* = 9 gray anagen VI HFs from three different donors treated with Rapamycin 20 ng/ml or untreated (vehicle) for 7 days.Representative images of gp100/ki‐67 immunofluorescence. Arrows indicate gp100^+^Ki‐67^+^ cells.Quantitative analysis of melanocyte dendricity. *N* = 10–12 gray anagen VI HFs from four different donors treated with Rapamycin 20 ng/ml or untreated (vehicle) for 7 days. Data information: Only anagen VI HFs were investigated and analyses performed in defined reference areas (dotted areas) in the HFPU. Mean ± SEM, Mann–Whitney U‐test. Scale bar: 50 μm. Samples from each donor represented by a different color. Nuclei stained with DAPI.

When these selected gray/white HFs were stimulated for only 7 days with rapamycin, this significantly decreased S6 phosphorylation in the bulb, demonstrating successful mTORC1 inhibition (Fig 4A and B). Furthermore, rapamycin tended to slightly, but not significantly prolong anagen in these gray/white HFs (Fig 4C and D). Interestingly, when only HFs from *responding donors* (characterized by increased intrafollicular melanin production and at least one gp100‐positive cell detected in the HFPU) were pooled, rapamycin treatment demonstrated a remarkable upregulation of melanin production, tyrosinase activity, and α‐MSH protein content in these selected responder HFs, even within the short time window of HF organ culture (Figs 4E–J, EV3A–L and EV4A–E for the analyses of pooled gray/white HFs from both responders and nonresponder HFs).

Although the low sample number of gray/white HFs and donors that could be included in this study requires repetition in larger HF populations and additional donors, this provides the first evidence that inhibiting intrafollicular mTORC1 activity can indeed partially restimulate melanogenesis in selected graying/white responder HFs, likely when a few functional HFPU melanocytes are still present and HF depigmentation has not yet progressed beyond the point of irreversibility (O'Sullivan *et al*, 2021; Rosenberg *et al*, 2021). This begs the clinically crucial, as yet open question what exactly determines whether or not a graying/white HF can still respond to mTORC1 inhibition with repigmentation.

Taken together, the present study provides conclusive evidence in support of the working hypothesis that human HF pigmentation is negatively regulated by mTORC1 activity, that mTORC1 activity regulates the α‐MSH/MC1R pathway, and that mTORC1 inhibition by rapamycin can partially restimulate pigmentation even in selected graying/white HF. Vice versa, the latter exhibit excessive mTORC1 activity, while experimentally induced mTORC1 hyperactivation by TSC2 silencing significantly inhibits HF melanogenesis *ex vivo*. Moreover, mTORC1 inhibition by rapamycin also prolongs the phase of active hair growth (anagen). As an important methodological spin‐off, our study in addition reports a novel preclinical assay system for studying TS physiology and pathobiology by TSC2 silencing directly in a human model (mini‐)organ.

Translationally, our data strongly encourage one to next probe in a clinical trial whether topical rapamycin can significantly retard the graying process or even repigment graying/white hair, namely in younger individuals with recent onset of hair graying, and whether this indeed prolongs anagen duration *in vivo*. That topical rapamycin is already frequently used in daily dermatological practice (Mercurio *et al*, 2021; Balestri *et al*, 2022; Effendi *et al*, 2022) facilitates its repurposing for canities and poliosis management, provided that a sufficient level of rapamycin reaches the HFPU of graying/white human anagen HFs.

## Materials and Methods

### Tissue specimens

Human scalp HFs were obtained from a total of 22 healthy donors (male and female, 24–78 years old) undergoing routine face‐lift surgery/hair transplantation. The use of anonymized, discarded human tissue was considered to represent non‐human research and exempted under 45 CFR46.101.2 by the IRB of the University of Miami Miller School of Medicine.

### HF organ culture

Human scalp HFs or scalp skin samples were collected on Day 0 or 1 after a face‐lifting procedure. Anagen VI scalp HFs were selected and microdissected on the same day of tissue receipt in the laboratory. Microdissected human scalp HFs were cultured at 37°C with 5% CO_2_ in William's E media (WEM, Gibco, Life Technologies) supplemented with 2 mM of L‐glutamine (Gibco), 10 ng/ml hydrocortisone (Sigma‐Aldrich), 10 μg/ml insulin (Sigma‐Aldrich), 1% amphotericin B (Gibco), and 1% penicillin/streptomycin mix (Gibco; Langan *et al*, 2015; Edelkamp *et al*, 2020). After microdissection, HFs were first preincubated in supplemented WEM for 24 h for re‐equilibration, given that human HFs undergo a substantial, but temporary stress response *ex vivo* after microdissection (Uchida *et al*, 2021). Following quality control (HFs not morphologically damaged and all in anagen VI), pigmented or gray/white HFs were randomly allocated to the different experimental groups. Rapamycin (final concentration in each rapamycin‐treated well: 20 ng/ml, Santa Cruz, sc‐3504), agouti signaling protein (ASIP)/agouti protein (final concentration in each agouti‐treated well: 2 μg/ml, R&D system, 9094‐AG) or vehicle (final solvent concentration in each vehicle‐treated well: 0.1% DMSO; Sigma‐Aldrich) was administrated on Days 1, 3, and 5 during medium change. At the end of the culture, HFs were embedded in optimal cutting temperature compound (OCT) and snap‐frozen in liquid nitrogen.

### siRNA transfection‐mediated knockdown of TSC2 in organ‐cultured HFs

Human anagen VI HFs were transfected with siRNA probe (ON‐TARGETplus Human TSC2 [7249] siRNA—SMARTpool, 5 nmol [siTSC2; Horizon Discovery Ltd, cat. L‐003029‐00‐0005] or nontargeting oligos [NTO] [ON‐TARGETplus Non‐targeting Pool, 5 nmol]) using Lipofectamine™ RNAiMAX (Life Technologies) following the manufacturer's instructions (Life Technologies). In brief, final concentration of 1 μM of siTSC2 or siRNA control (NTO) was added 24 h after microdissection for 48 h in each of the corresponding wells. Three HFs were retrieved and frozen for RNA extraction and subsequent quantitative reverse transcription polymerase chain reaction qRT–PCR, 48 h after transfection. After additional 72 h of culture, the remaining HFs were OCT‐embedded, snap‐frozen in liquid nitrogen, and cut into 6 μm sections for (immuno‐)histology.

### Quantitative reverse transcription‐PCR

Total RNA was isolated from whole microdissected HFs using PicoPure™ RNA Isolation Kit (Applied Biosystems—ThermoFisher) following the manufacturer's instructions (Mardaryev *et al*, 2021). RNA purity and concentrations were determined using the Nanodrop ND‐1000 assay (Fisher Scientific). Reverse transcription of the RNA into cDNA was performed using the TetrocDNA Synthesis Kit (Bioline‐Meridian Bioscience), according to the manufacturer's instructions. The RNA concentrations were adjusted (between 50 and 500 nM) in order to have the same amount of RNA among the same donor to allow further quantification and comparison between the samples after real‐time qRT–PCR. Normalization was performed using the housekeeping gene ACTB. qRT–PCR was run in triplicate using TaqMan Fast Advanced Master Mix and Gene Expression Assay probes (Id: Hs99999903_m1 for ACTB, and Hs01020387_m1 for TSC2, Thermo scientific) on the qTower2.2 thermocycler. Real‐time quantification plots and Ct values were collected and stored by the qPCRsoft2.1 software. The amount of the transcripts was normalized to those of the housekeeping gene (ACTB) using the ΔΔCT method (Chéret *et al*, 2018; Hawkshaw *et al*, 2018; Mardaryev *et al*, 2021).

### Immunofluorescence staining

OCT‐embedded samples were sectioned (6 μm thickness) with a Cryostar NX50 (Thermo scientific). Briefly, after fixation (see Table 1), cryosections were preincubated for 30 min at RT (see Table 1) followed by primary antibody (see Table 1) incubation overnight at 4°C. After three times 5‐min‐long washes, slides were incubated with the corresponding fluorescent‐labeled secondary antibody for 45 min at room temperature (RT).

**Table 1:** List of fixation, blocking and primary and secondary antibodies.

Antigen	Fixation	Blocking	Primary ab	Secondary ab
Ki‐67/gp100	4% PFA, 10 min at RT	10% goat serum in PBS	Mouse anti‐Ki‐67 (1:800; 9449S; cell signaling) and rabbit anti‐NKI‐beteb/gp100 (1:100, ab137078, Abcam)	FITC‐labeled goat anti‐mouse antibody (Jackson ImmunoResearch, 1:200) and NKI‐beteb/gp100 with a goat‐anti‐rabbit Alexa Fluor® 555 antibody (Life technology, 1:400)
MITF	Methanol:acetone (1:1), 10 min at −20°C	10% Goat serum in TBS + 0.3% Triton X‐100	Mouse anti‐MITF (1:50, Abcam, ab12039)	FITC‐labeled goat anti‐mouse antibody (Jackson ImmunoResearch, 1:200)
pMITF	Methanol:acetone (1:1), 10 min at −20°C	5% BSA in TBS	Rabbit anti‐Anti‐phospho‐MITF (1:50, Sigma‐Aldrich, SAB4503940)	Goat‐anti‐rabbit Alexa Fluor® 555 antibody (Life technology, 1:400)
p‐S6	4% PFA, 10 min at RT	N/A	Rabbit anti‐Phospho‐S6 Ribosomal Protein (Ser235/236; 1:200; 4858; cell signaling)	Goat‐anti‐rabbit Alexa Fluor® 488 antibody (Life technology, 1:400)
TSC2	4% PFA, 10 min at RT	10% BSA in PBS	Rabbit anti‐Tuberin/TSC2 (1:100, Cell signaling, 4308S)	FITC‐labeled goat anti‐rabbit antibody (Jackson ImmunoResearch, 1:200) followed by an amplification with Alexa Fluor® 488 Anti‐FITC (Life technology, 1:700)
α‐MSH	Acetone, 10 min at −20°C	2% BSA in PBS	Rabbit anti‐alpha‐MSH (Millipore Sigma, M0939), 1:500	FITC‐labeled goat anti‐rabbit antibody (Jackson ImmunoResearch, 1:200) followed by an amplification with Alexa Fluor® 488 Anti‐FITC (Life technology, 1:700)

For assessing tyrosinase activity *in situ* (Samuelov *et al*, 2013; Hardman *et al*, 2015; Chéret *et al*, 2020), sections were fixed in methanol:acetone (1:1) for 10 min at −20°C, followed by three times 5‐min‐long washes in PBS before blocking the endogenous peroxidase in 3% H_2_O_2_ in PBS for 15 min. After additional three times 5‐min‐long washes in PBS, the sections were pretreated for 15 min each at RT in Avidin/Biotin (Vector Labs). Three times 5‐min‐long washes in PBS were then followed by preincubation for 30 min at RT in 5% normal goat serum +1% BSA in PBS and then in Biotinylated TSA‐Reagent (1:50, NEL700A001KT, Perkin Elmer) for 30 min at RT. After three times 5‐min‐long washes in 0.1% IGEPAL^®^ (I3021, Sigma‐Aldrich) in PBS, sections were incubated with the primary antibody Streptavidin‐Cy3 (1:600, S6402, Sigma‐Aldrich), followed by three times 5‐min‐long washes in 0.1% IGEPAL^®^ in PBS. Finally, cryosections were counterstained with 4′,6′‐diamidino‐2‐phenylindole dihydrochloride (DAPI).

Of note, gp100 (=demarcates premelanomes) is a well‐recognized and sensitive tracker of melanosome transfer between melanocytes and keratinocytes (Singh *et al*, 2008).

### Histochemistry

Masson–Fontana staining was carried out to assess the melanin content of the HFs according to the previously established protocol (Samuelov *et al*, 2013; Hardman *et al*, 2015; Chéret *et al*, 2020).

### Microscopy, image analysis, and quantitative (immuno‐)histomorphometry

Images were acquired using the BZ‐X800 All‐in‐one Fluorescence Microscope (Keyence Corporation, Osaka, Japan) maintaining a constant set exposure time throughout imaging. Images were taken at 200× magnifications. Image analysis and measurement of staining intensity was evaluated in well‐defined reference areas (see vehicle pictures [dotted area]) using NIH ImageJ software (NIH, Bethesda, Maryland, USA), as previously described (Gáspár *et al*, 2011; Hardman *et al*, 2015; Chéret *et al*, 2020; Tiede *et al*, 2021). In brief, for measuring the staining intensity of our different markers, we first draw the reference area (see the dotted area on each figure) and measure the mean intensity. For analyzing the number of single‐ and double‐positive cells, we have used the cell counter functions of ImageJ and individually pointed the positive (single and/or double).

### Statistical analyses

All data are expressed as fold change of mean or mean ± SEM and were analyzed by Student's *t*‐test or Mann–Whitney test for data following Gaussian and non‐Gaussian distribution, respectively (Graph Pad Prism 9, GraphPad Software, San Diego, CA, USA) after performing d'Agostino and Pearson omnibus normality test. *P* < 0.05 was regarded as significant.

## Author contributions

**Takahiro Suzuki:** Data curation; formal analysis; writing – review and editing. **Jérémy Chéret:** Conceptualization; data curation; formal analysis; writing – original draft. **Fernanda Dinelli Scala:** Data curation; formal analysis; writing – review and editing. **Aysun Akhundlu:** Data curation; formal analysis; writing – review and editing. **Jennifer Gherardini:** Data curation; formal analysis; writing – review and editing. **Dana‐Lee Demetrius:** Data curation; formal analysis; writing – review and editing. **James D B O'Sullivan:** Data curation; formal analysis; writing – review and editing. **Gorana Kuka Epstein:** Resources; writing – review and editing. **Alan J Bauman:** Resources; writing – review and editing. **Constantinos Demetriades:** Conceptualization; writing – review and editing. **Ralf Paus:** Conceptualization; supervision; writing – original draft.

## Disclosure and competing interests statement

The authors declare that they have no conflict of interest. For the record, RP and JC and JG were or are employees of Monasterium Laboratory, a skin & hair research CRO (www.monasteriumlab.com) that provides HF research services, and RP has founded and JC consults for a company (www.cutaneon.com) that develops hair growth/pigmentation‐modulatory treatment strategies.

## Supporting information



Expanded View Figures PDF

Source Data for Expanded View

PDF+

Source Data for Figure 1

Source Data for Figure 2

Source Data for Figure 3

Source Data for Figure 4

## Data Availability

This study includes no data deposited in external repositories. The authors declare that all data supporting the findings of this study are available within this article and its supplementary materials or from the corresponding author upon reasonable request.
