# Supplementary material for: mTORC1 activity negatively regulates human hair follicle growth and pigmentation
Source: EMBO Rep. 2023 May 22;24(7):e56574. doi: 10.15252/embr.202256574 (PMC10328083; doi:10.15252/embr.202256574)
Supplement: Supplementary file 1 — Expanded View Figures PDF [file EMBR-24-e56574-s001.pdf]

Expanded View Figures

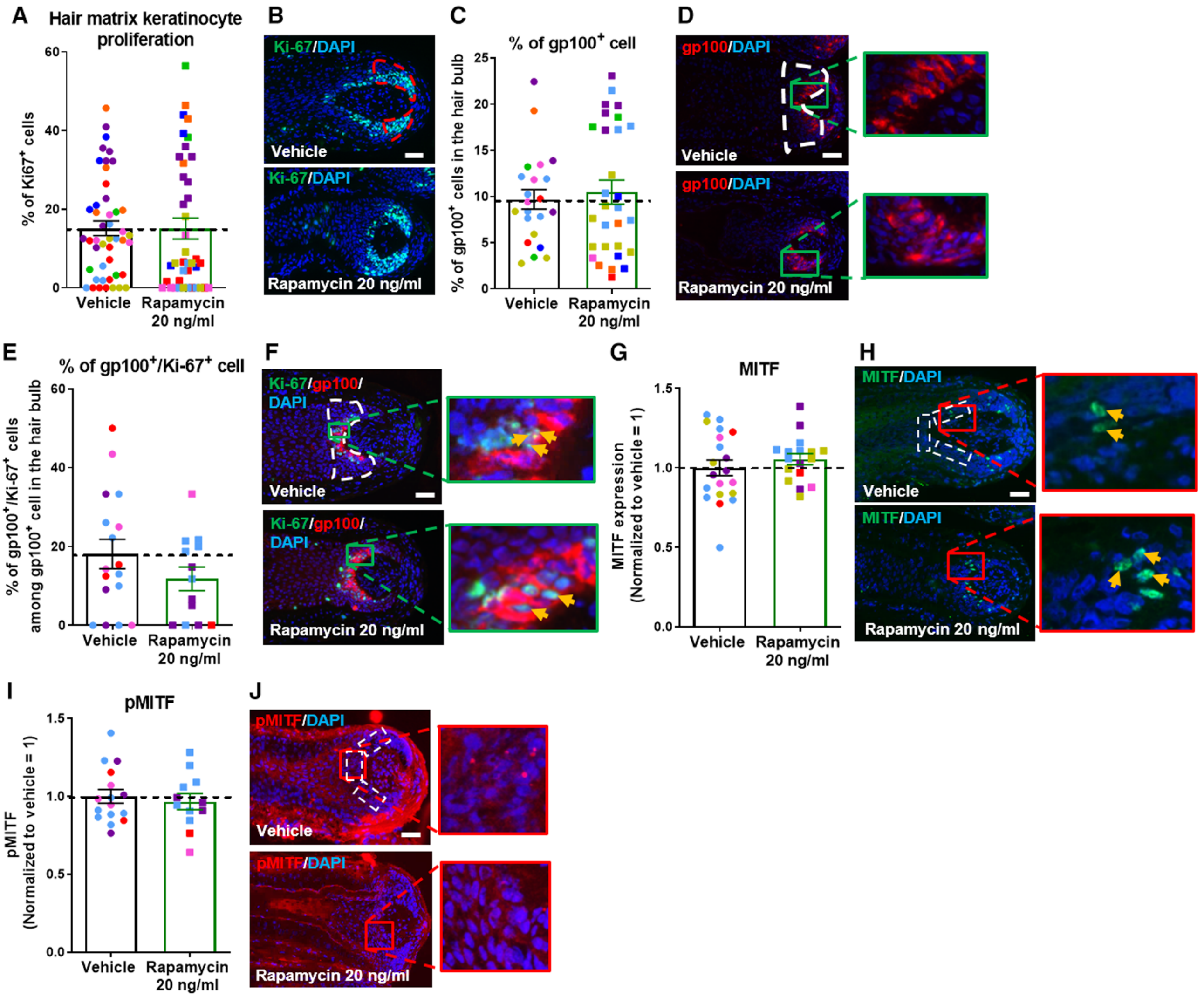

Figure EV1. mTORC1 inhibition did not affect MITF expression or the number and proliferation state of melanocytes in human scalp hair follicles.

- A Quantitative analysis of Ki-67$^+$ cell number. N = 40–48 anagen VI HFs from eight different donors treated with Rapamycin 20 ng/ml or untreated (vehicle) for 7 days.
- B Representative images of Ki-67 immunofluorescence.
- C Quantitative immunohistomorphometry of the number of gp100$^+$ cells. N = 22–29 anagen VI HFs from eight different donors treated with Rapamycin 20 ng/ml or untreated (vehicle) for 7 days.
- D Representative images of gp100 immunofluorescence.
- E Quantitative analysis of gp100$^+$/Ki-67$^+$ cell number. N = 13–17 anagen VI HFs from four different donors treated with Rapamycin 20 ng/ml or untreated (vehicle) for 7 days.
- F Representative images of gp100/Ki-67 immunofluorescence. Yellow arrows indicate gp100$^+$/Ki-67$^+$ cells.
- G Quantitative analysis of MITF expression. N = 17–20 anagen VI HFs from five different donors treated with Rapamycin 20 ng/ml or untreated (vehicle) for 7 days.
- H Representative images of MITF immunofluorescence. Yellow arrows indicate MITF$^+$ cells.
- I Quantitative analysis of MITF phosphorylation (pMITF). N = 12–16 anagen VI HFs from four different donors treated with Rapamycin 20 ng/ml or untreated (vehicle) for 7 days.
- J Representative images of pMITF immunofluorescence.

Data information: Only anagen VI HFs (except for A and B where all HFs were analyzed) were investigated and analyses performed in defined reference areas (dotted areas) in the HFPU. Mean ± SEM, Student's *t*-test (A, C, G, I) or Mann–Whitney U-test (E). Scale bar: 50 μm. Samples from each donor represented by a different color. Nuclei stained with DAPI.
Source data are available online for this figure.

© 2023 The Authors

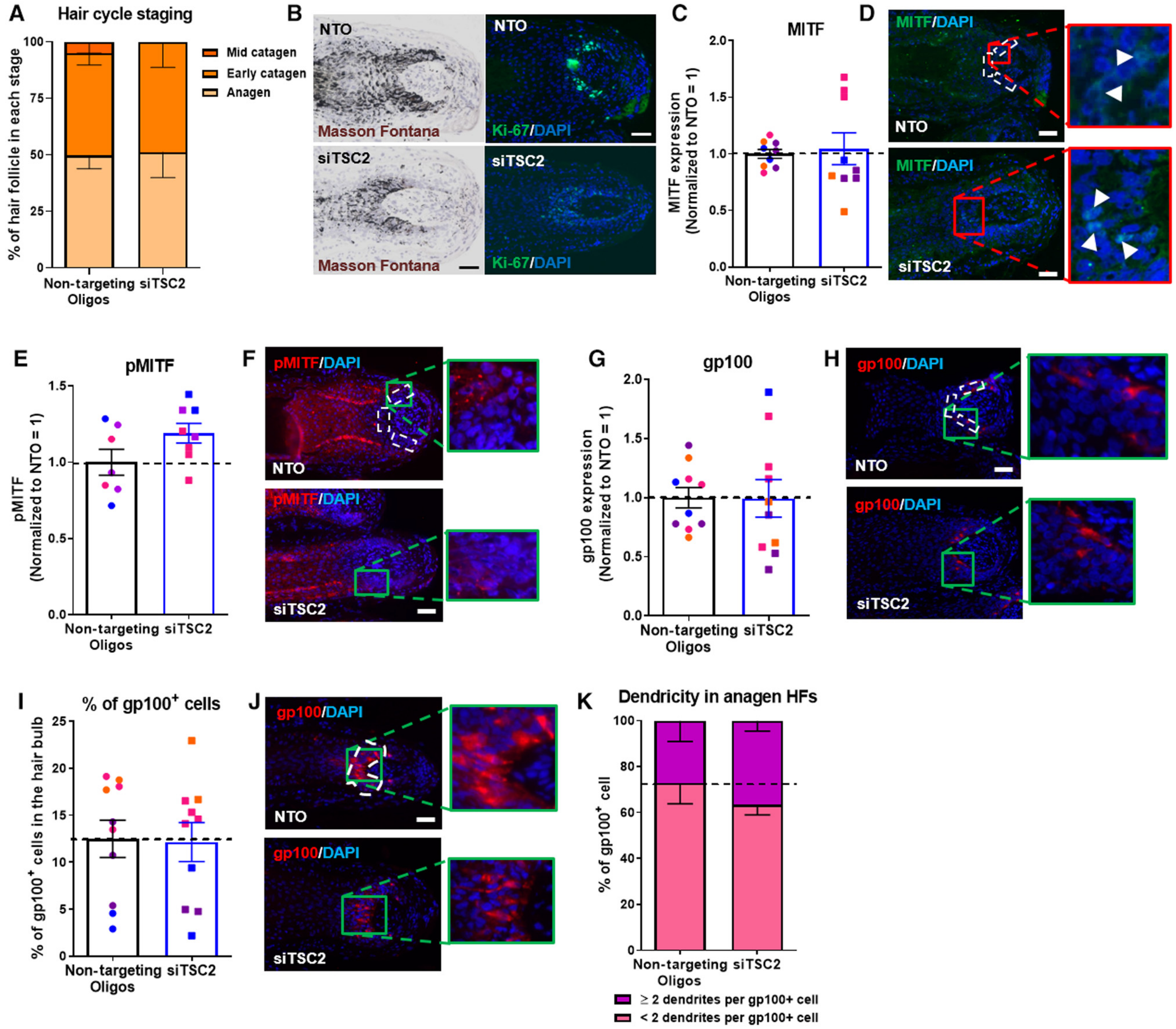

Figure EV2. mTORC1 overactivation does not affect melanocyte dendricity and number in human scalp hair follicles.

A Hair cycle staging was performed using Ki-67 and Masson–Fontana histochemistry. Mean ± SEM; $N = 21$–22 HFs per group from four different donors treated with siTSC2 or nontargeting oligos for 6 days; Unpaired Student's *t*-test.

B Representative fluorescence images of Ki-67 and bright-field microscopic images of Masson–Fontana.

C Quantitative analysis of MITF expression. $N = 9$ anagen VI HFs from four different donors treated with siTSC2 or nontargeting oligos (NTO) for 6 days.

D Representative images of MITF immunofluorescence. White arrows show MITF$^+$ cells.

E Quantitative analysis of MITF phosphorylation (pMITF). $N = 8$ anagen VI HFs from four different donors treated with siTSC2 or nontargeting oligos for 6 days.

F Representative images of pMITF immunofluorescence.

G Quantitative analysis of gp100 expression. $N = 10$ anagen VI HFs from four different donors treated with siTSC2 or nontargeting oligos for 6 days.

H Representative images of gp100 immunofluorescence.

I Quantitative analysis of gp100$^+$ cell number. $N = 9$ anagen VI HFs from four different donors treated with siTSC2 or nontargeting oligos for 6 days.

J Representative images of gp100 immunofluorescence.

K Quantitative analysis of melanocyte dendricity. $N = 10$ anagen VI HFs from four different donors treated with siTSC2 or nontargeting oligos for 6 days.

Data information: Only anagen VI HFs (except for A and B where all HFs were analyzed) were investigated and analyses performed in defined reference areas (dotted areas) in the HFPU. Mean ± SEM, Student's *t*-test. Scale bar: 50 μm. Samples from each donor represented by a different color. Nuclei stained with DAPI.

 © 2023 The Authors

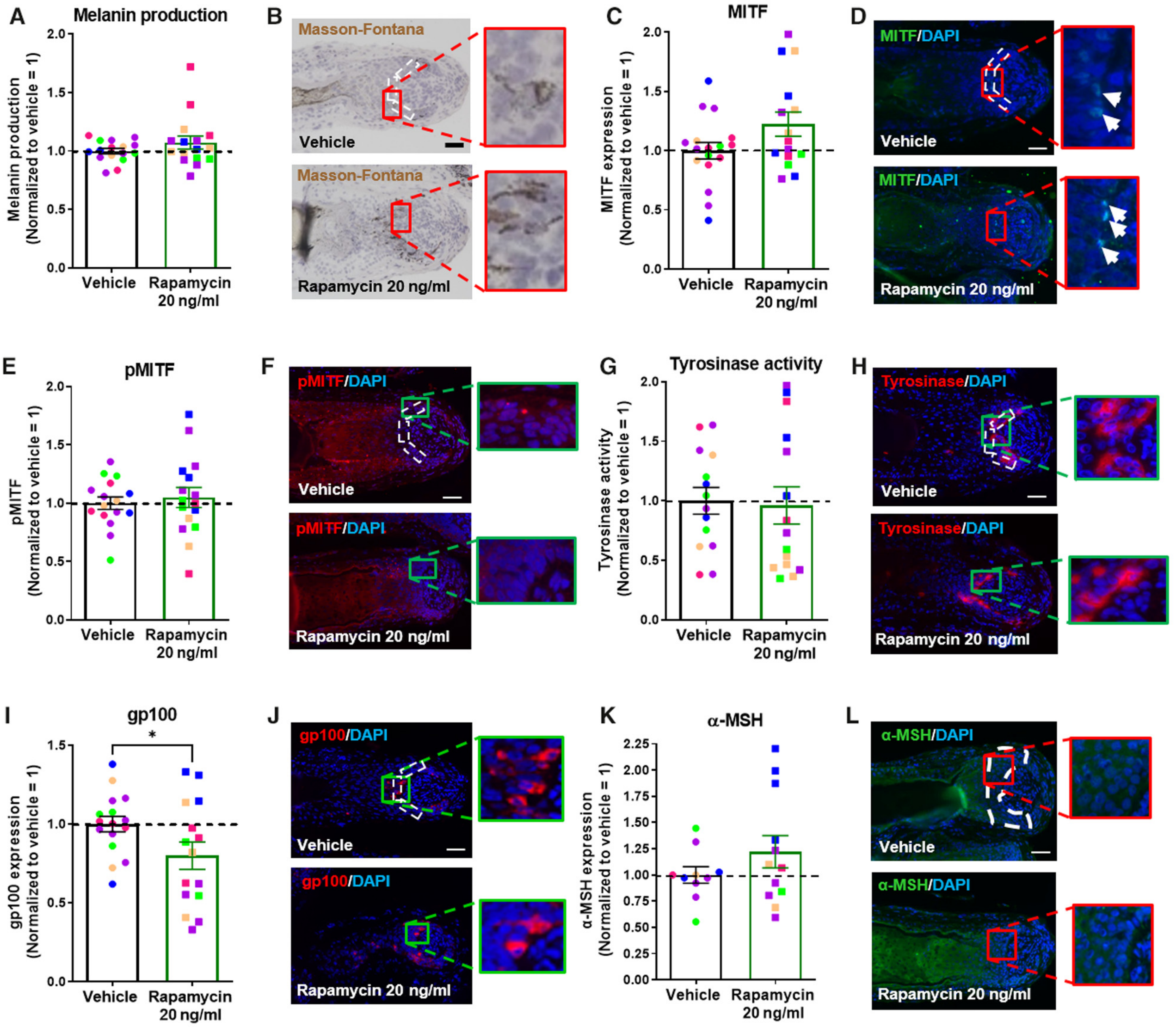

Figure EV3. mTORC1 inhibition stimulates repigmentation of gray/white human scalp hair follicles only in certain hair follicles.

A Quantitative histomorphometry of melanin production by Masson–Fontana histochemistry in defined reference area in the bulb. $N = 16$ gray anagen VI HFs from five different donors treated with Rapamycin 20 ng/ml or untreated (vehicle) for 7 days.

B Representative bright-field microscopy images of Masson–Fontana histochemistry.

C Quantitative analysis of MITF expression in defined reference area in the bulb. $N = 15$–17 gray anagen VI HFs from five different donors treated with Rapamycin 20 ng/ml or untreated (vehicle) for 7 days.

D Representative images of MITF immunofluorescence. White arrows showed MITF$^+$ cells.

E Quantitative analysis of MITF phosphorylation (pMITF) in defined reference area in the bulb. $N = 16$ gray anagen VI HFs from five different donors treated with Rapamycin 20 ng/ml or untreated (vehicle) for 7 days.

F Representative fluorescence images of pMITF immunofluorescence. White arrows showed pMITF$^+$ cells.

G Quantitative analysis of tyrosinase activity in defined reference area in the bulb. $N = 12$–13 gray anagen VI HFs from four different donors treated with Rapamycin 20 ng/ml or untreated (vehicle) for 7 days.

H Representative images of tyrosinase activity immunofluorescence.

I Quantitative analysis of gp100 expression in defined reference area within the bulb. $N = 15$–16 gray anagen VI HFs from five different donors treated with Rapamycin 20 ng/ml or untreated (vehicle) for 7 days.

J Representative images of gp100 immunofluorescence.

K Quantitative analysis of α-MSH expression in defined reference area within the bulb. $N = 10$–12 gray anagen VI HFs from five different donors treated with Rapamycin 20 ng/ml or untreated (vehicle) for 7 days.

L Representative images of α-MSH immunofluorescence.

Data information: Only anagen VI HFs were investigated and analyses performed in defined reference areas (dotted areas) in the HFPU. Mean ± SEM, Mann–Whitney U-test (A) or Student's *t*-test (C, E, G, I, K), $^*P < 0.05$. Scale bar: 50 μm. Samples from each donor represented by a different color. Nuclei stained with DAPI.

© 2023 The Authors

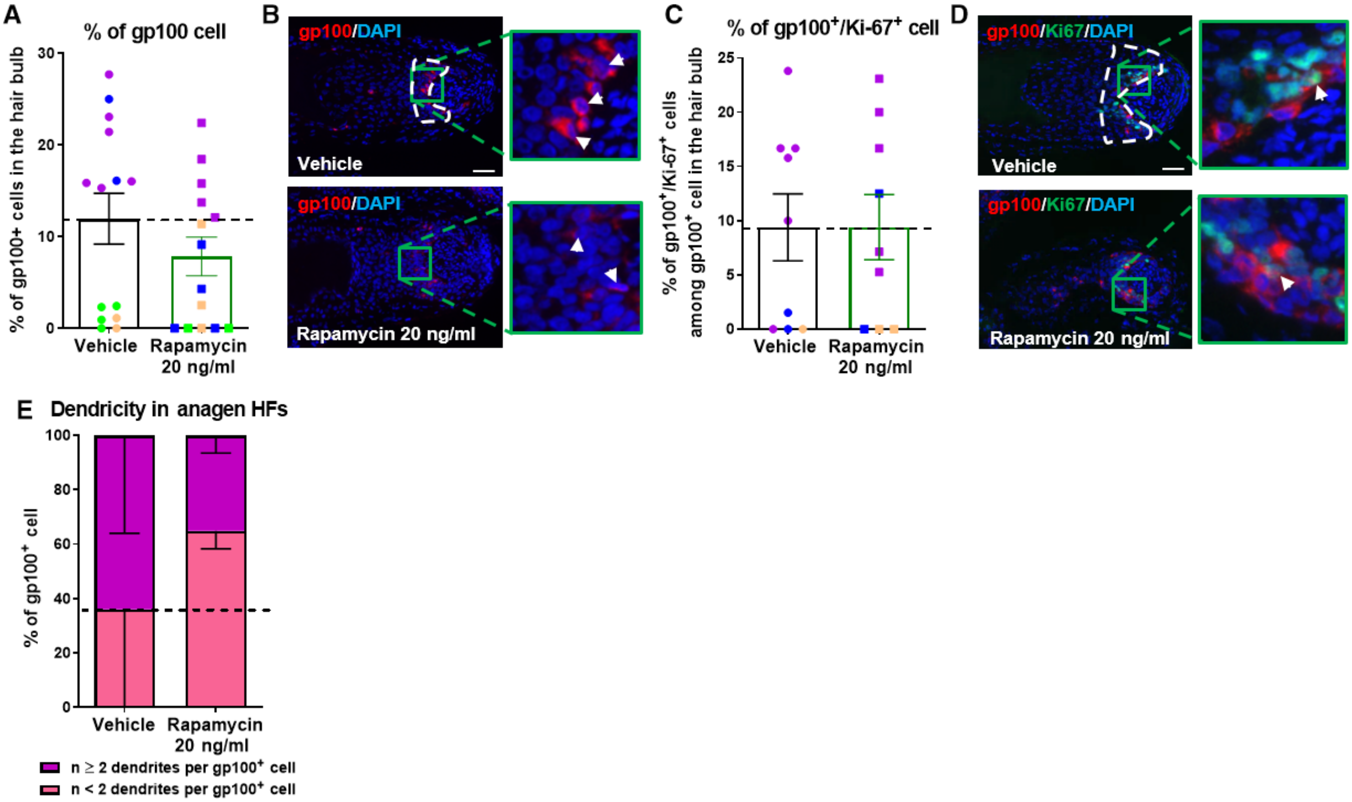

Figure EV4. mTORC1 inhibition does not significantly alter melanocyte number, proliferation state, and dendricity in gray/white human scalp gray hair follicles.

A Quantitative immunohistomorphometry of the number of gp100^{+} cells. *N* = 14 gray anagen VI HFs from four different donors treated with Rapamycin 20 ng/ml or untreated (vehicle) for 7 days.

B Representative images of gp100 immunofluorescence. Arrows indicate gp100^{+} cells.

C Quantitative analysis of gp100^{+}/ki-67^{+} cell number. *N* = 9 gray anagen VI HFs from three different donors treated with Rapamycin 20 ng/ml or untreated (vehicle) for 7 days.

D Representative images of gp100/ki-67 immunofluorescence. Arrows indicate gp100^{+}Ki-67^{+} cells.

E Quantitative analysis of melanocyte dendricity. *N* = 10–12 gray anagen VI HFs from four different donors treated with Rapamycin 20 ng/ml or untreated (vehicle) for 7 days.

Data information: Only anagen VI HFs were investigated and analyses performed in defined reference areas (dotted areas) in the HFPU. Mean ± SEM, Mann–Whitney U-test. Scale bar: 50 μm. Samples from each donor represented by a different color. Nuclei stained with DAPI.

 © 2023 The Authors
