# Supplementary figures and images for: mTORC1 activity negatively regulates human hair follicle growth and pigmentation
Source: EMBO Rep. 2023 May 22;24(7):e56574. doi: 10.15252/embr.202256574 (PMC10328083; doi:10.15252/embr.202256574)

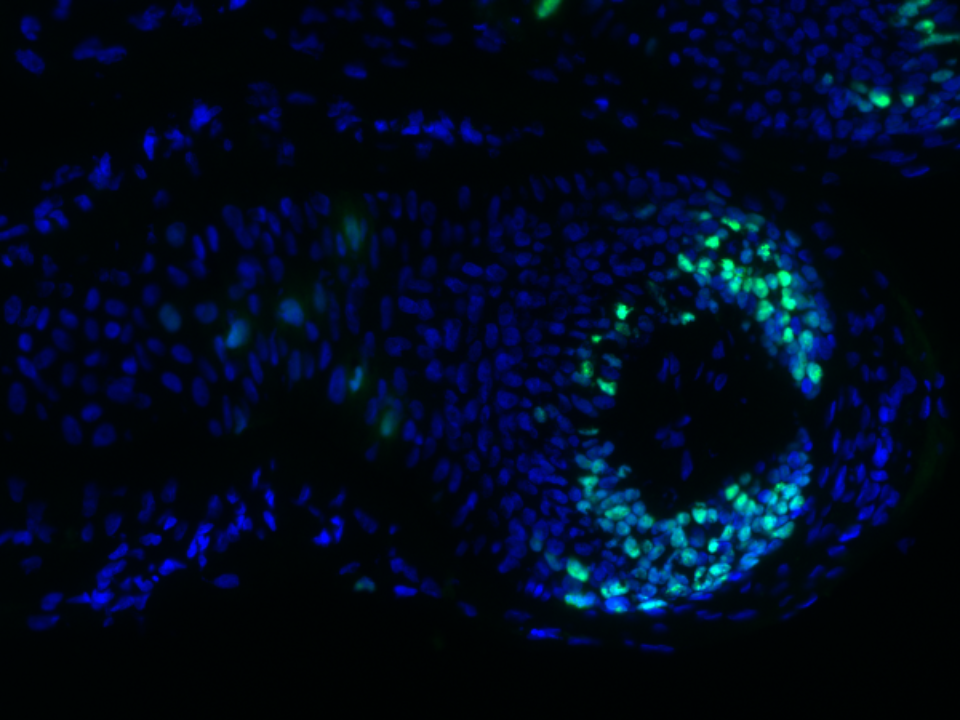

Supplement: Supplementary file 2 — Source Data for Expanded View [file EMBR-24-e56574-s004.zip › Source data/Fig EV1/EV1B/EV1B Ki67 Rapa_Overlay.tif]

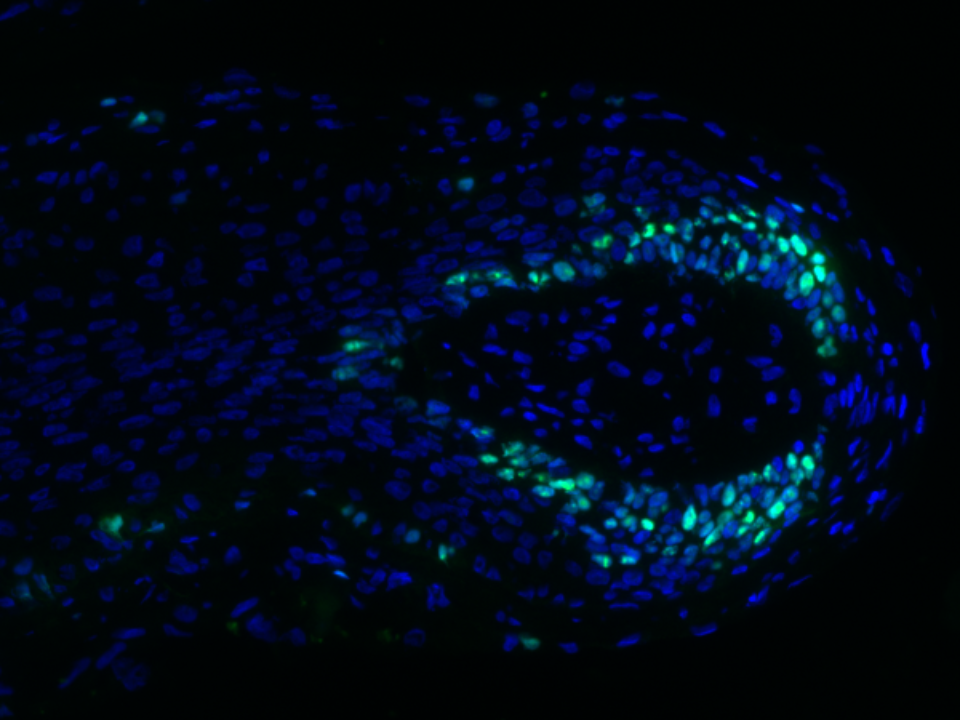

Supplement: Supplementary file 2 — Source Data for Expanded View [file EMBR-24-e56574-s004.zip › Source data/Fig EV1/EV1B/EV1B Ki67 Vehicle_Overlay.tif]

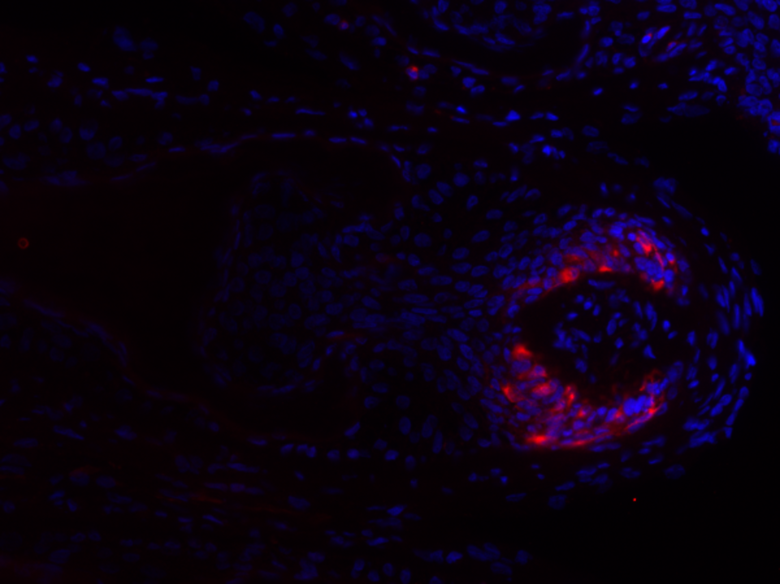

Supplement: Supplementary file 2 — Source Data for Expanded View [file EMBR-24-e56574-s004.zip › Source data/Fig EV1/EV1D/EV1D gp100 Rapa_Overlay.tif]

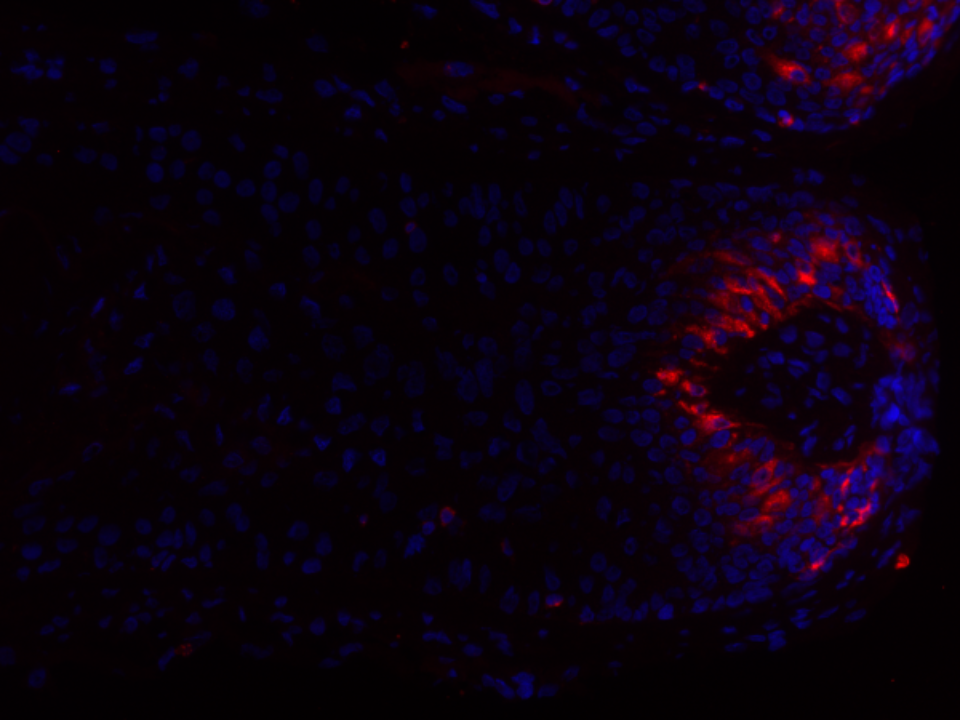

Supplement: Supplementary file 2 — Source Data for Expanded View [file EMBR-24-e56574-s004.zip › Source data/Fig EV1/EV1D/EV1D gp100 Vehicle_Overlay.tif]

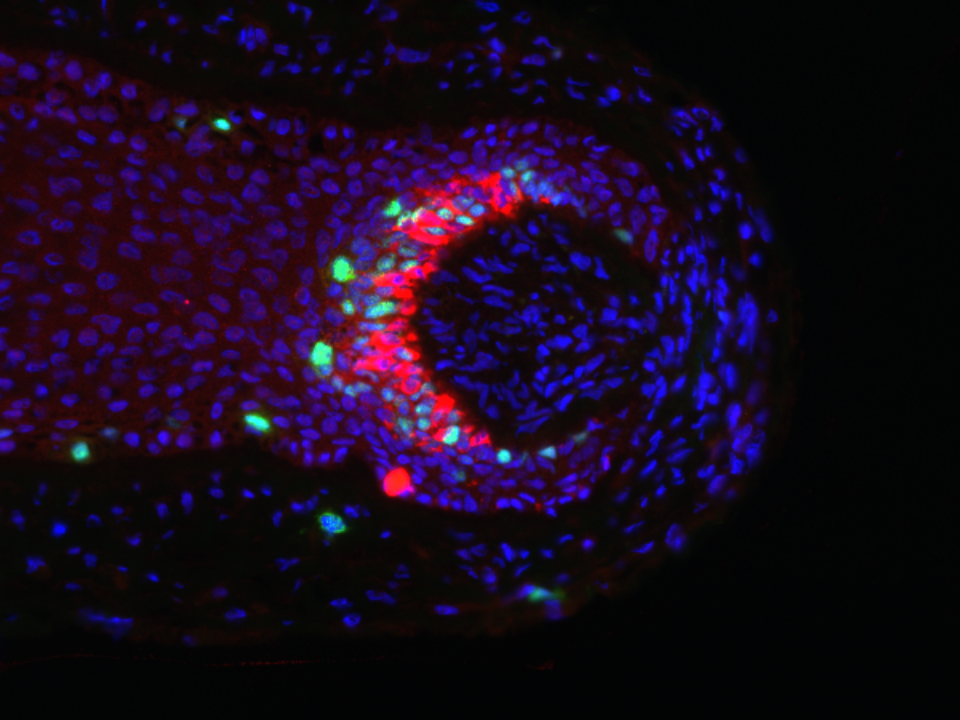

Supplement: Supplementary file 2 — Source Data for Expanded View [file EMBR-24-e56574-s004.zip › Source data/Fig EV1/EV1F/EV1F gp100Ki67 Rapa_Overlay.tif]

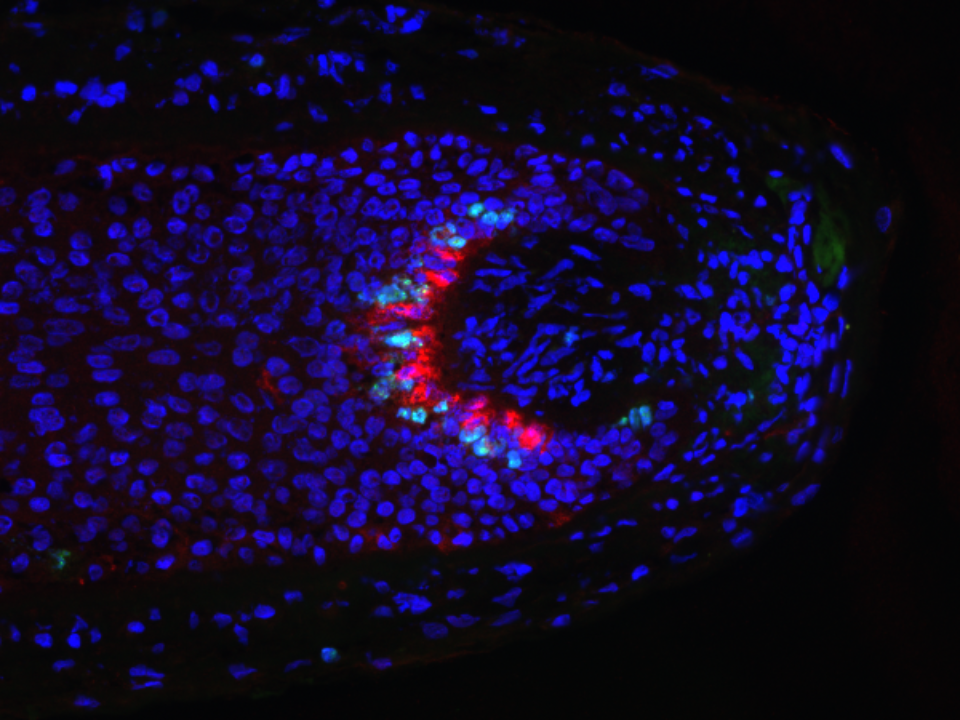

Supplement: Supplementary file 2 — Source Data for Expanded View [file EMBR-24-e56574-s004.zip › Source data/Fig EV1/EV1F/EV1F gp100Ki67 Vehicle_Overlay.tif]

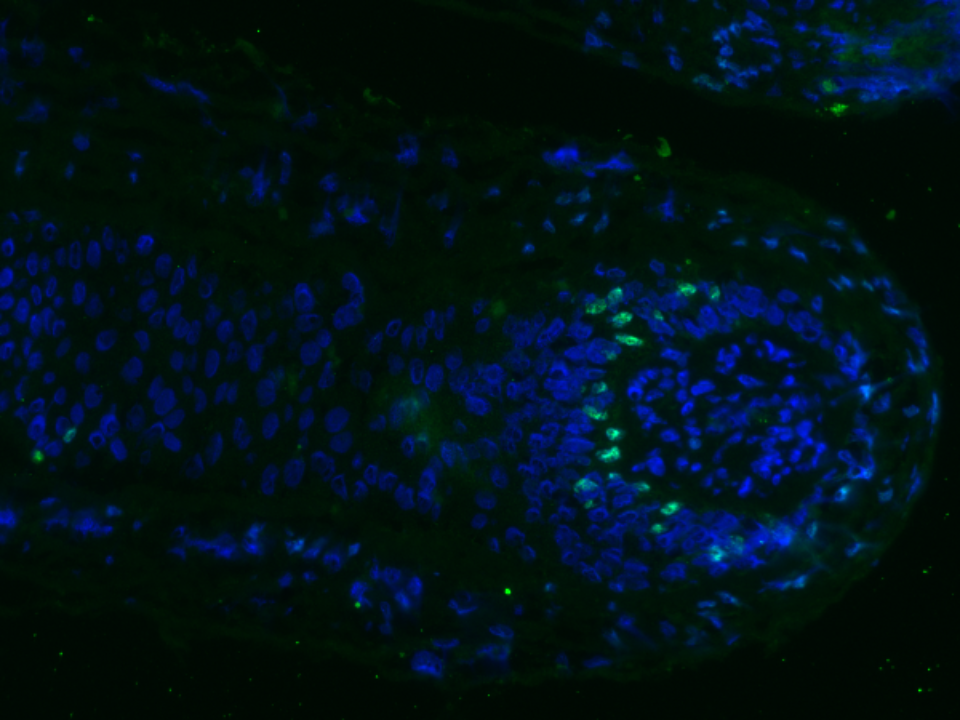

Supplement: Supplementary file 2 — Source Data for Expanded View [file EMBR-24-e56574-s004.zip › Source data/Fig EV1/EV1H/EV1H MITF Rapa_Overlay.tif]

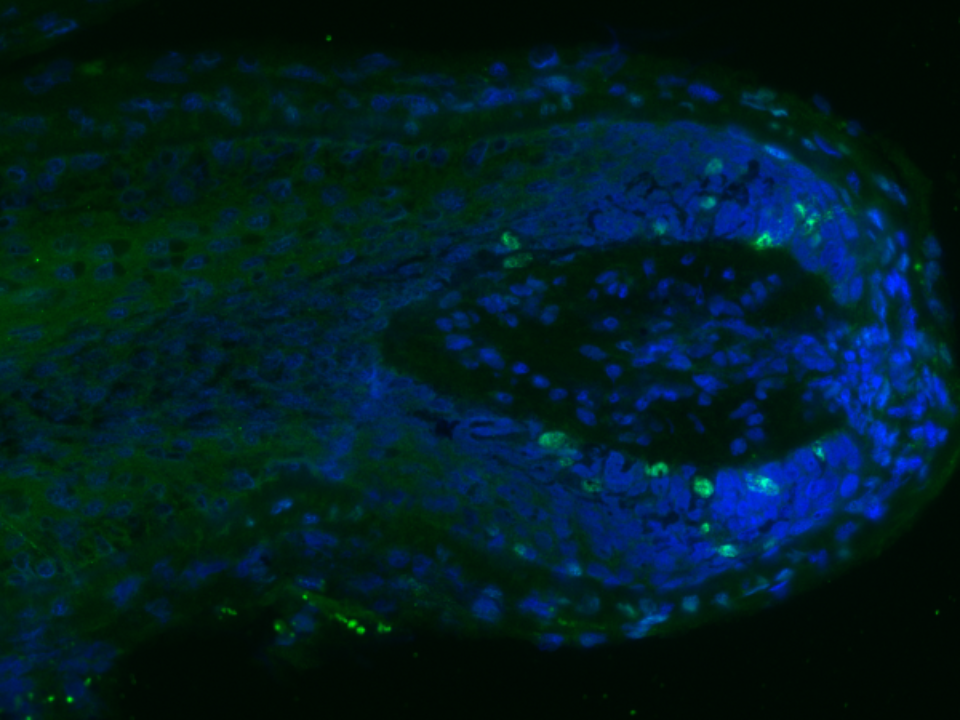

Supplement: Supplementary file 2 — Source Data for Expanded View [file EMBR-24-e56574-s004.zip › Source data/Fig EV1/EV1H/EV1H MITF Vehicle_Overlay.tif]

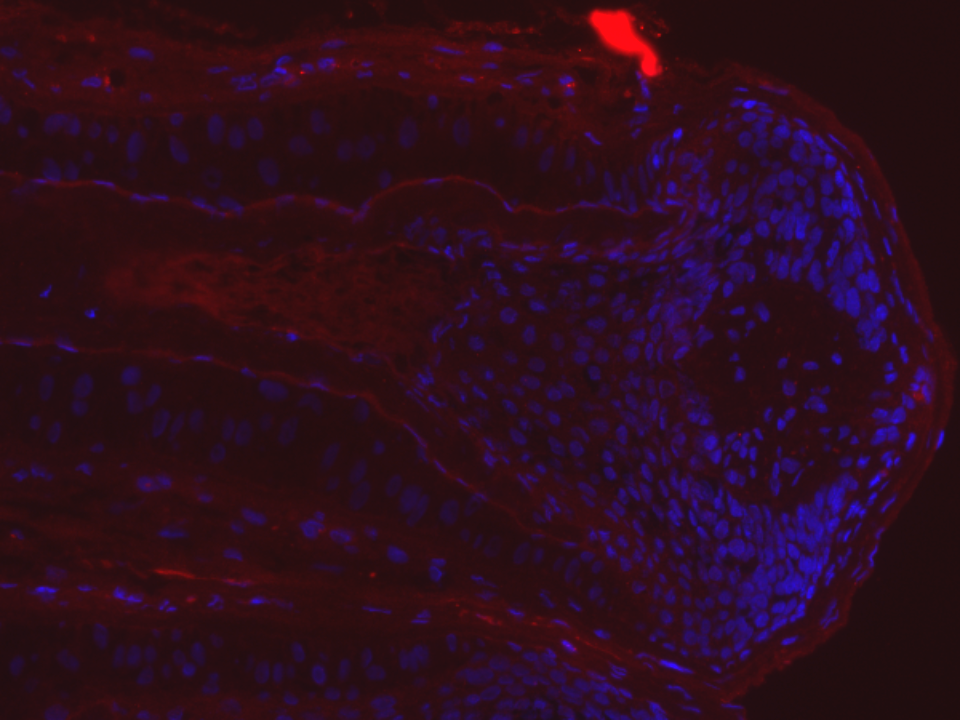

Supplement: Supplementary file 2 — Source Data for Expanded View [file EMBR-24-e56574-s004.zip › Source data/Fig EV1/EV1J/EV1J pMITF Rapa_Overlay.tif]

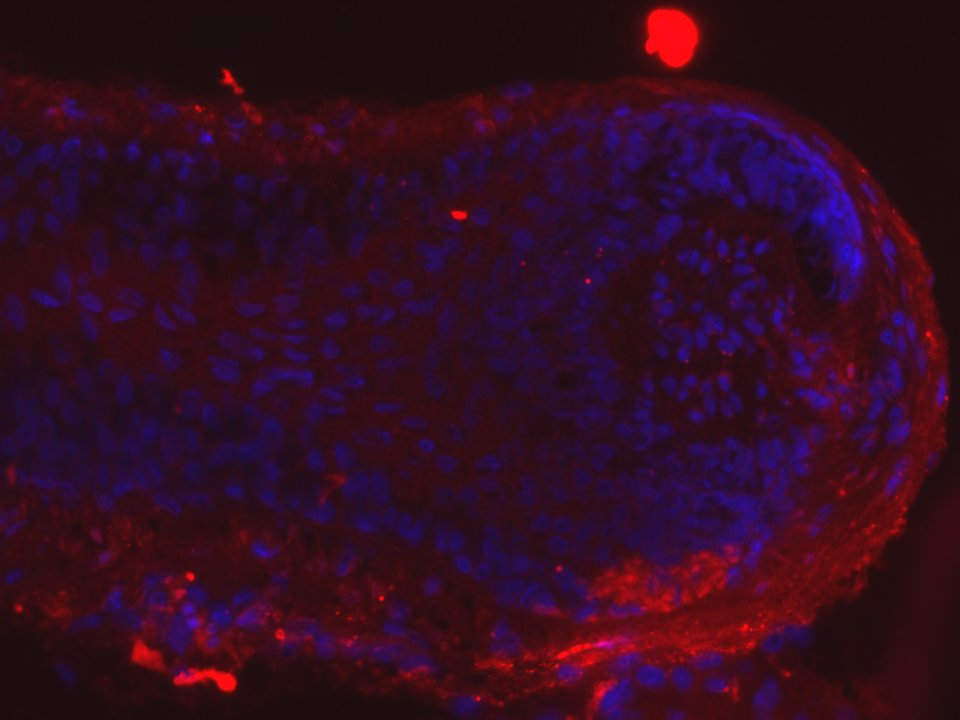

Supplement: Supplementary file 2 — Source Data for Expanded View [file EMBR-24-e56574-s004.zip › Source data/Fig EV1/EV1J/EV1J pMITF Vehicle_Overlay.tif]

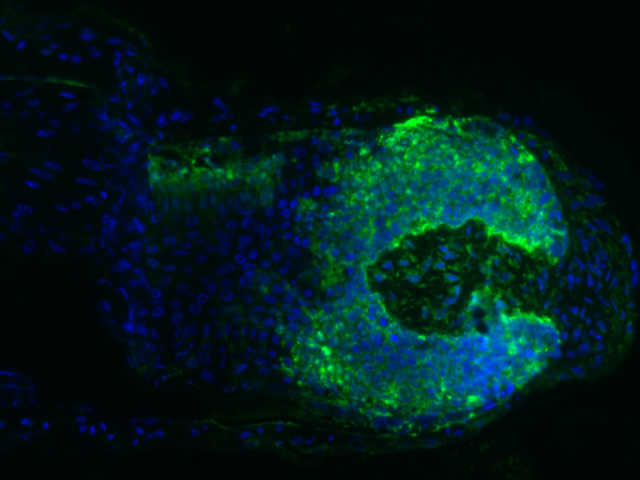

Supplement: Supplementary file 4 — Source Data for Figure 1 [file EMBR-24-e56574-s008.zip › 1B/1B Grey HF pS6_Overlay.tif]

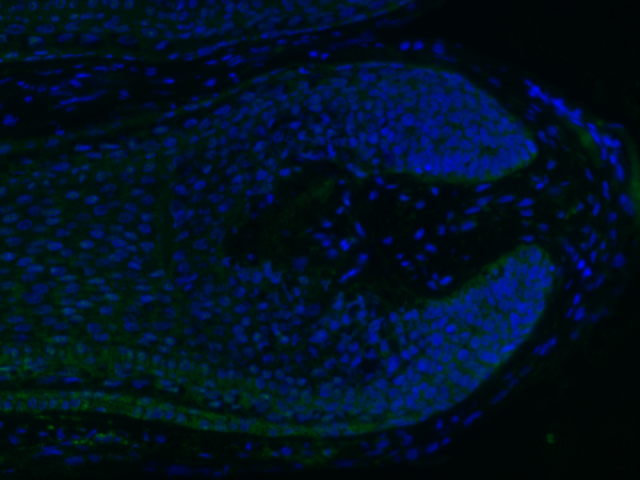

Supplement: Supplementary file 4 — Source Data for Figure 1 [file EMBR-24-e56574-s008.zip › 1B/1B Pigmented HF pS6_Overlay.tif]

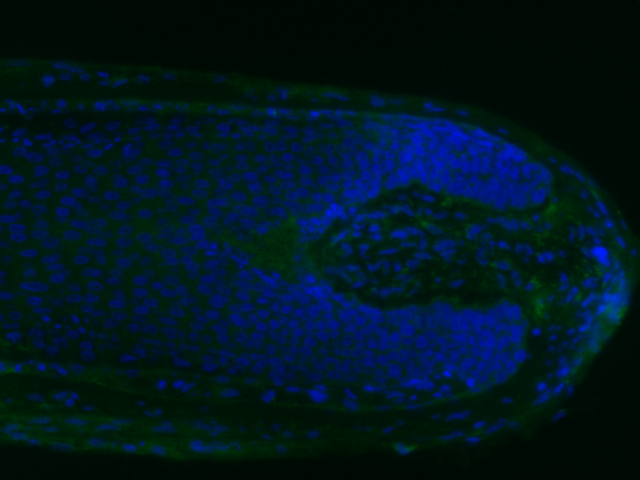

Supplement: Supplementary file 4 — Source Data for Figure 1 [file EMBR-24-e56574-s008.zip › 1D/1D Grey HF TSC2_Overlay.tif]

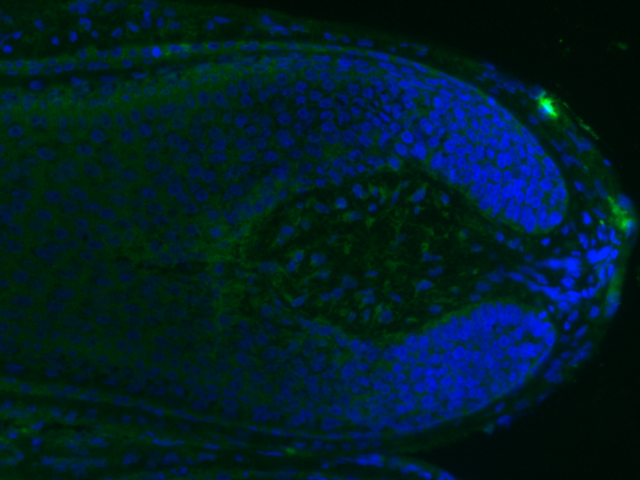

Supplement: Supplementary file 4 — Source Data for Figure 1 [file EMBR-24-e56574-s008.zip › 1D/1D Pigmented HF TSC2_Overlay.tif]

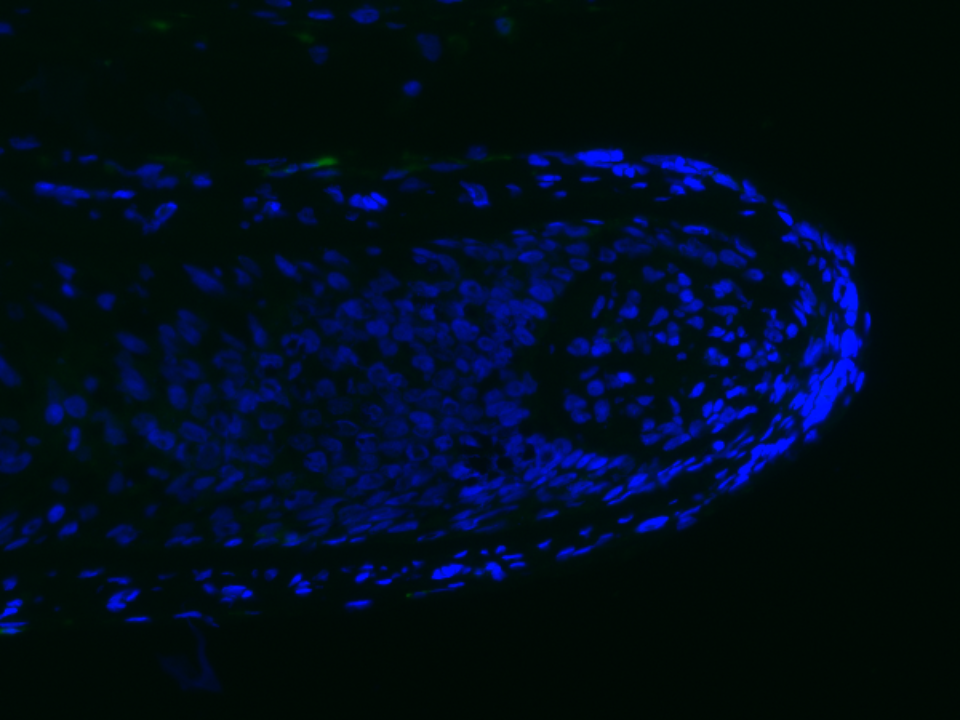

Supplement: Supplementary file 5 — Source Data for Figure 2 [file EMBR-24-e56574-s005.zip › 2B/2B Rapa pS6_Overlay.tif]

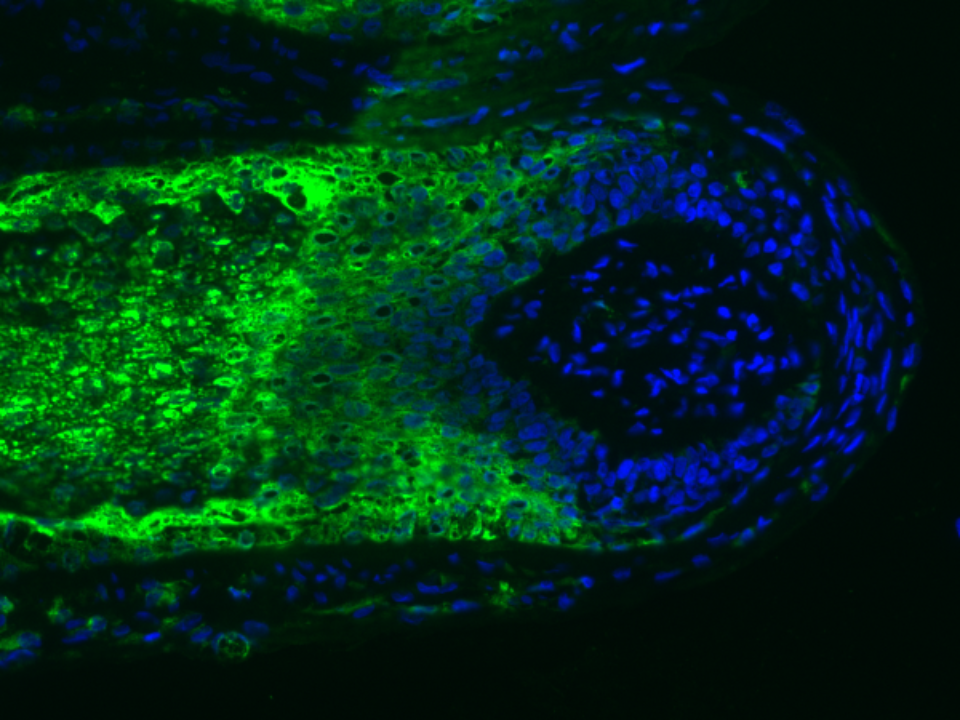

Supplement: Supplementary file 5 — Source Data for Figure 2 [file EMBR-24-e56574-s005.zip › 2B/2B Vehicle pS6_Overlay.tif]

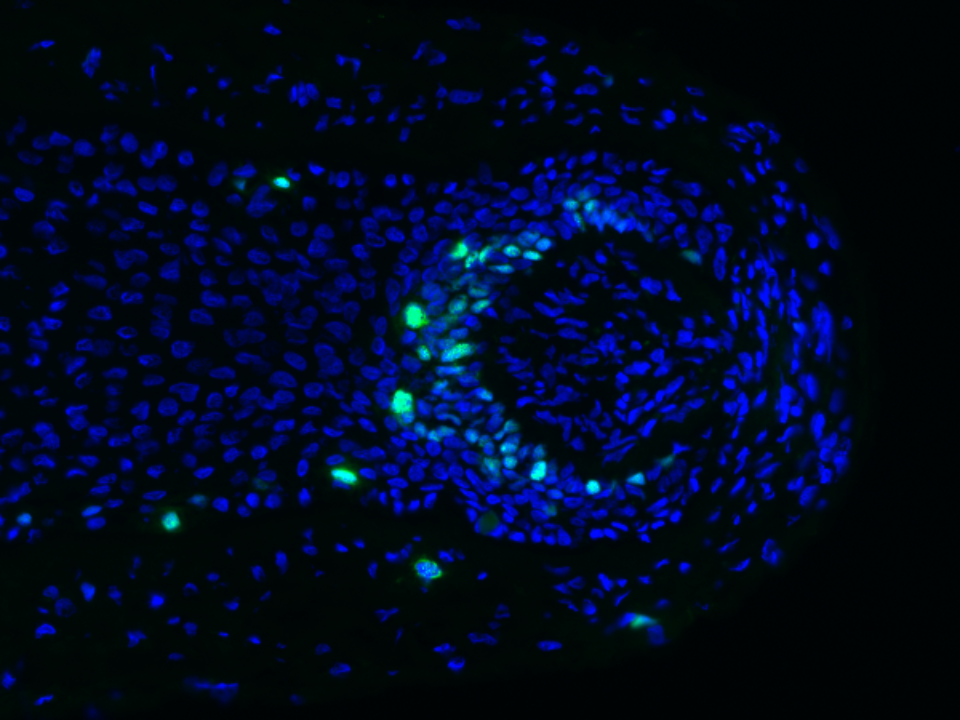

Supplement: Supplementary file 5 — Source Data for Figure 2 [file EMBR-24-e56574-s005.zip › 2D/2D Rapa Ki67 Anagen_Overlay.tif]

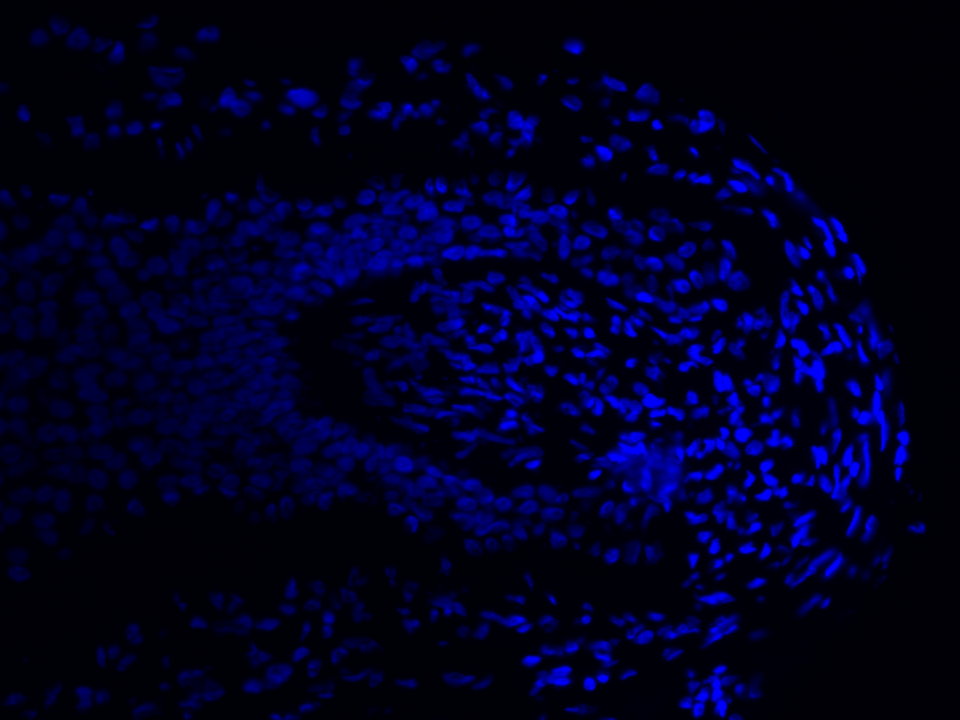

Supplement: Supplementary file 5 — Source Data for Figure 2 [file EMBR-24-e56574-s005.zip › 2D/2D Rapa Ki67 Catagen_Overlay.tif]

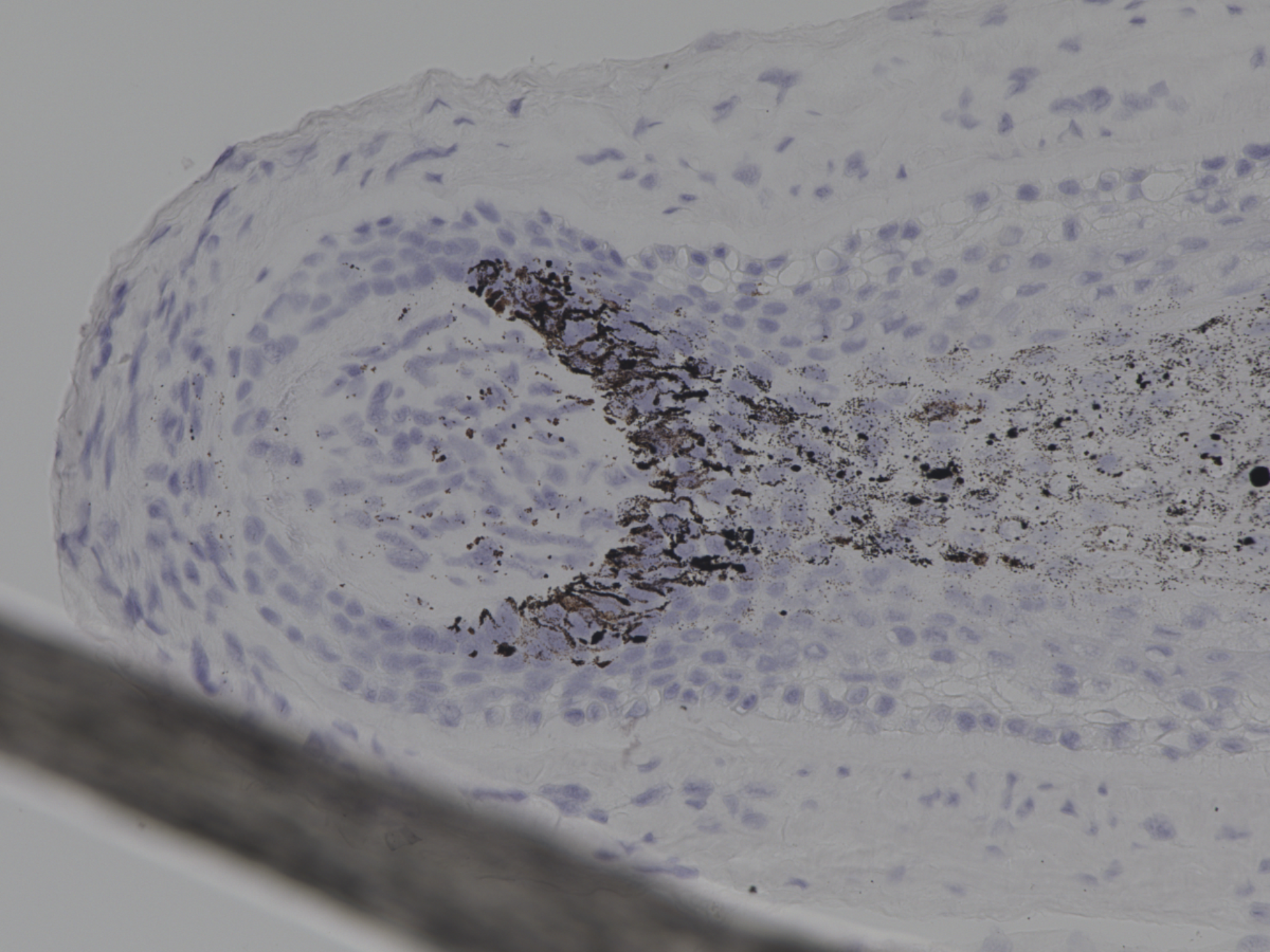

Supplement: Supplementary file 5 — Source Data for Figure 2 [file EMBR-24-e56574-s005.zip › 2D/2D Rapa Masson-Fontana Anagen.tif]

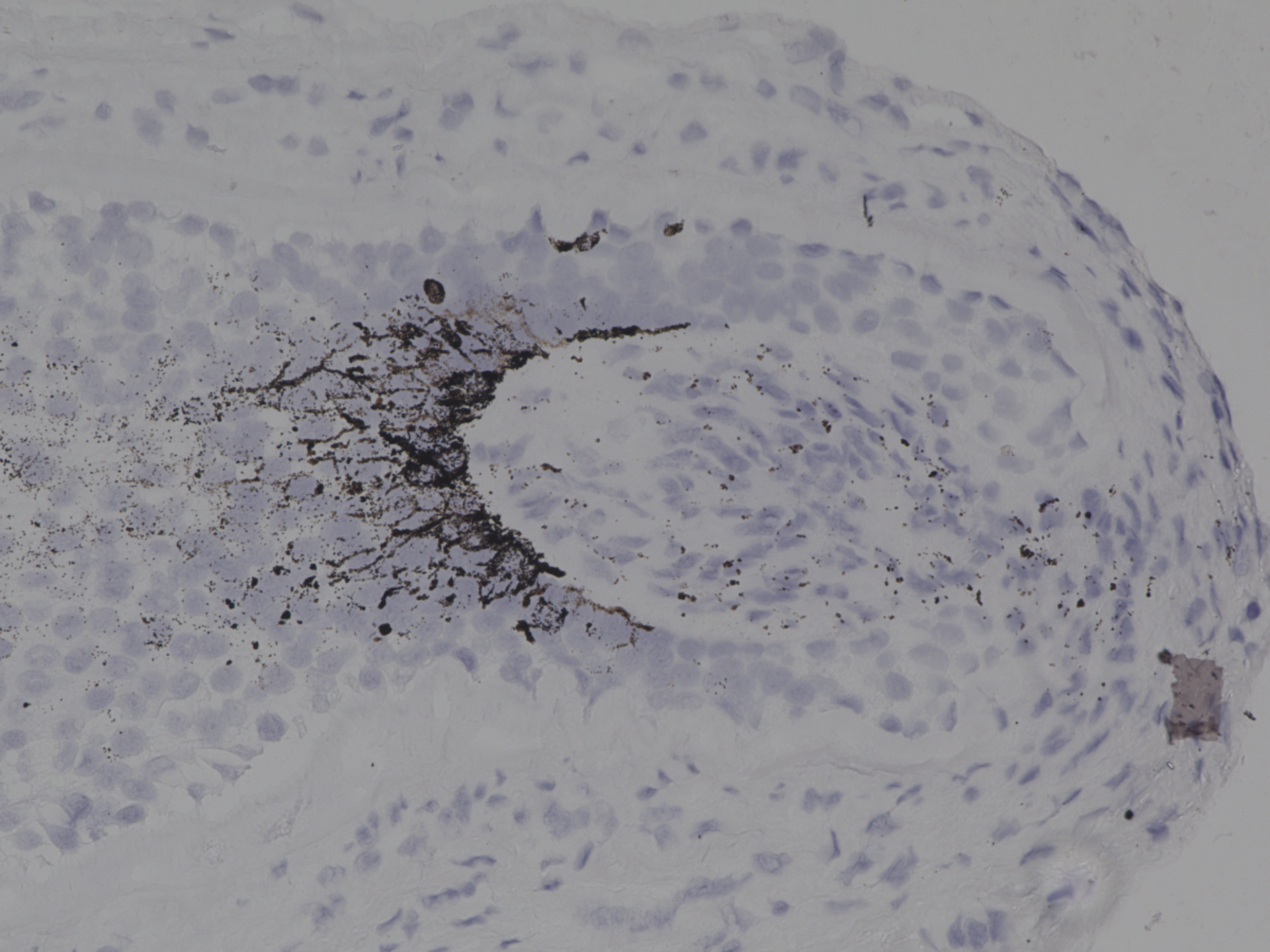

Supplement: Supplementary file 5 — Source Data for Figure 2 [file EMBR-24-e56574-s005.zip › 2D/2D Rapa Masson-Fontana Catagen.tif]

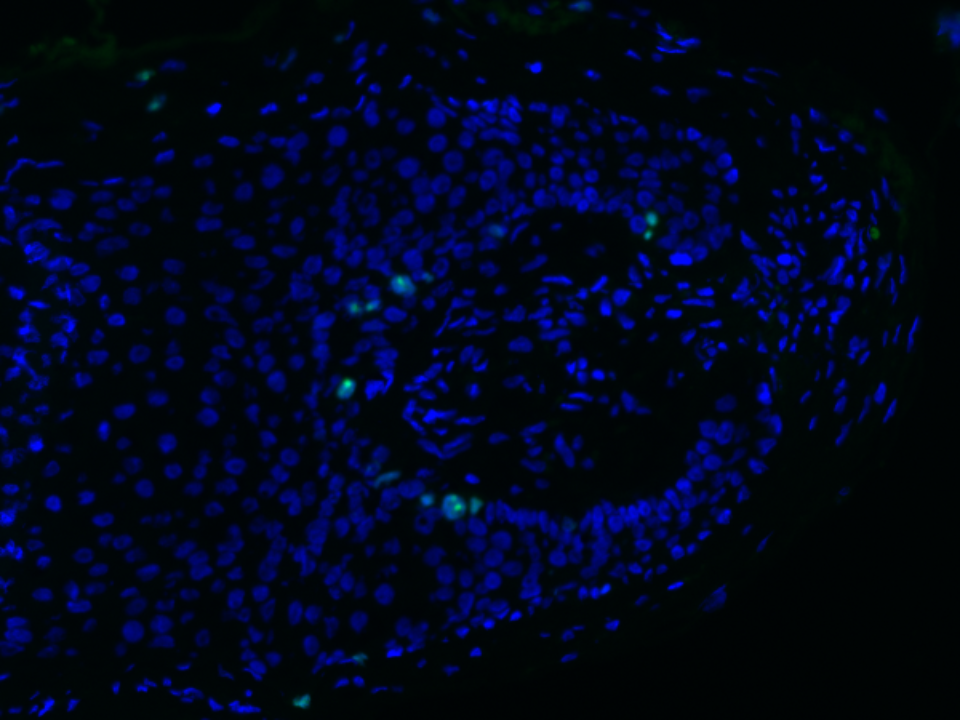

Supplement: Supplementary file 5 — Source Data for Figure 2 [file EMBR-24-e56574-s005.zip › 2D/2D Vehicle Ki67 Anagen_Overlay.tif]

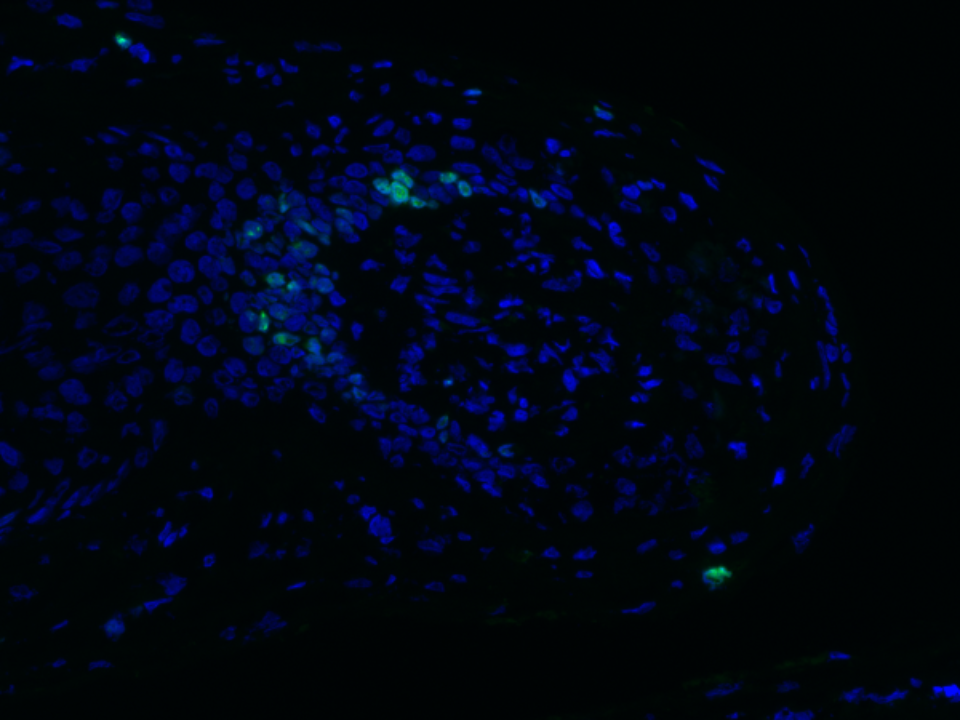

Supplement: Supplementary file 5 — Source Data for Figure 2 [file EMBR-24-e56574-s005.zip › 2D/2D Vehicle Ki67 Catagen_Overlay.tif]

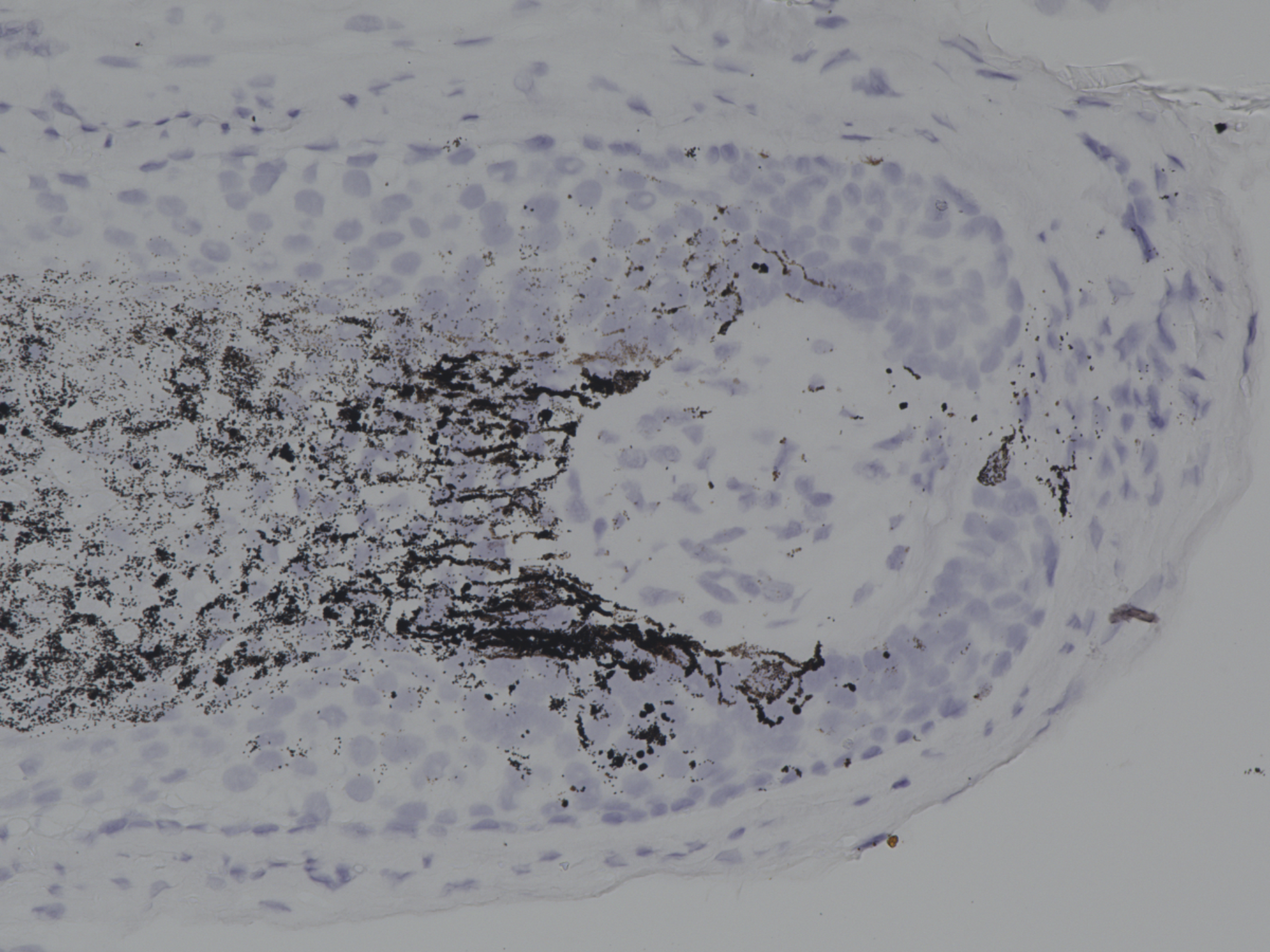

Supplement: Supplementary file 5 — Source Data for Figure 2 [file EMBR-24-e56574-s005.zip › 2D/2D Vehicle Masson-Fontana Anagen.tif]

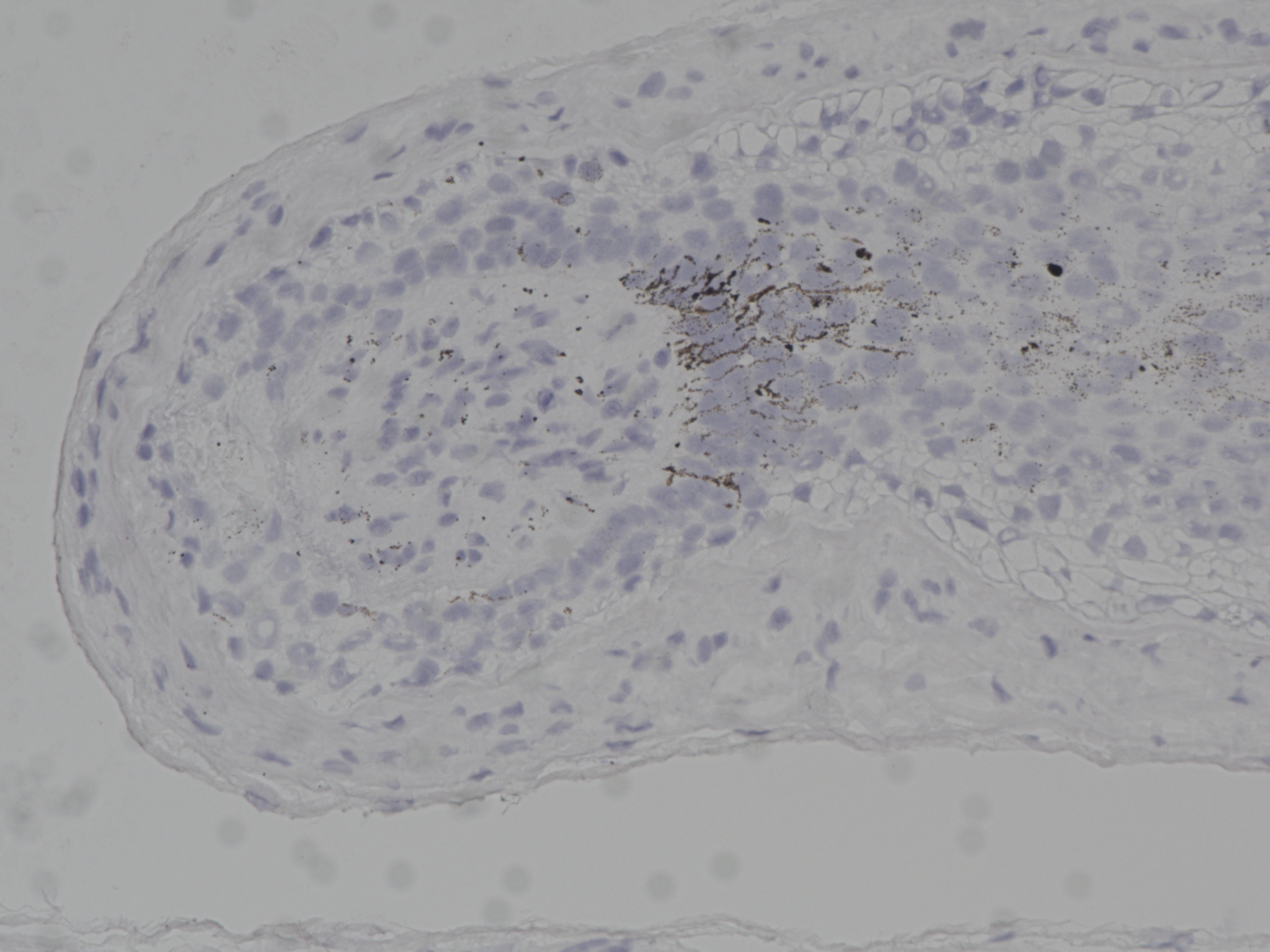

Supplement: Supplementary file 5 — Source Data for Figure 2 [file EMBR-24-e56574-s005.zip › 2D/2D Vehicle Masson-Fontana Catagen.tif]

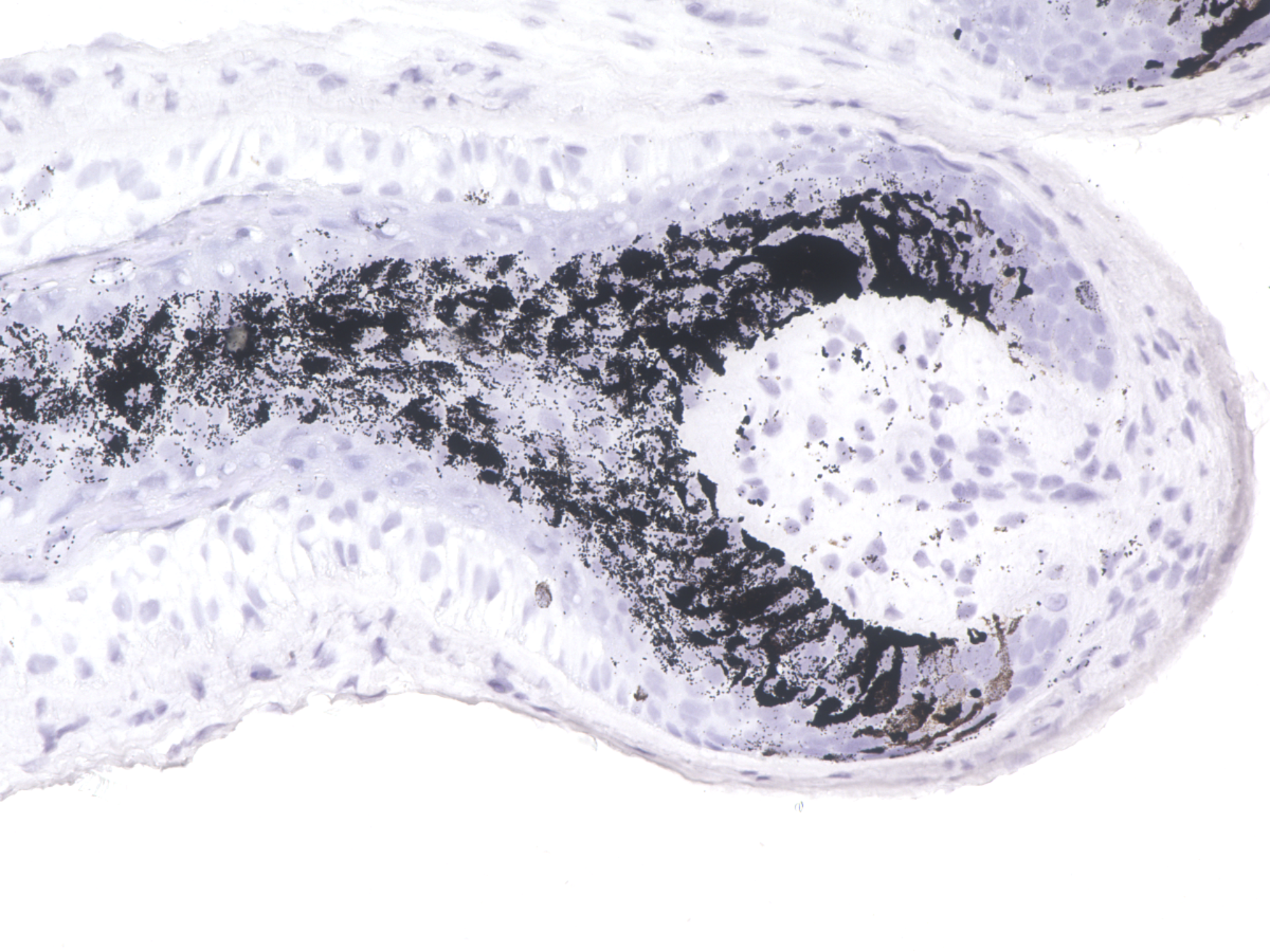

Supplement: Supplementary file 5 — Source Data for Figure 2 [file EMBR-24-e56574-s005.zip › 2F/2F Masson-Fontana Rapa.tif]

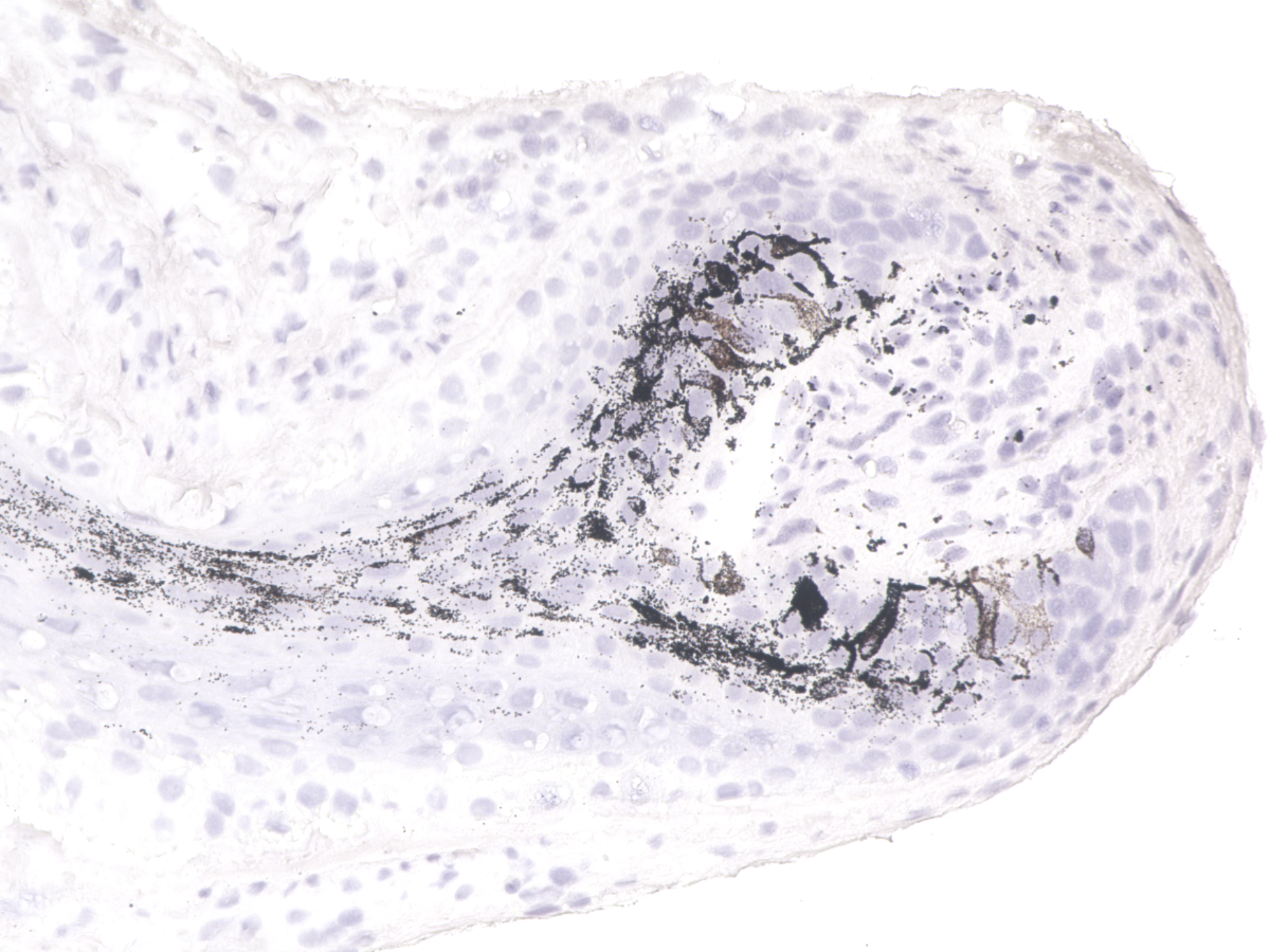

Supplement: Supplementary file 5 — Source Data for Figure 2 [file EMBR-24-e56574-s005.zip › 2F/2F Masson-Fontana Vehicle.tif]

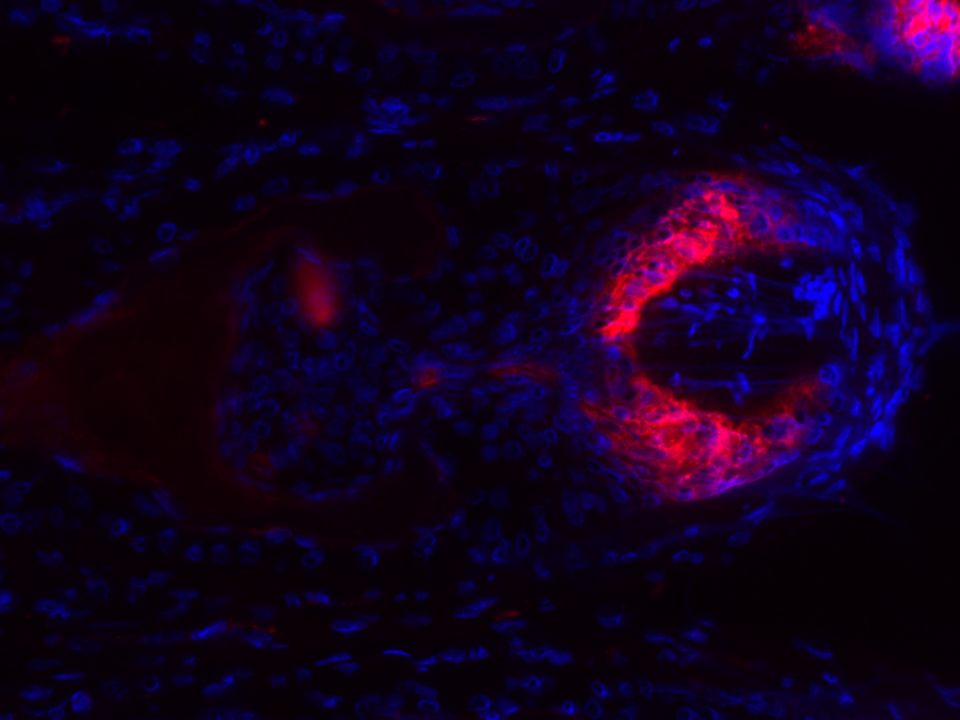

Supplement: Supplementary file 5 — Source Data for Figure 2 [file EMBR-24-e56574-s005.zip › 2H/2H Tyrosinase Rapa_Overlay.tif]

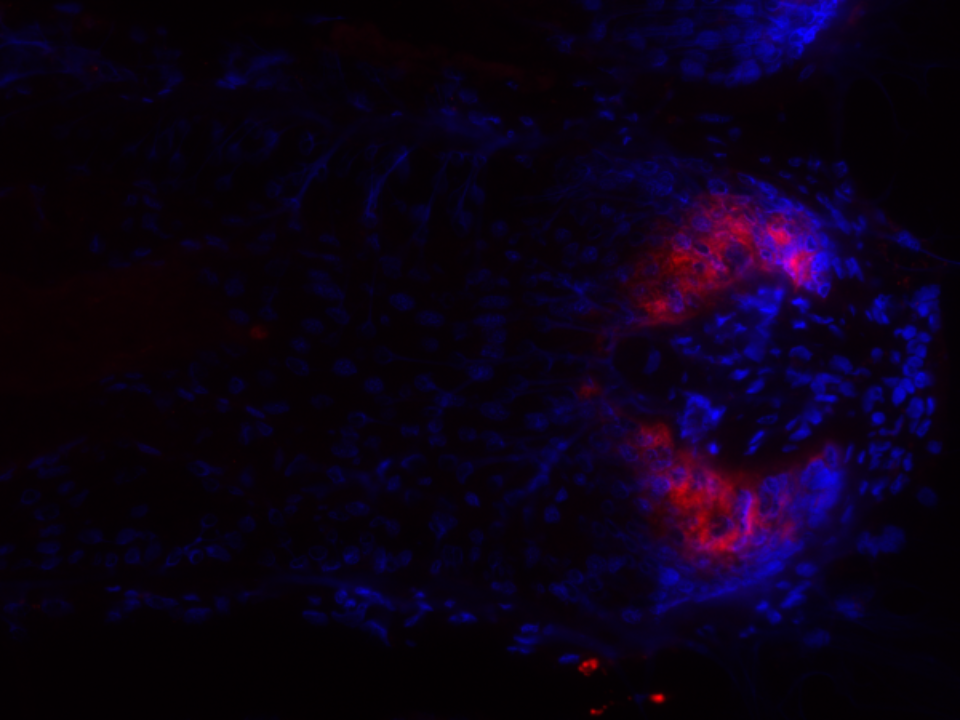

Supplement: Supplementary file 5 — Source Data for Figure 2 [file EMBR-24-e56574-s005.zip › 2H/2H Tyrosinase Vehicle_Overlay.tif]

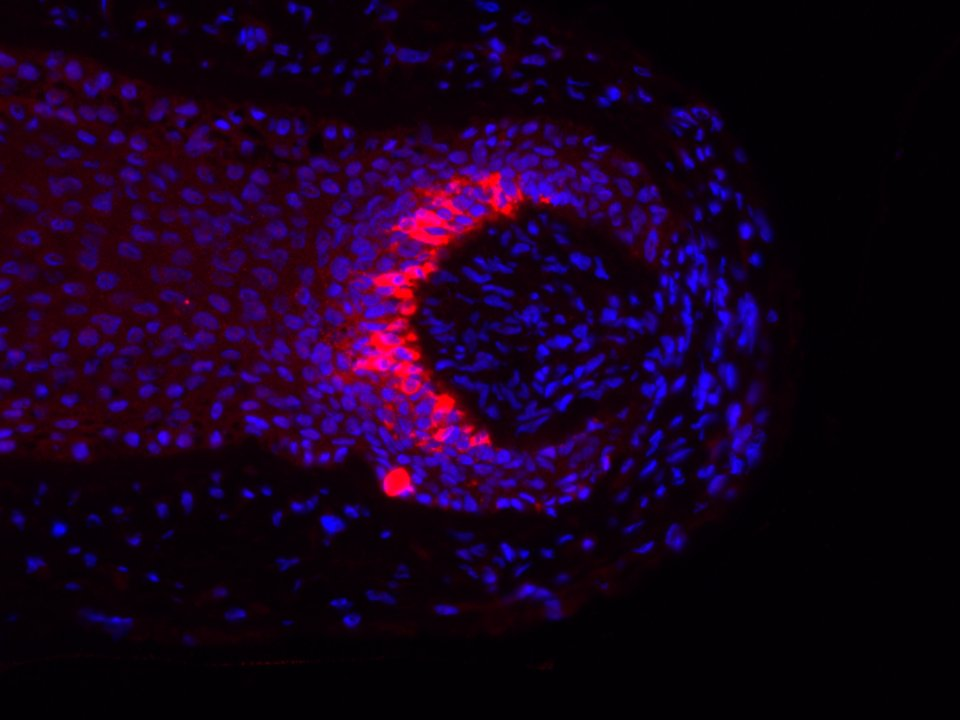

Supplement: Supplementary file 5 — Source Data for Figure 2 [file EMBR-24-e56574-s005.zip › 2J/2J Rapa gp100_Overlay.tif]

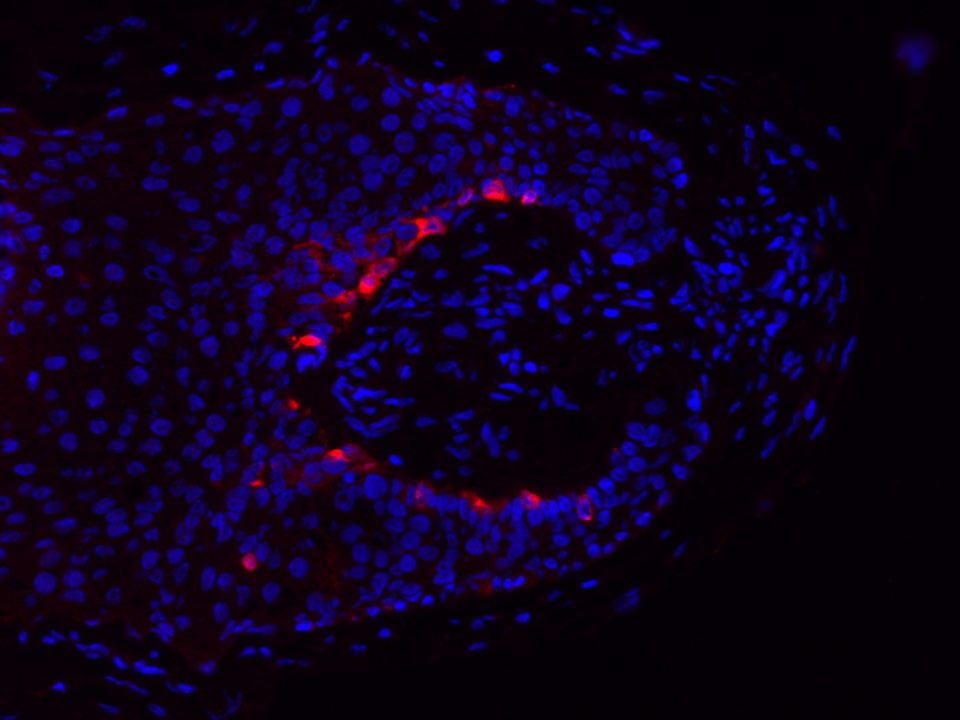

Supplement: Supplementary file 5 — Source Data for Figure 2 [file EMBR-24-e56574-s005.zip › 2J/2J Vehicle gp100_Overlay.tif]

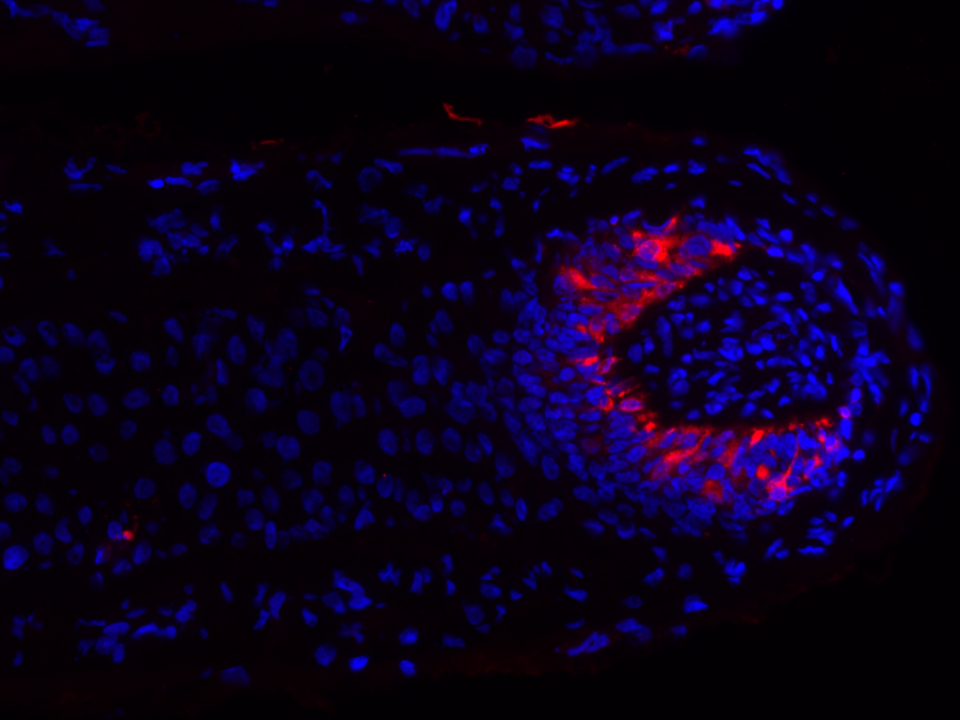

Supplement: Supplementary file 5 — Source Data for Figure 2 [file EMBR-24-e56574-s005.zip › 2L/2L gp100 Rapa_Overlay.tif]

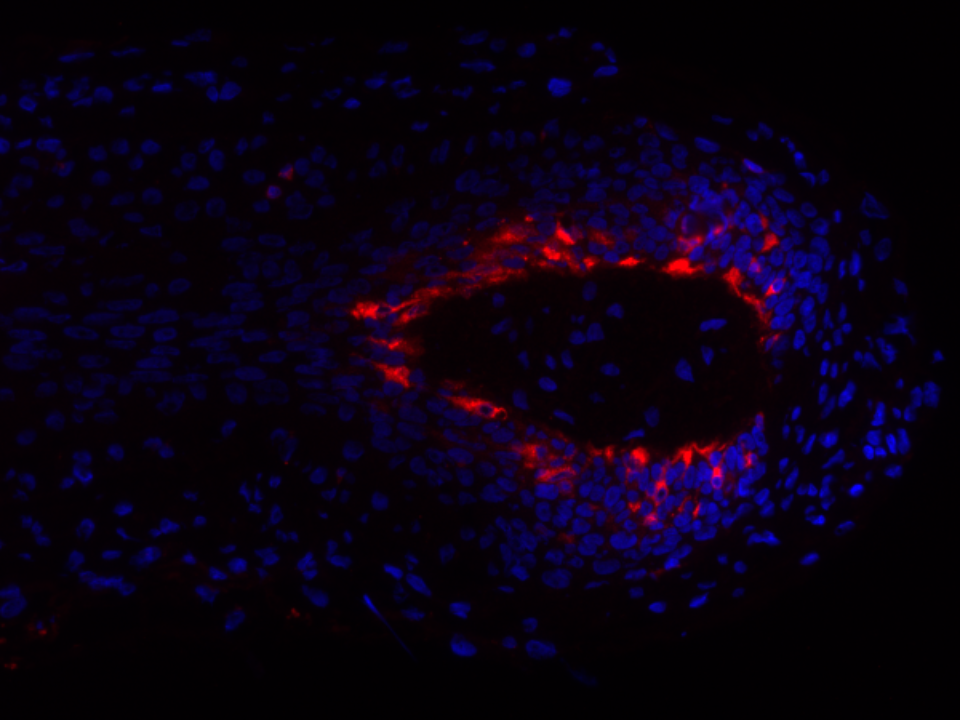

Supplement: Supplementary file 5 — Source Data for Figure 2 [file EMBR-24-e56574-s005.zip › 2L/2L gp100 Vehicle_Overlay.tif]

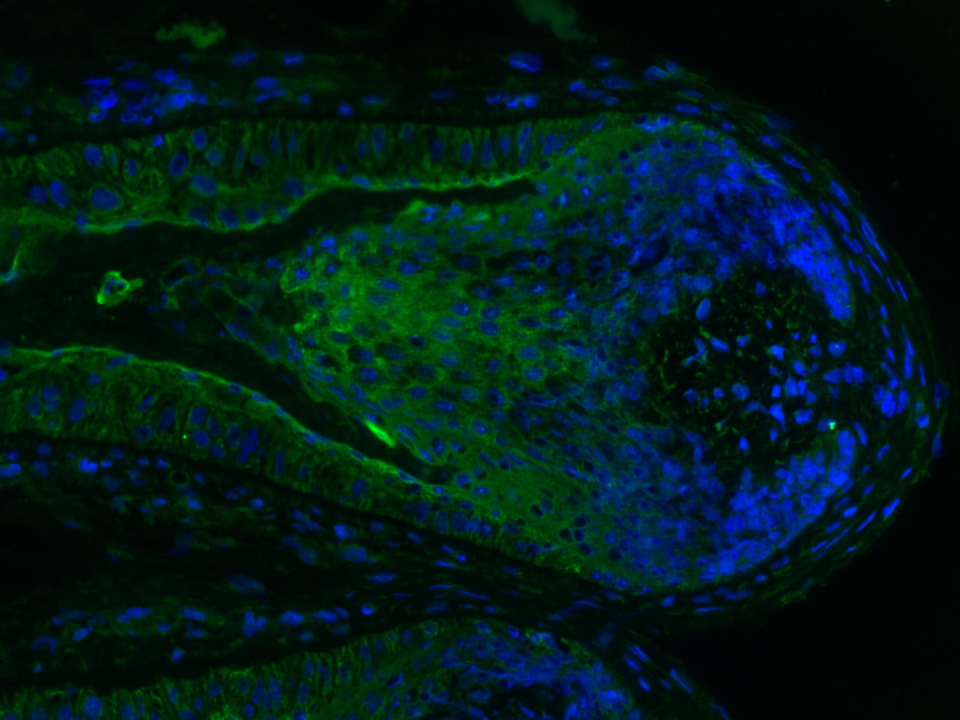

Supplement: Supplementary file 5 — Source Data for Figure 2 [file EMBR-24-e56574-s005.zip › 2N/2N aMSH Rapa_Overlay.tif]

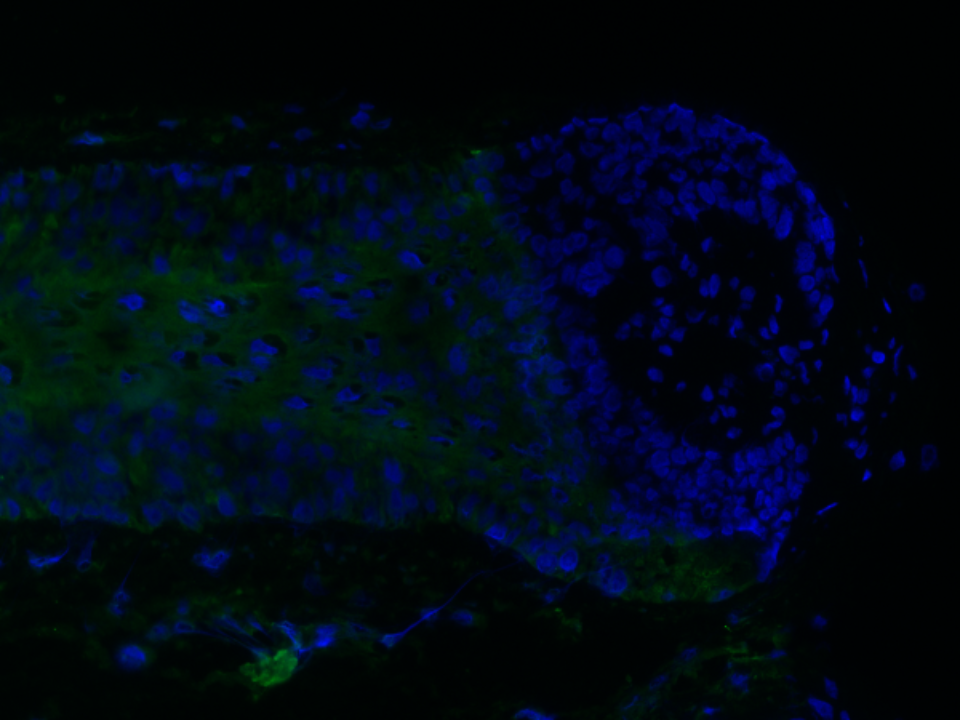

Supplement: Supplementary file 5 — Source Data for Figure 2 [file EMBR-24-e56574-s005.zip › 2N/2N aMSH Vehicle_Overlay.tif]

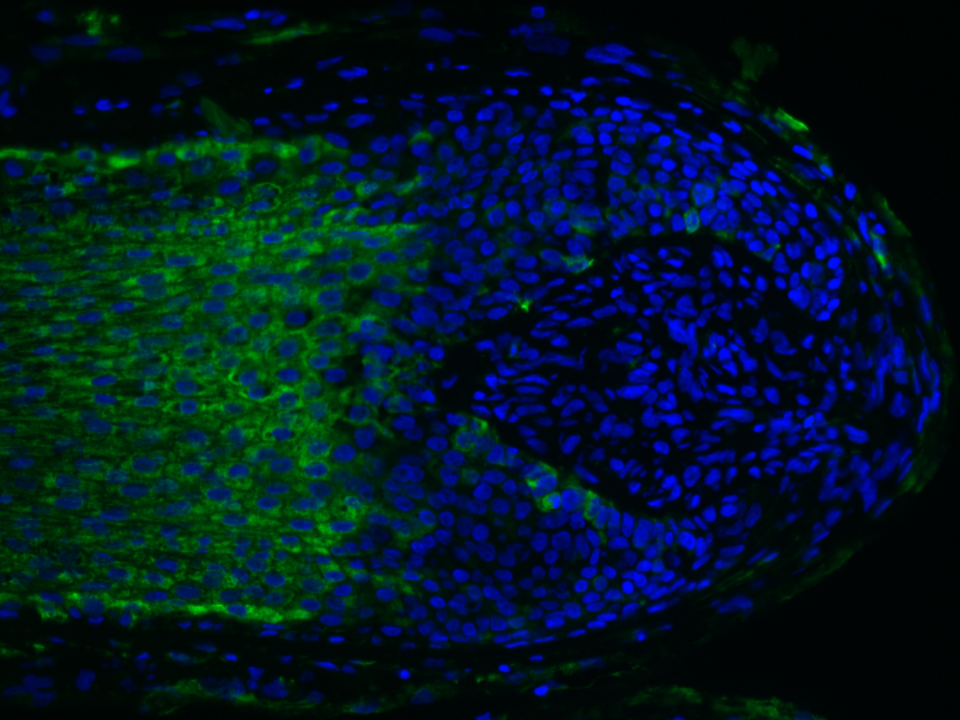

Supplement: Supplementary file 5 — Source Data for Figure 2 [file EMBR-24-e56574-s005.zip › 2P/2P p-S6 Agouti_Overlay.tif]

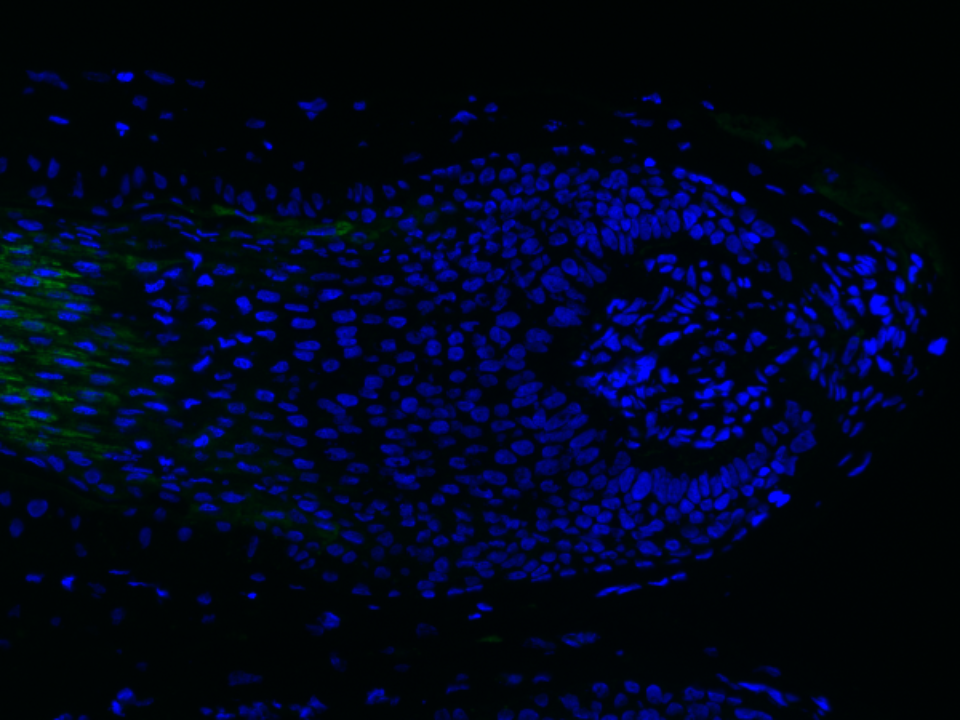

Supplement: Supplementary file 5 — Source Data for Figure 2 [file EMBR-24-e56574-s005.zip › 2P/2P p-S6 Rapa+Agouti_Overlay.tif]

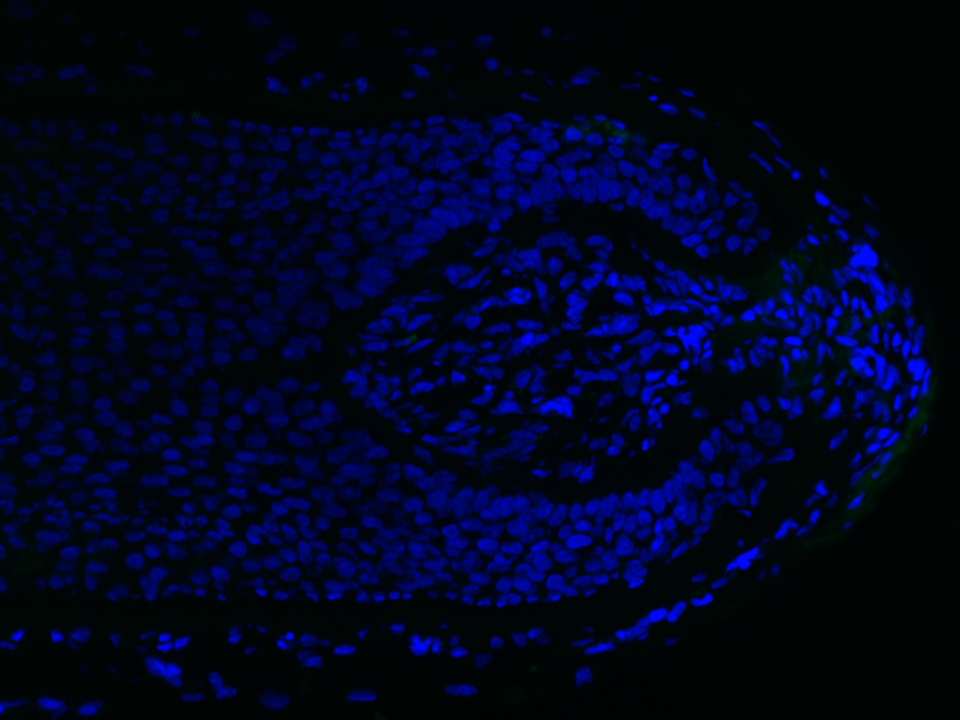

Supplement: Supplementary file 5 — Source Data for Figure 2 [file EMBR-24-e56574-s005.zip › 2P/2P p-S6 Rapa_Overlay.tif]

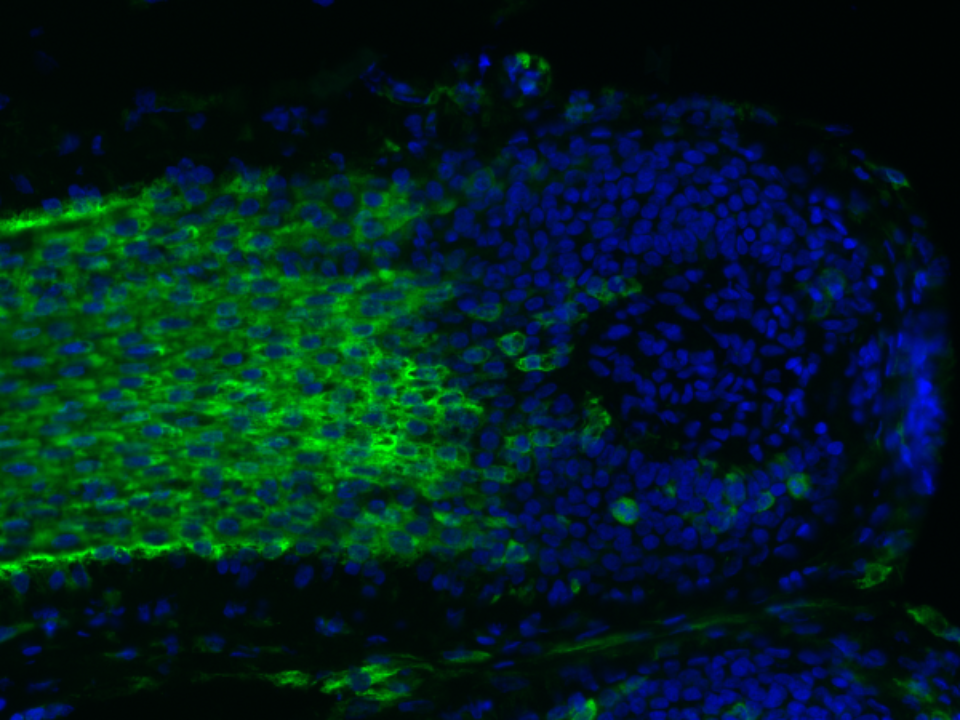

Supplement: Supplementary file 5 — Source Data for Figure 2 [file EMBR-24-e56574-s005.zip › 2P/2P p-S6 Vehicle_Overlay.tif]

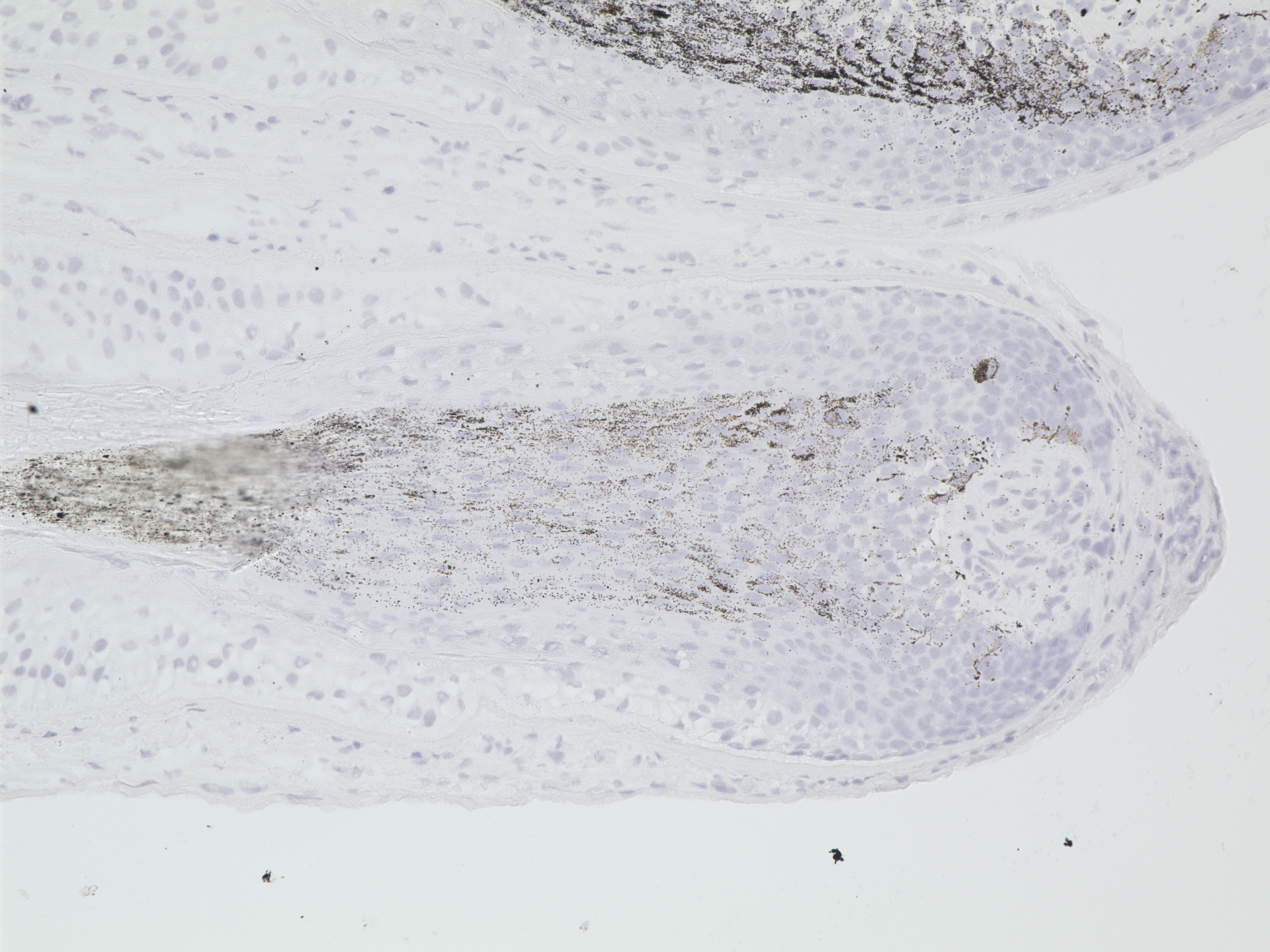

Supplement: Supplementary file 5 — Source Data for Figure 2 [file EMBR-24-e56574-s005.zip › 2R/2R Masson-Fontana Agouti.tif]

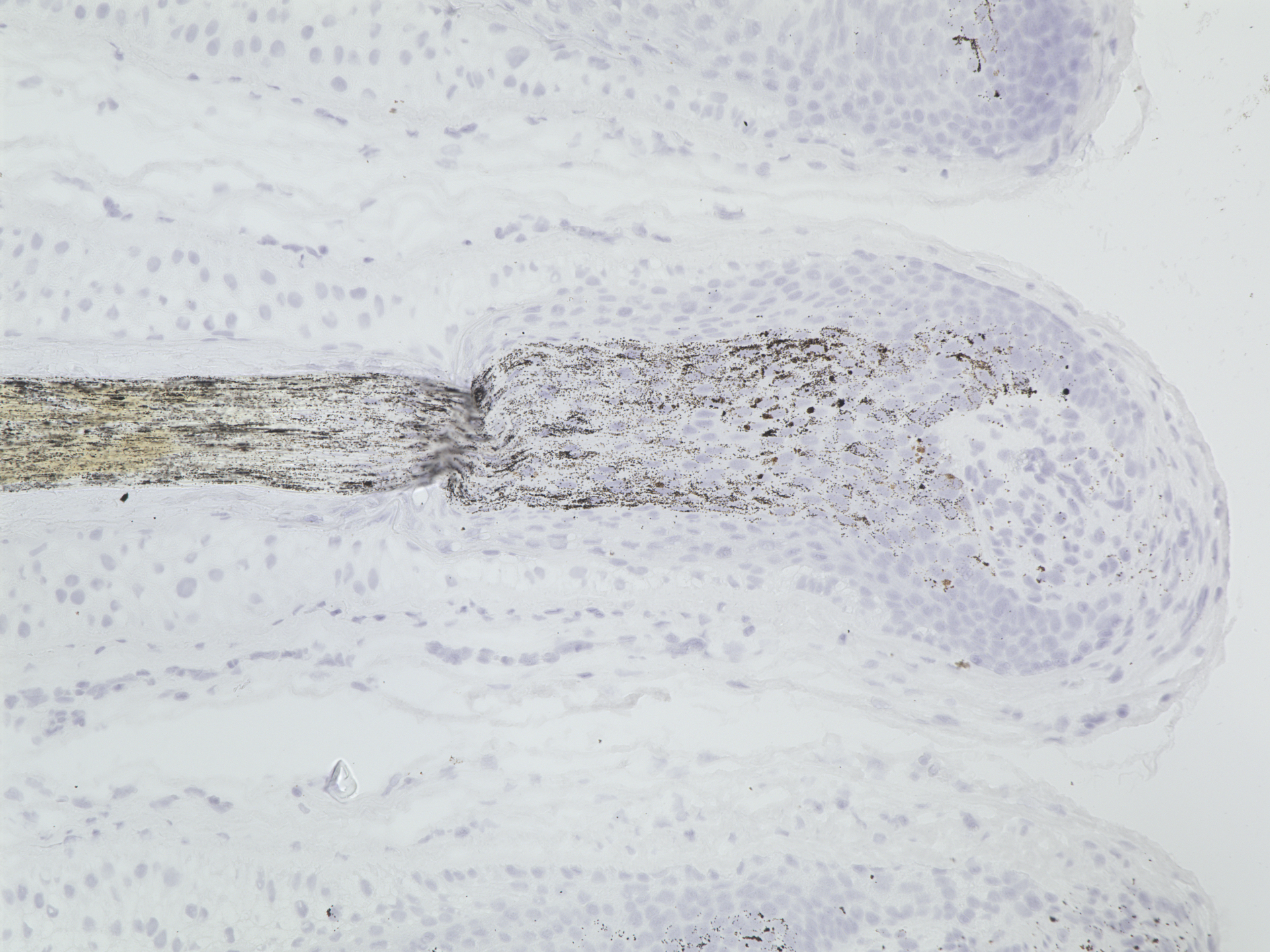

Supplement: Supplementary file 5 — Source Data for Figure 2 [file EMBR-24-e56574-s005.zip › 2R/2R Masson-Fontana Rapa+Agouti.tif]

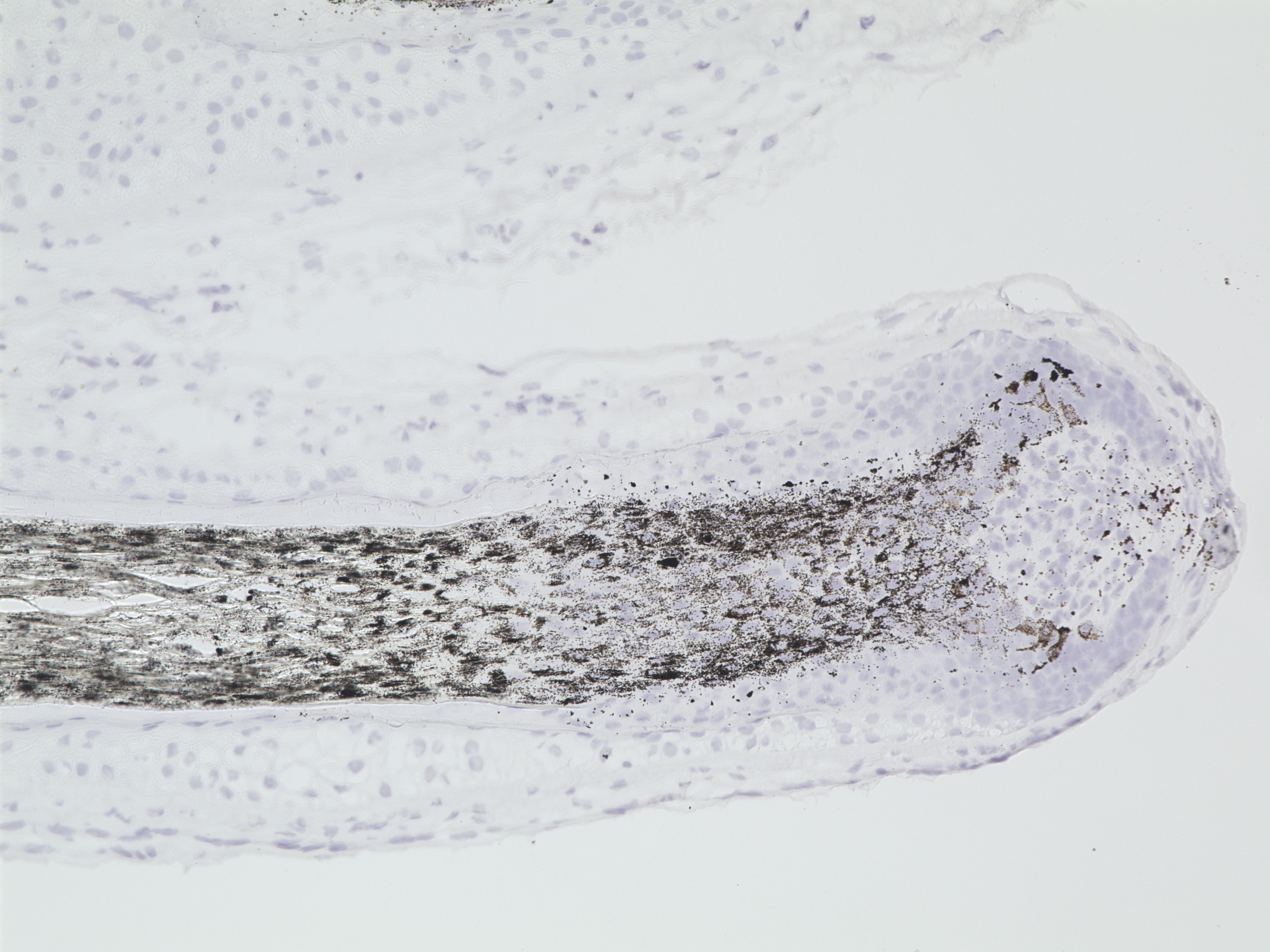

Supplement: Supplementary file 5 — Source Data for Figure 2 [file EMBR-24-e56574-s005.zip › 2R/2R Masson-Fontana Rapa.tif]

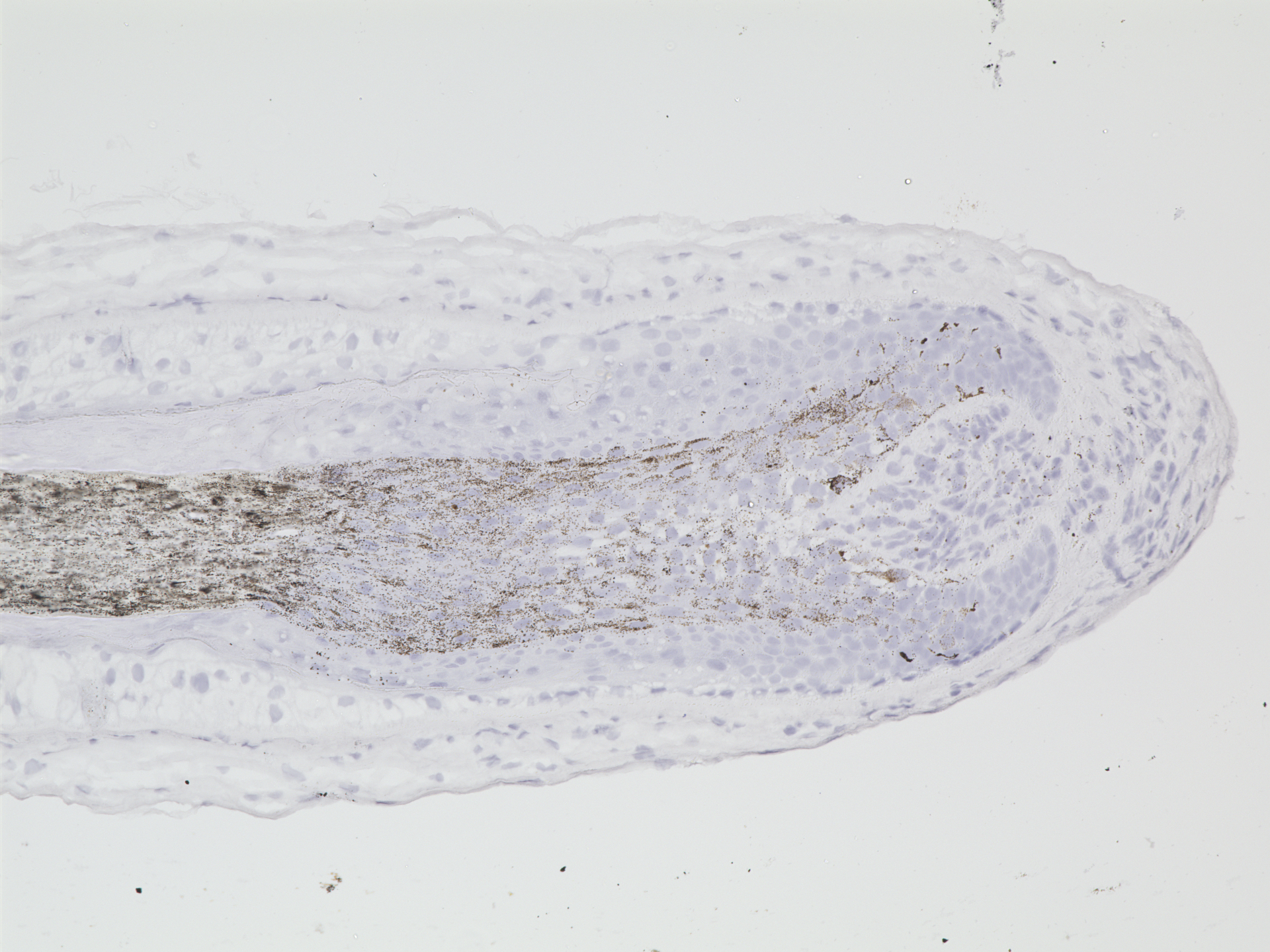

Supplement: Supplementary file 5 — Source Data for Figure 2 [file EMBR-24-e56574-s005.zip › 2R/2R Masson-Fontana Vehicle.tif]

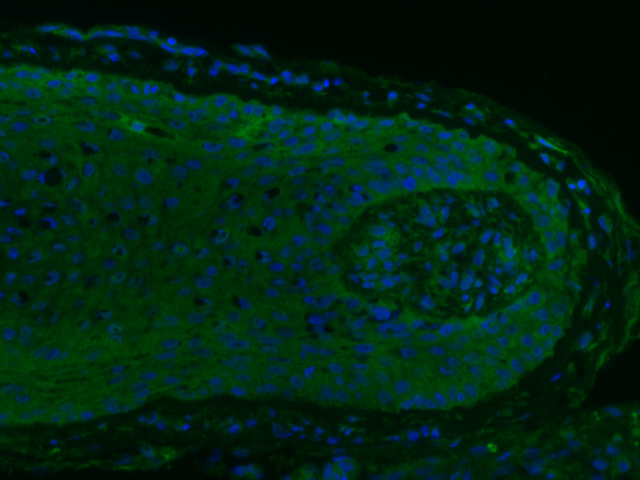

Supplement: Supplementary file 6 — Source Data for Figure 3 [file EMBR-24-e56574-s003.zip › 3C/3C TSC2 Non-targeting oligos_Overlay.tif]

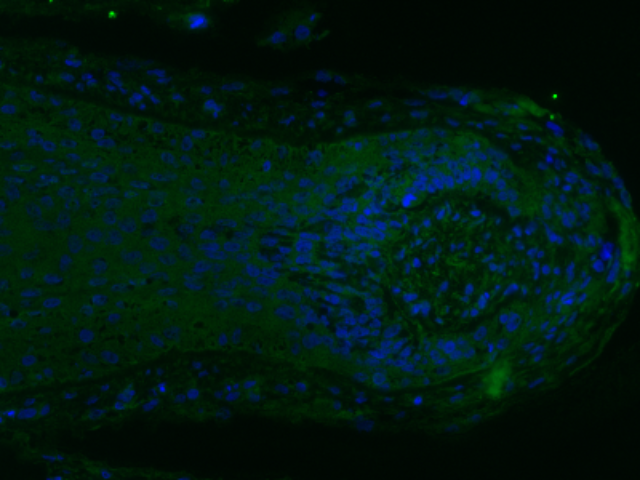

Supplement: Supplementary file 6 — Source Data for Figure 3 [file EMBR-24-e56574-s003.zip › 3C/3C TSC2 siTSC2_Overlay.tif]

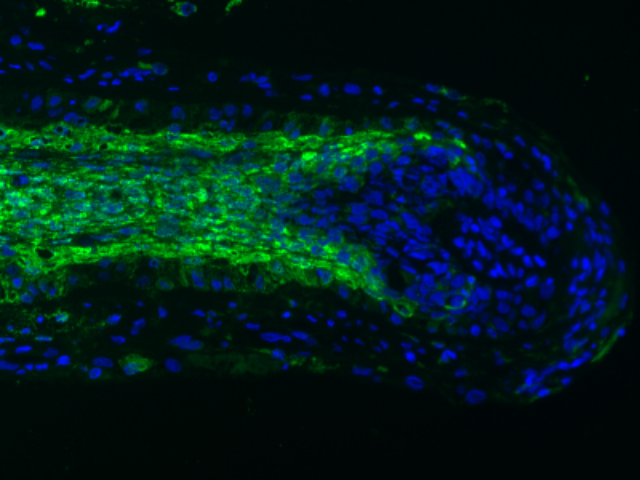

Supplement: Supplementary file 6 — Source Data for Figure 3 [file EMBR-24-e56574-s003.zip › 3E/3E pS6 Non-targeting Oligos_Overlay.tif]

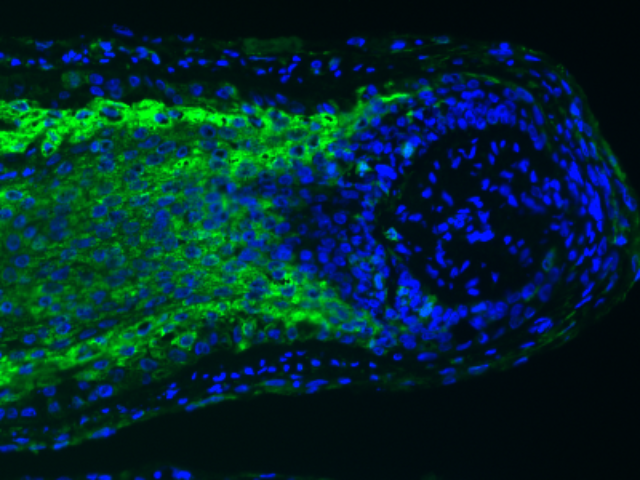

Supplement: Supplementary file 6 — Source Data for Figure 3 [file EMBR-24-e56574-s003.zip › 3E/3E pS6 siTSC2_Overlay.tif]

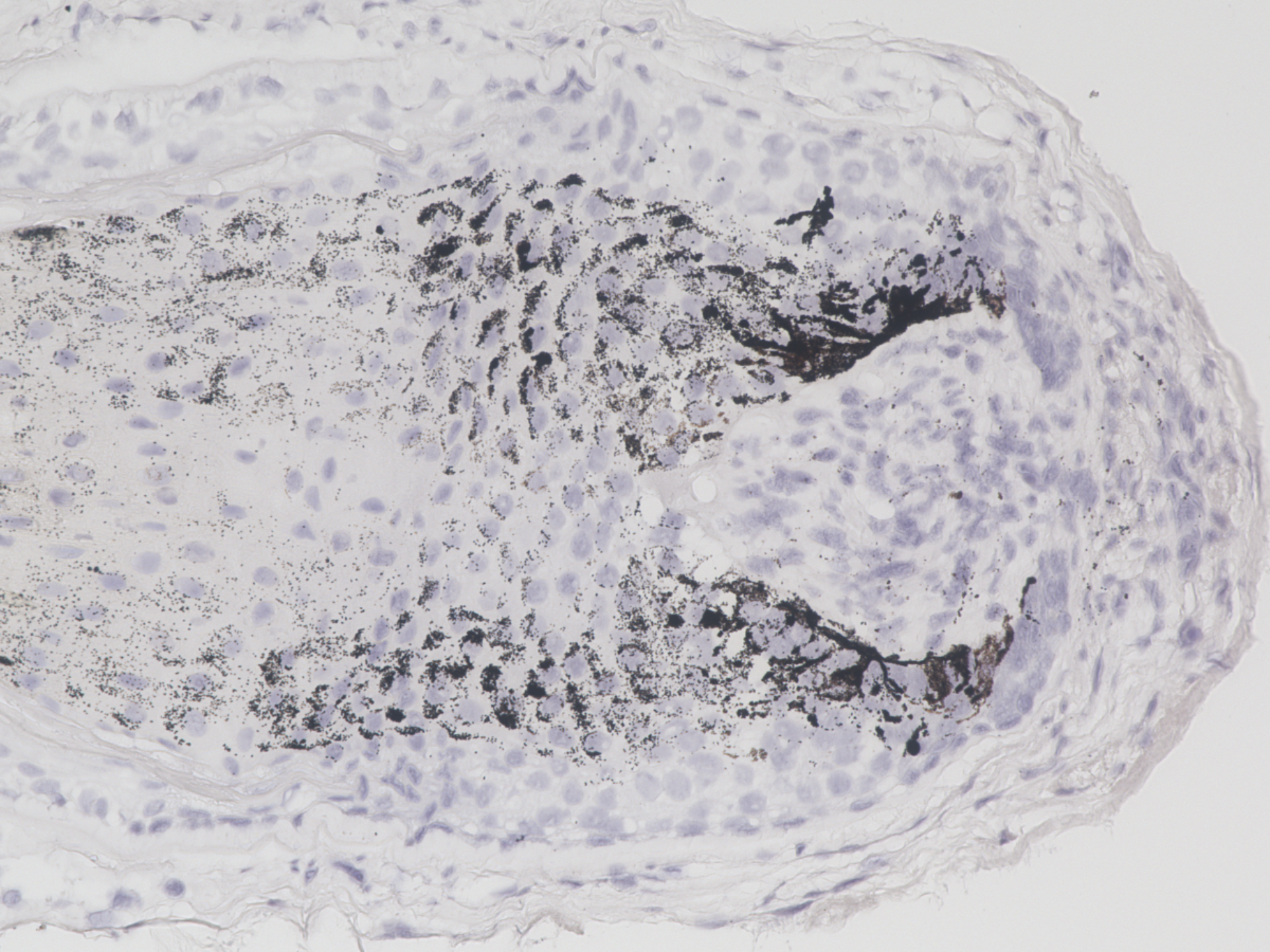

Supplement: Supplementary file 6 — Source Data for Figure 3 [file EMBR-24-e56574-s003.zip › 3G/3G Masson-Fontana Non-targeting Oligos.tif]

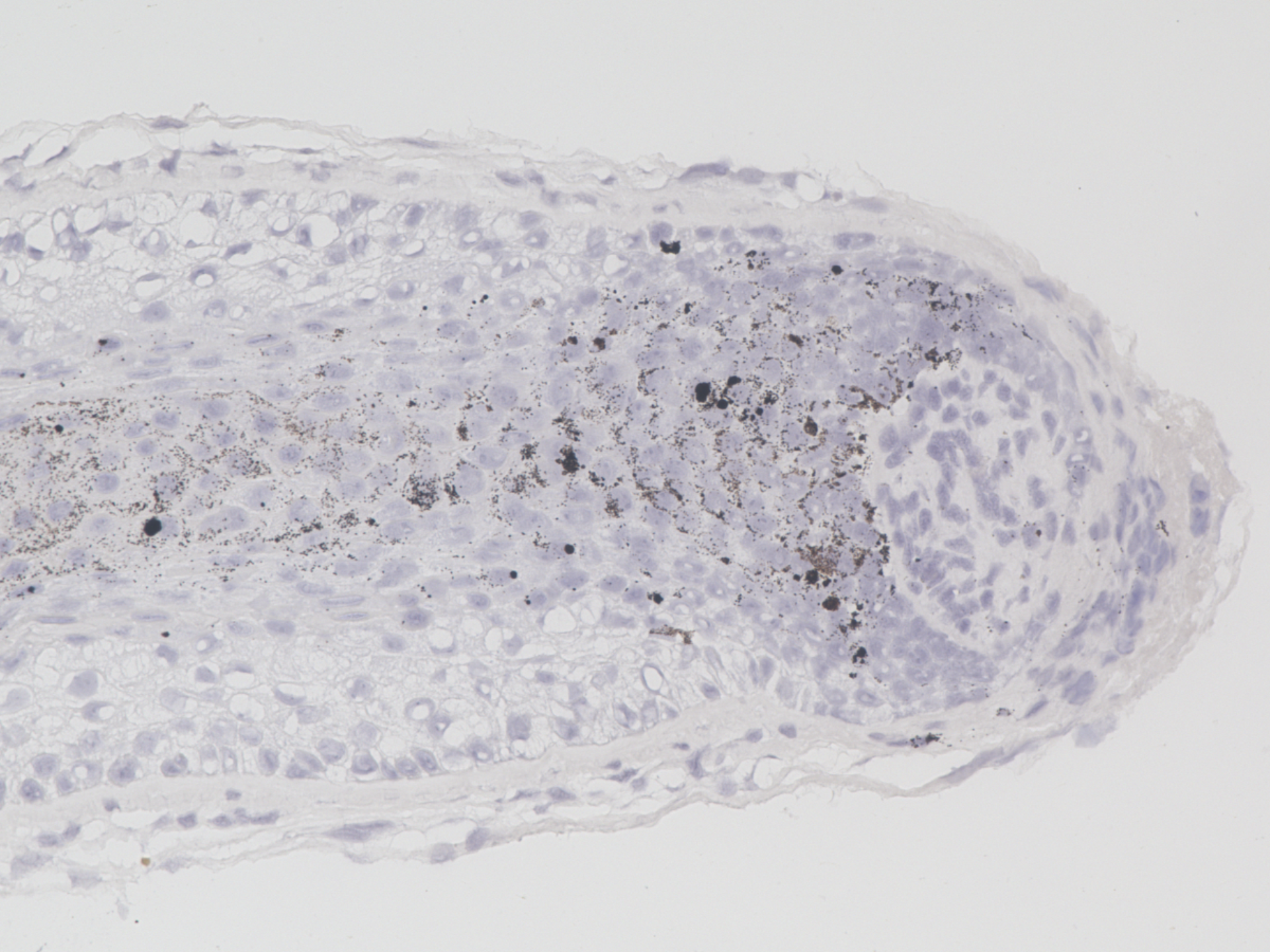

Supplement: Supplementary file 6 — Source Data for Figure 3 [file EMBR-24-e56574-s003.zip › 3G/3G Masson-Fontana siTSC2.tif]

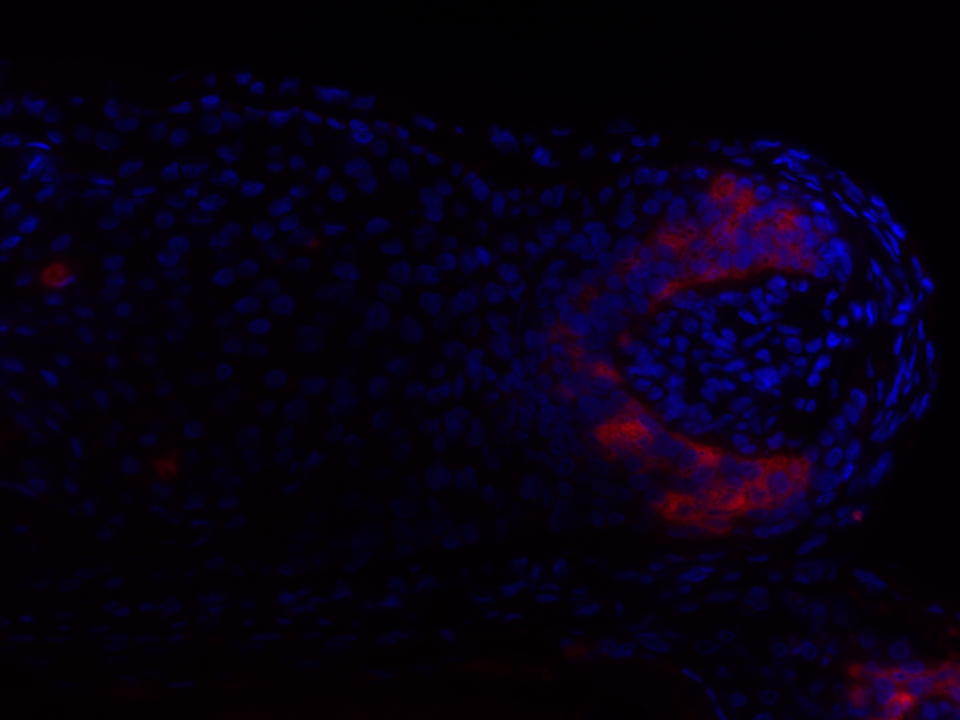

Supplement: Supplementary file 6 — Source Data for Figure 3 [file EMBR-24-e56574-s003.zip › 3I/3I Tyrosinase Non-targeting Oligos_Overlay.tif]

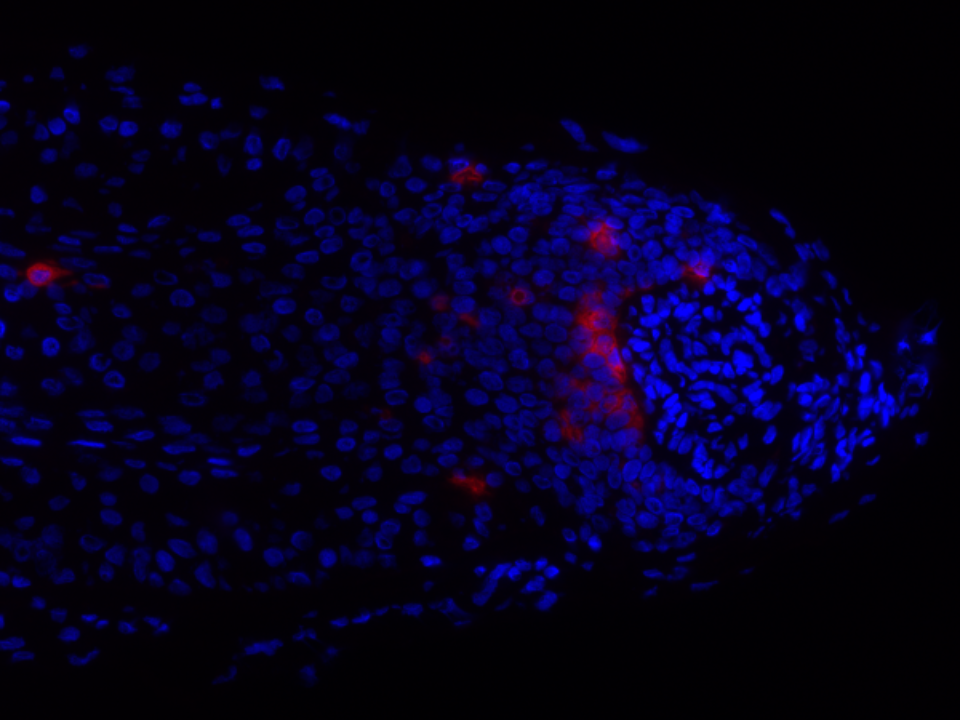

Supplement: Supplementary file 6 — Source Data for Figure 3 [file EMBR-24-e56574-s003.zip › 3I/3I Tyrosinase siTSC2_Overlay.tif]

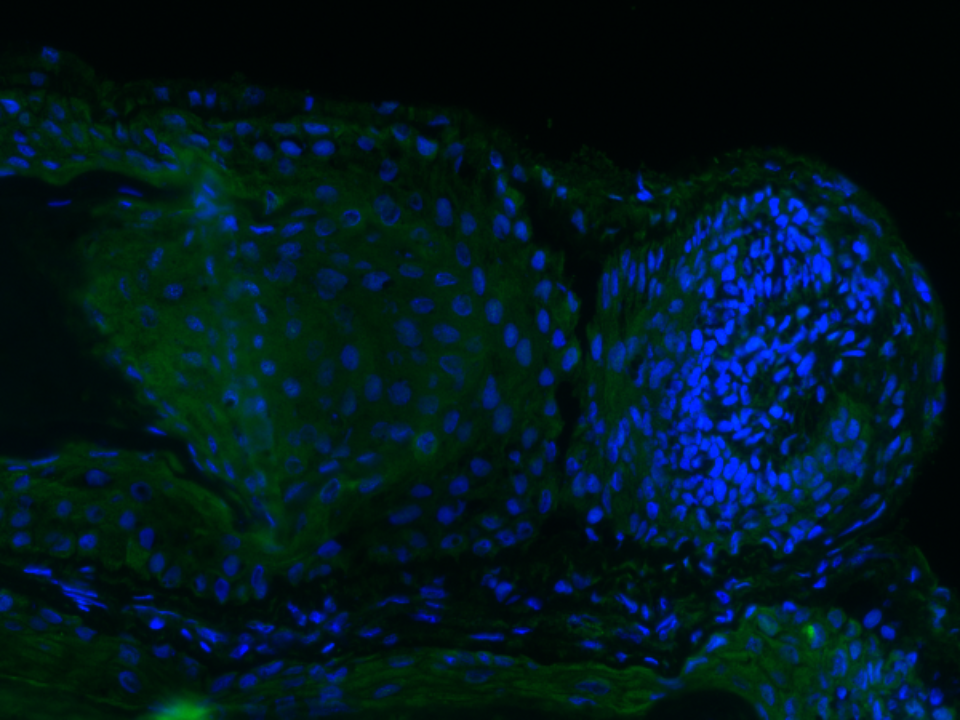

Supplement: Supplementary file 6 — Source Data for Figure 3 [file EMBR-24-e56574-s003.zip › 3K/3K aMSH Non-targeting Oligos_Overlay.tif]

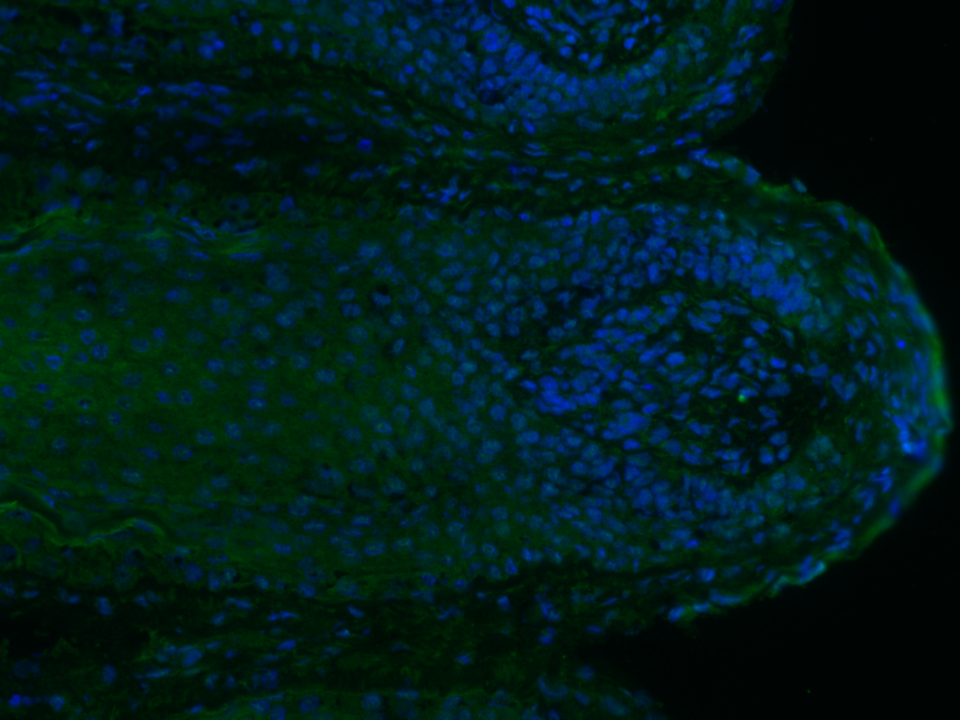

Supplement: Supplementary file 6 — Source Data for Figure 3 [file EMBR-24-e56574-s003.zip › 3K/3K aMSH siTSC2_Overlay.tif]

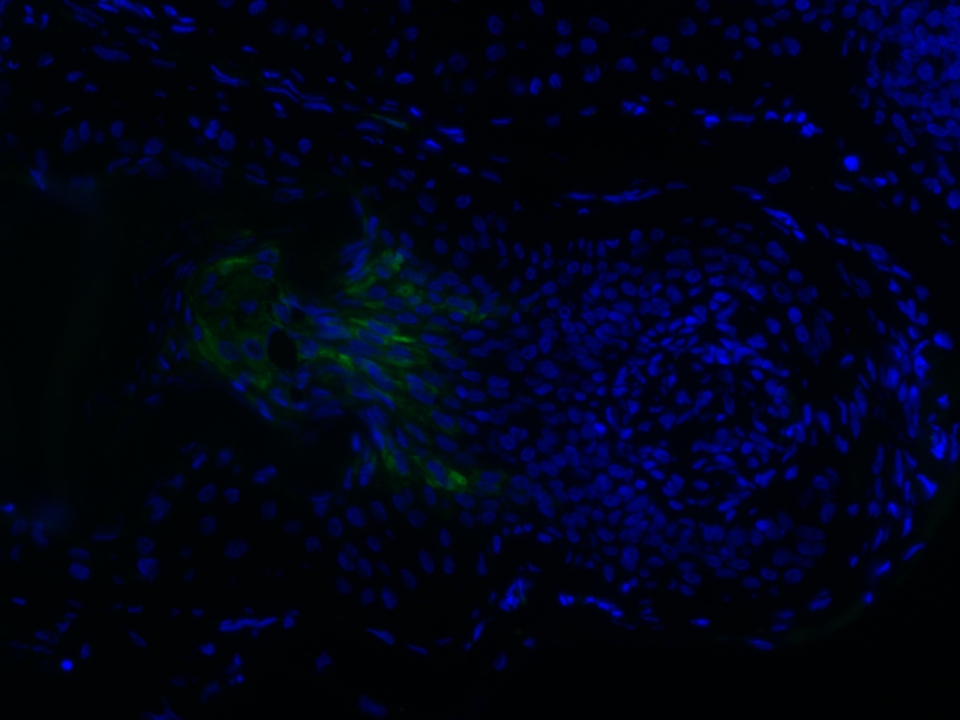

Supplement: Supplementary file 7 — Source Data for Figure 4 [file EMBR-24-e56574-s002.zip › 4B/4B pS6 Rapa.jpg]

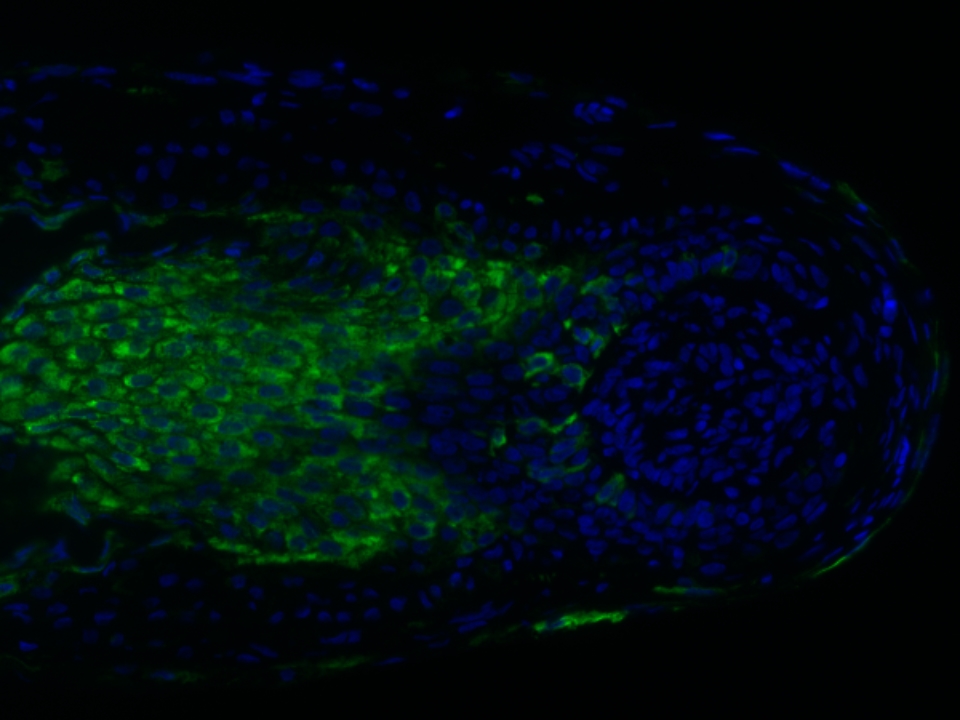

Supplement: Supplementary file 7 — Source Data for Figure 4 [file EMBR-24-e56574-s002.zip › 4B/4B pS6 Vehicle.jpg]

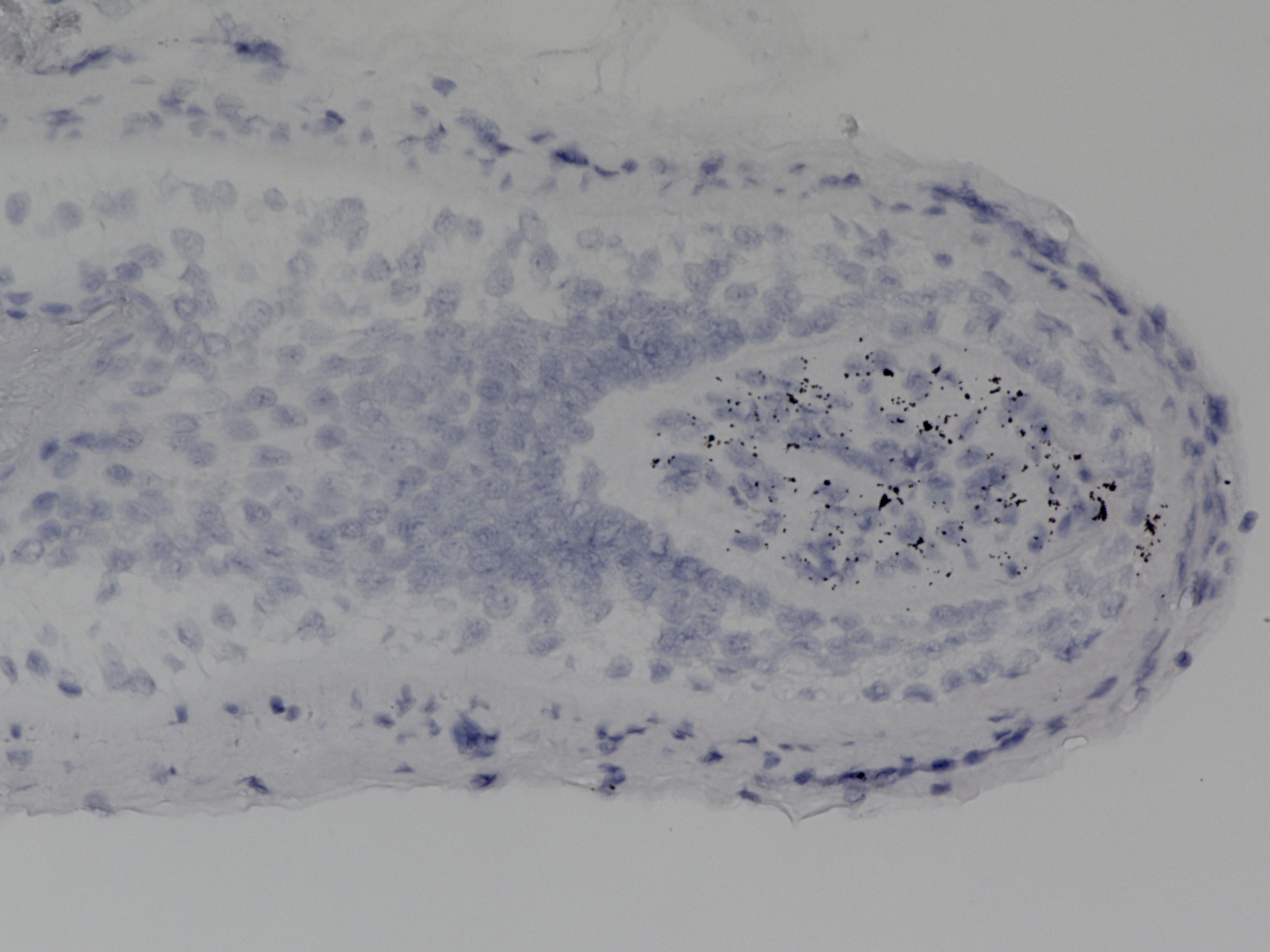

Supplement: Supplementary file 7 — Source Data for Figure 4 [file EMBR-24-e56574-s002.zip › 4D/4D Masson Fontana Rapa.tif]

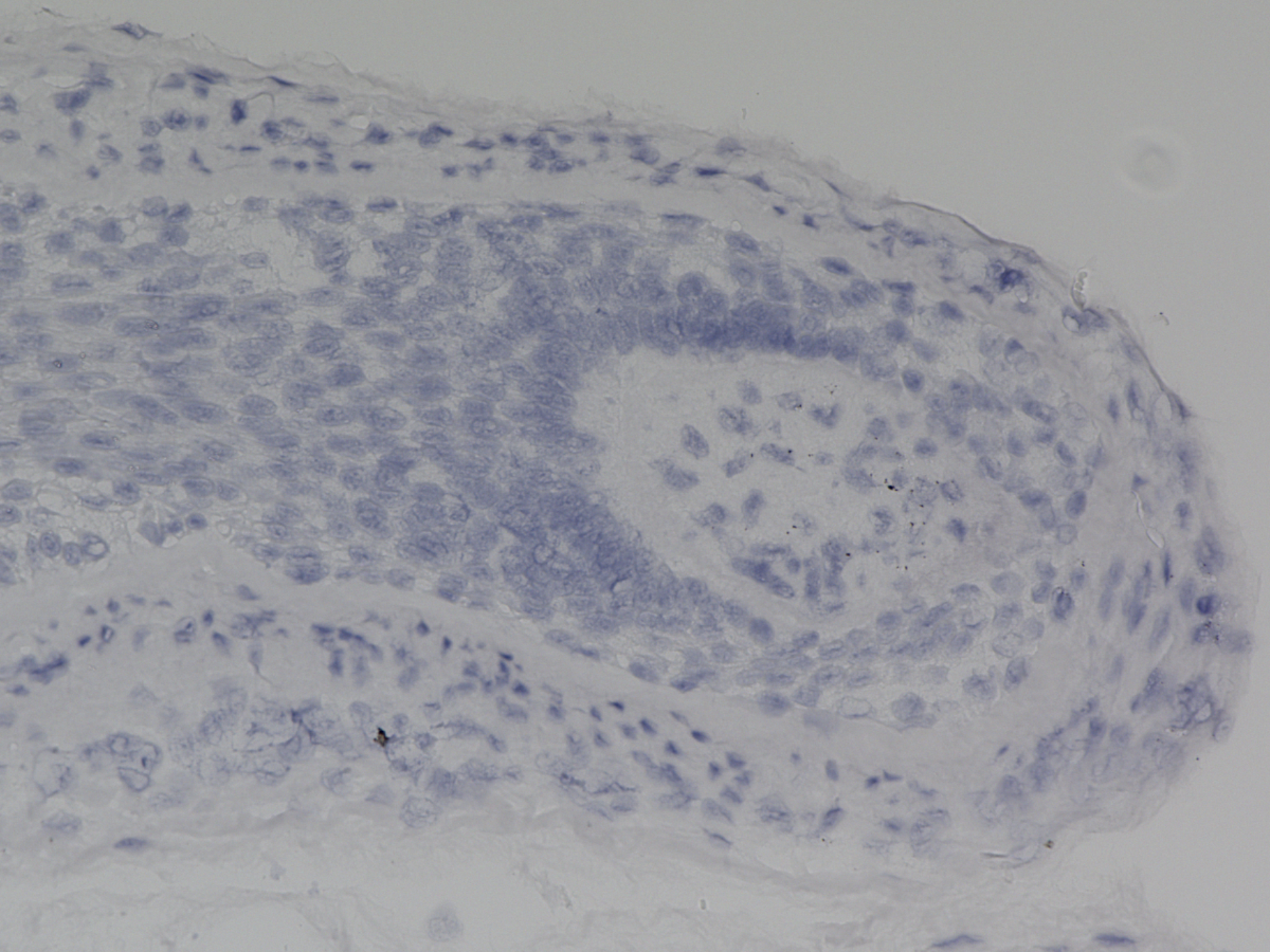

Supplement: Supplementary file 7 — Source Data for Figure 4 [file EMBR-24-e56574-s002.zip › 4D/4D Masson Fontana Vehicle.tif]

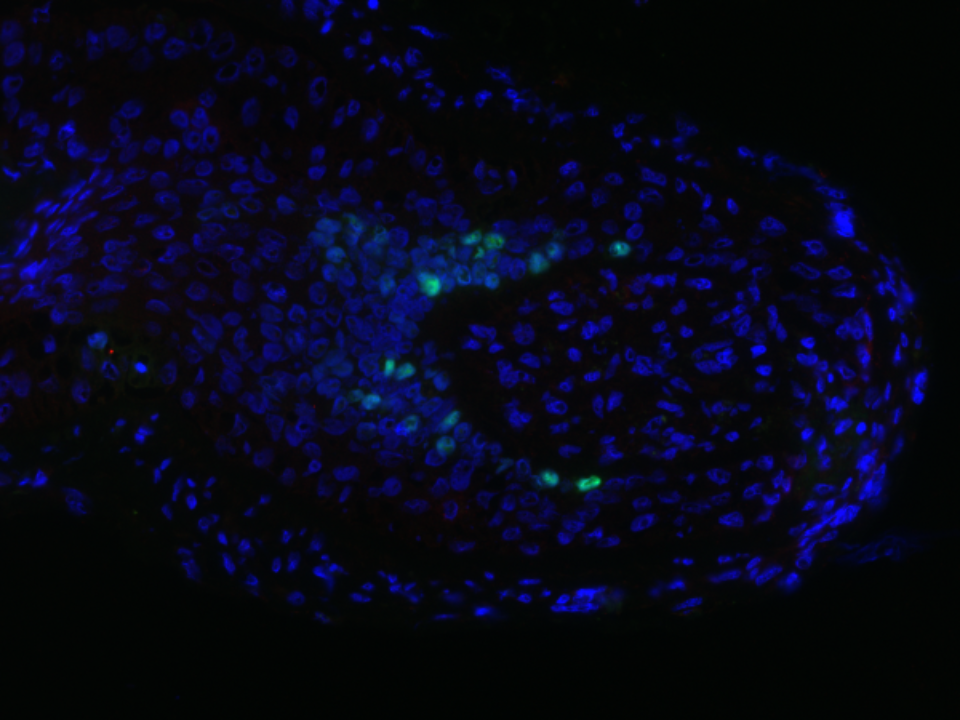

Supplement: Supplementary file 7 — Source Data for Figure 4 [file EMBR-24-e56574-s002.zip › 4D/4D Rapa Ki67.tif]

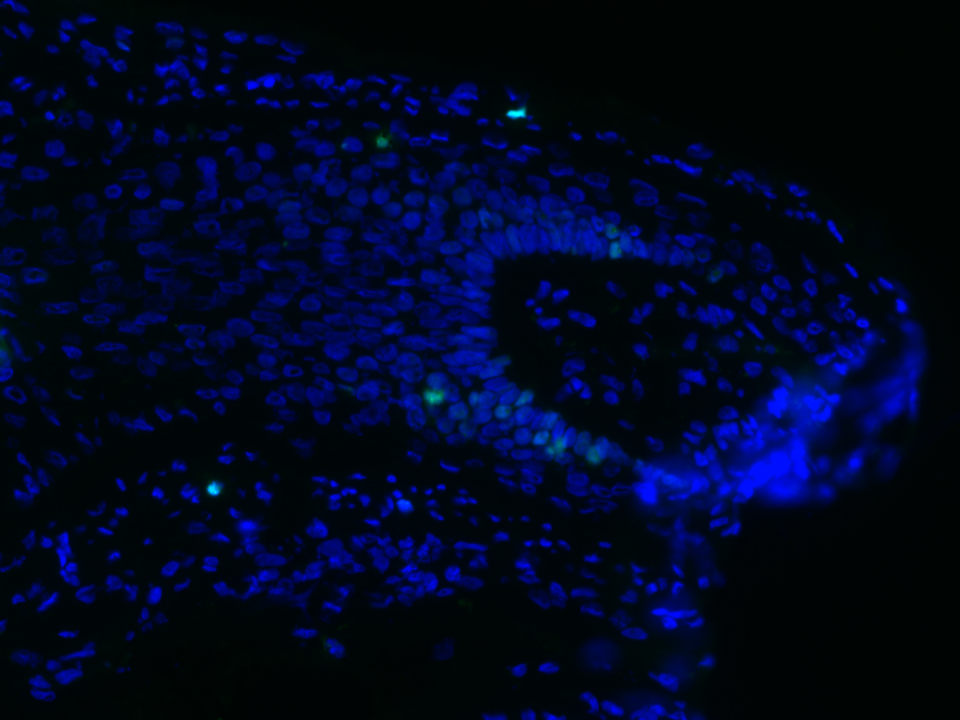

Supplement: Supplementary file 7 — Source Data for Figure 4 [file EMBR-24-e56574-s002.zip › 4D/4D Vehicle Ki67.tif]

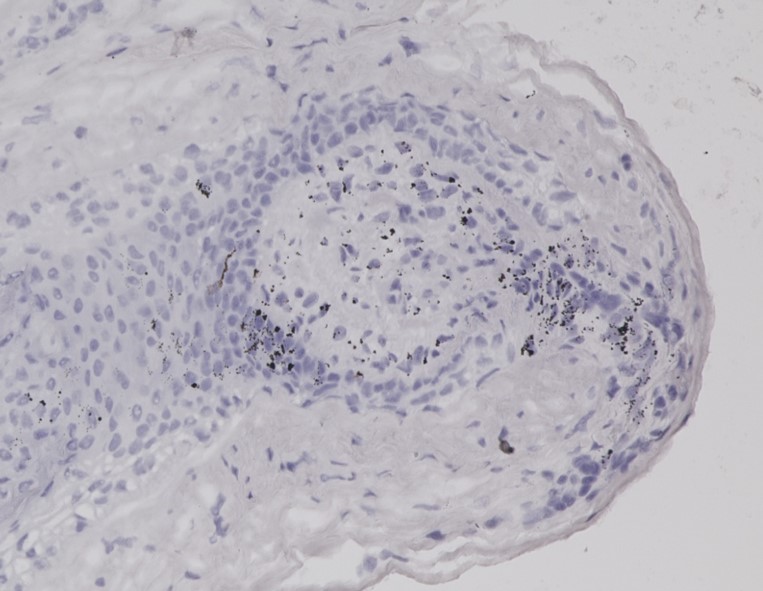

Supplement: Supplementary file 7 — Source Data for Figure 4 [file EMBR-24-e56574-s002.zip › 4F/4F Rapa Masson Fontana.jpg]

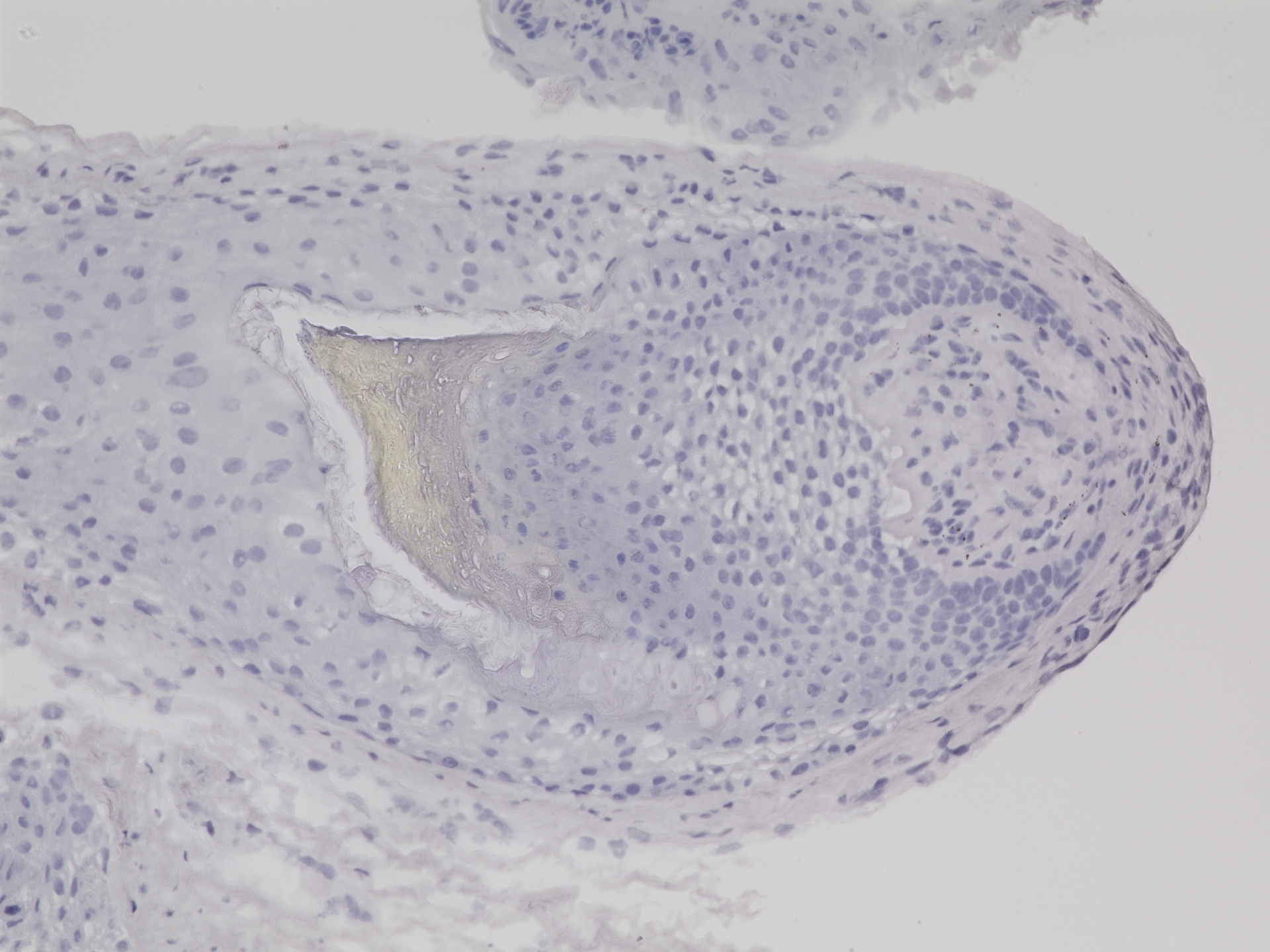

Supplement: Supplementary file 7 — Source Data for Figure 4 [file EMBR-24-e56574-s002.zip › 4F/4F Vehicle Masson Fontana.jpg]

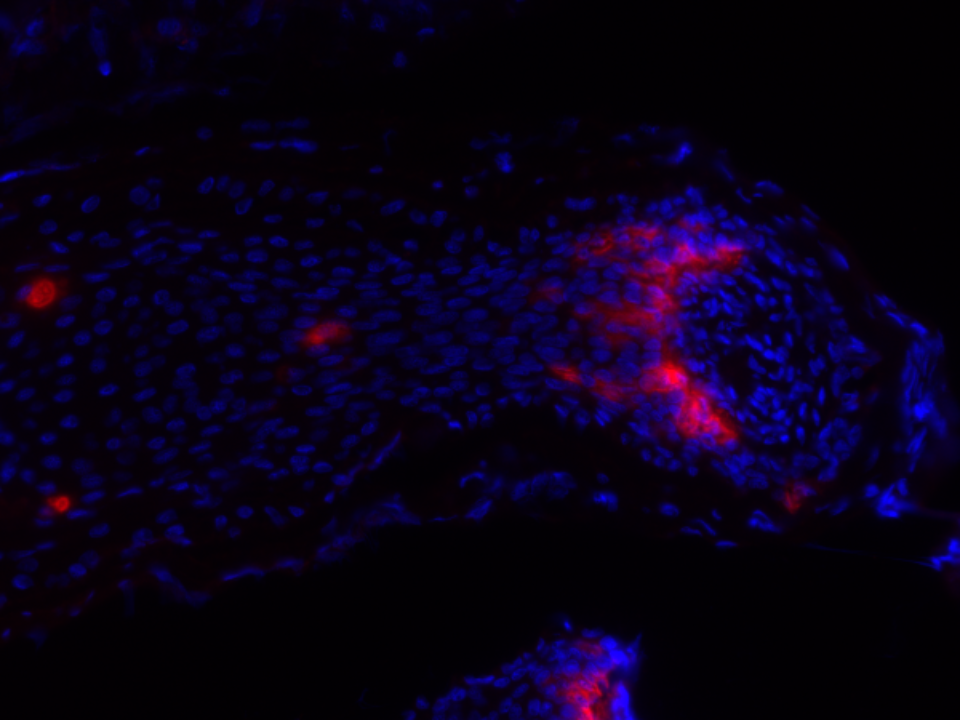

Supplement: Supplementary file 7 — Source Data for Figure 4 [file EMBR-24-e56574-s002.zip › 4H/4H Tyrosinase Rapa.tif]

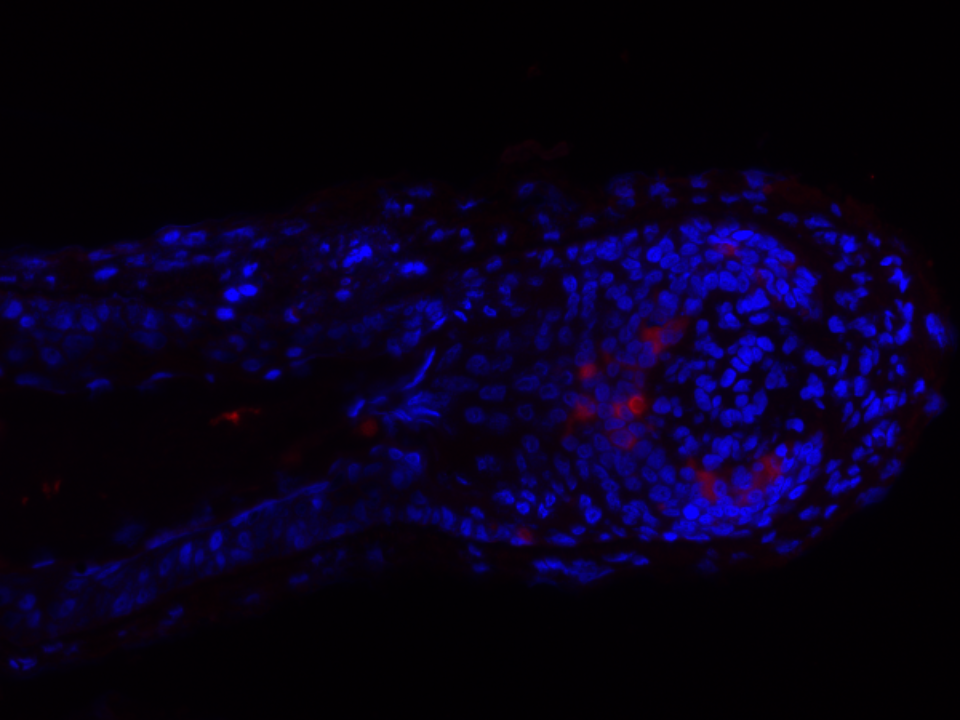

Supplement: Supplementary file 7 — Source Data for Figure 4 [file EMBR-24-e56574-s002.zip › 4H/4H Tyrosinase Vehicle.tif]

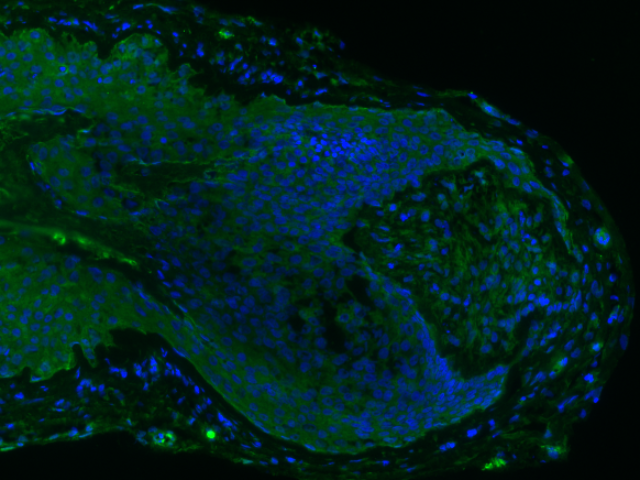

Supplement: Supplementary file 7 — Source Data for Figure 4 [file EMBR-24-e56574-s002.zip › 4J/4J aMSH Rapa.tif]

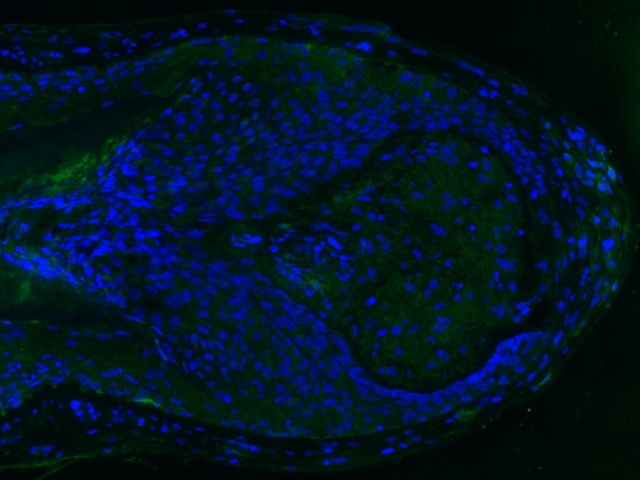

Supplement: Supplementary file 7 — Source Data for Figure 4 [file EMBR-24-e56574-s002.zip › 4J/4J aMSH Vehicle.tif]
