# Supplementary material for: mTORC1 activity negatively regulates human hair follicle growth and pigmentation
Source: EMBO Rep. 2023 May 22;24(7):e56574. doi: 10.15252/embr.202256574 (PMC10328083; doi:10.15252/embr.202256574)
Supplement: Supplementary file 8 [file EMBR-24-e56574-s006.pdf]

EMBO reports

mTORC1 activity negatively regulates human hair follicle growth and pigmentation

Takahiro Suzuki, Jérémy Chéret, Fernanda Scala, Aysun Akhundlu, Jennifer Gherardini, Dana-Lee Demetrius, James O'Sullivan, Gorana Epstein-Kuka, Alan Bauman, Constantinos Demetriades, and Ralf Paus
DOI: 10.15252/embr.202256574

Corresponding author(s): Ralf Paus (rxp803@med.miami.edu)

Review Timeline:

Submission Date:	28th Nov 22
Editorial Decision:	23rd Jan 23
Revision Received:	1st Mar 23
Editorial Decision:	23rd Mar 23
Revision Received:	11th Apr 23
Accepted:	24th Apr 23

Editor: Martina Rembold

Transaction Report:

(Note: With the exception of the correction of typographical or spelling errors that could be a source of ambiguity, letters and reports are not edited. Depending on transfer agreements, referee reports obtained elsewhere may or may not be included in this compilation. Referee reports are anonymous unless the Referee chooses to sign their reports.)

1st Editorial Decision **23rd Jan 2023**

Dear Dr. Paus

Thank you for the submission of your research manuscript to our journal. We have now received the full set of referee reports that is copied below.

As you will see, both referees consider the findings interesting but they also raise a number of concerns regarding missing control experiments, missing experimental details and clarity, or quantification, which need to be addressed.

Given these constructive comments, we would like to invite you to revise your manuscript with the understanding that the referee concerns (as detailed above and in their reports) must be fully addressed and their suggestions taken on board. Please address all referee concerns in a complete point-by-point response. Acceptance of the manuscript will depend on a positive outcome of a second round of review. It is EMBO reports policy to allow a single round of revision only and acceptance or rejection of the manuscript will therefore depend on the completeness of your responses included in the next, final version of the manuscript.

We realize that it is difficult to revise to a specific deadline. In the interest of protecting the conceptual advance provided by the work, we recommend a revision within 3 months (April 23rd). Please discuss the revision progress ahead of this time with me if you require more time to complete the revisions.

I am also happy to discuss the revision further via e-mail or a video call, if you wish.

Your article will be published as a short report (unless the revision has more than 5 figures). For short reports, the revised manuscript should not exceed 27,000 characters (including spaces but excluding materials & methods and references) and 5 main plus 5 expanded view figures. The results and discussion sections must further be combined, which will help to shorten the manuscript text by eliminating some redundancy that is inevitable when discussing the same experiments twice.

*****IMPORTANT NOTE:
We perform an initial quality control of all revised manuscripts before re-review. Your manuscript will FAIL this control and the handling will be DELAYED if the following APPLIES:

1) A data availability section providing access to data deposited in public databases is missing. If you have not deposited any data, please add a sentence to the data availability section that explains that.

2) Your manuscript contains statistics and error bars based on n=2. Please use scatter blots in these cases. No statistics should be calculated if n=2.

When submitting your revised manuscript, please carefully review the instructions that follow below. Failure to include requested items will delay the evaluation of your revision.*****

When submitting your revised manuscript, we will require:

1) a .docx formatted version of the manuscript text (including legends for main figures, EV figures and tables). Please make sure that the changes are highlighted to be clearly visible.

2) individual production quality figure files as .eps, .tif, .jpg (one file per figure).
Please download our Figure Preparation Guidelines (figure preparation pdf) from our Author Guidelines pages https://www.embopress.org/page/journal/14693178/authorguide for more info on how to prepare your figures.

3) a .docx formatted letter INCLUDING the reviewers' reports and your detailed point-by-point responses to their comments. As part of the EMBO Press transparent editorial process, the point-by-point response is part of the Review Process File (RPF), which will be published alongside your paper.

4) a complete author checklist, which you can download from our author guidelines (). Please insert information in the checklist that is also reflected in the manuscript. The completed author checklist will also be part of the RPF.

5) Please note that all corresponding authors are required to supply an ORCID ID for their name upon submission of a revised manuscript (). Please find instructions on how to link your ORCID ID to your account in our manuscript tracking system in our Author guidelines
()

6) We replaced Supplementary Information with Expanded View (EV) Figures and Tables that are collapsible/expandable online. A maximum of 5 EV Figures can be typeset. EV Figures should be cited as 'Figure EV1, Figure EV2" etc... in the text and their respective legends should be included in the main text after the legends of regular figures.

- For the figures that you do NOT wish to display as Expanded View figures, they should be bundled together with their legends in a single PDF file called *Appendix*, which should start with a short Table of Content. Appendix figures should be referred to in the main text as: "Appendix Figure S1, Appendix Figure S2" etc. See detailed instructions regarding expanded view here:

- Additional Tables/Datasets should be labeled and referred to as Table EV1, Dataset EV1, etc. Legends have to be provided in a separate tab in case of .xls files. Alternatively, the legend can be supplied as a separate text file (README) and zipped together with the Table/Dataset file.

7) Please note that a Data Availability section at the end of Materials and Methods is now mandatory. In case you have no data that requires deposition in a public database, please state so instead of refereeing to the database.
See also < https://www.embopress.org/page/journal/14693178/authorguide#dataavailability>). Please note that the Data Availability Section is restricted to new primary data that are part of this study.

8) At EMBO Press we ask authors to provide source data for the main figures. Our source data coordinator will contact you to discuss which figure panels we would need source data for and will also provide you with helpful tips on how to upload and organize the files.

Additional information on source data and instruction on how to label the files are available .

9) The journal requires a statement specifying whether or not authors have competing interests (defined as all potential or actual interests that could be perceived to influence the presentation or interpretation of an article). In case of competing interests, this must be specified in your disclosure statement. Further information: https://www.embopress.org/competing-interests

10) Figure legends and data quantification:
The following points must be specified in each figure legend:

- the name of the statistical test used to generate error bars and P values,
- the number (n) of independent experiments (please specify technical or biological replicates) underlying each data point,
- the nature of the bars and error bars (s.d., s.e.m.)

- If the data are obtained from n {less than or equal to} 5, show the individual data points in addition to the SD or SEM.
- If the data are obtained from n {less than or equal to} 2, use scatter blots showing the individual data points.

Discussion of statistical methodology can be reported in the materials and methods section, but figure legends should contain a basic description of n, P and the test applied.

See also the guidelines for figure legend preparation:
https://www.embopress.org/page/journal/14693178/authorguide#figureformat

- Please also include scale bars in all microscopy images.

11) Our journal encourages inclusion of *data citations in the reference list* to directly cite datasets that were re-used and obtained from public databases. Data citations in the article text are distinct from normal bibliographical citations and should directly link to the database records from which the data can be accessed. In the main text, data citations are formatted as follows: "Data ref: Smith et al, 2001" or "Data ref: NCBI Sequence Read Archive PRJNA342805, 2017". In the Reference list, data citations must be labeled with "[DATASET]". A data reference must provide the database name, accession number/identifiers and a resolvable link to the landing page from which the data can be accessed at the end of the reference. Further instructions are available at .

12) As part of the EMBO publication's Transparent Editorial Process, EMBO reports publishes online a Review Process File to accompany accepted manuscripts. This File will be published in conjunction with your paper and will include the referee reports, your point-by-point response and all pertinent correspondence relating to the manuscript.

You are able to opt out of this by letting the editorial office know (emboreports@embo.org). If you do opt out, the Review Process File link will point to the following statement: "No Review Process File is available with this article, as the authors have chosen not to make the review process public in this case."

We would also welcome the submission of cover suggestions, or motifs to be used by our Graphics Illustrator in designing a

cover.

I look forward to seeing a revised form of your manuscript when it is ready and please let me know if you have questions or comments regarding the revision.

You can use this link to submit your revision: https://embor.msubmit.net/cgi-bin/main.plex

Yours sincerely,

Martina Rembold, PhD
Senior Editor
EMBO reports

****

Referee #1:

In this manuscript, the authors report on the role of TORC1 as a regulator of hair pigmentation and even growth using a whole organ culture of human hair follicles.
In their findings the authors show that mTORC1 is significantly upregulated during hair greying. Notably, melanogenesis is increased when mTORC1 is inhibited with rapamycin- even in hair greying follicles that have not reached the point of irreversibility , whereas hair pigmentation decreased when mTORC1 is overexpressed. In an effort to find the mechanism behind the regulation of melanogenesis by mTORC1, the authors found that mTORC1 inhibition stimulated intrafollicular MSH production and activation of MC1R. Interestingly, this control of melanogenesis is linked to a prolongation of the hair cycle anagen phase and therefore hinting that rapamycin has potential as a hair growth stimulator by extending the anagen phase.
Overall, this manuscript is a solid hypothesis-driven study where the authors make good use of their ex vivo human hair follicle culture model to uncover promising strategies to stimulate hair pigmentation during normal pigmented or hair greying follicles by inhibiting mTORC1. Therefore this work will be catching the attention and interest from general and specialized audiences.

Minor concerns/comments:

According to the authors, the increase of melanogenesis by rapamycin induced
mTORC1 inhibition is driven by stimulating intrafollicular tyrosinase activity and melanin transfer. And, this increase in melanogenesis is enhanced by -MSH production and activation of MC1R. Interestingly, this is not primarily mediated by MITF. Here the authors measure -MSH expression by (immuno-)histomorphometry and also block MC1R activity using ASP, however it would be interesting to measure MC1R expression as well. Could the authors provide quantification of MC1R expression before and mTORC1 inhibition?. In addition, this reviewer finds the sentence "Contrary to a widespread misconception, human hair greying does not primarily result from a dysfunction of HF melanocyte stem cells, but is initiated by a dysfunction of differentiated melanocytes in the HFPU" interesting and provocative but unsupported by the single reference provided (O'Sullivan et al, 2021). In fact, it opens the question whether mTORC1- or its inhibition- is also regulating the differentiation of HF melanocyte stem cells. Given that their ex vivo model may not include the HF stem cell compartment , the authors could try to the measure tyrosinase activity of the more undifferentiated melanocytes found in the upper ORS layers after mTORC1 inhibition?

Referee #2:

In this study, Suzuki et al. have revealed a role of mTORC1 on ex vivo hair follicle (HF) pigmentation combining elegant models of HF organ culture with molecular analyses. These are novel and important findings, especially in the context of human HF biology. Given that HF pigmentation is the result of multiple intracellular pathways in melanocytes together with highly regulated interactions between melanocytes and keratinocytes, the observations reported are of interest well beyond the fields of cutaneous biology. Indeed, many human biological and pathophysiological aspects are modulated or the result of complex cell-cell and cell-environment interactions, aspects of which can be modeled by the HF system. The body of work presented is extensive, and the data support the major conclusions of the paper. That being said, some details of the studies are lacking throughout the manuscript and, consequently, it is difficult to assess whether several conclusions drawn from some experiments are validated by the data shown, as detailed below.

1. Although the differences in p-S6 fluorescence intensity are obvious in Fig. 1, there is no description included concerning how images were acquired and fluorescence signals were quantified to ensure accuracy of measurements and conclusions. This aspect of the work needs to be addressed. Similar considerations are relevant for other experiments involving quantification of fluorescence signals.

2. Figure 2D appears to be incomplete, as micrographs of Catagen HF treated with rapamycin and Anagen HF treated with vehicle have been omitted. The description of the experimental methods appears to indicate that all HF used in these studies were in Anagen VI.

3. Although it is well known that melanocytes develop dendrites, the micrographs included do not allow evaluation of the abundance of dendrites in said melanocytes. Please include clear micrographs that demonstrate melanocyte dendricity in the tissue sections.

4. The conclusions regarding the effect of rapamycin on melanosome transfer to HF keratinocytes (p.8) are not supported by the data shown, as the experiments presented are not designed to measure melanosome transfer from melanocytes to keratinocytes.

5. The mechanistic experiments that included TSC silencing are very important. It is somewhat unusual that a relatively minor approximately 20% decrease in TSC2 abundance (as per immunofluorescence quantification shown in Fig. 3B, C would be associated with a major downstream effect. This should be further discussed.

6. The description of drug treatments is not clear and needs to be revised (P. 18). Specifically, the drugs were administered several times. However, it is not clear if a stock solution of 20 ng/ml rapamycin was used, or if the final concentration in the culture medium was 20 ng/ml. Similar considerations apply to ASIP and DMSO. Further, it is not clear whether there were changes in the culture medium, so that rapamycin was always present at the same concentration, or whether there were simply additions to the existing culture medium, in which case the drug and vehicle concentrations varied from day 1 to day 7. The micrographs appear to indicate that the HF were cultured in the presence of 20 ng/ml rapamycin, but the description in the "Methods" section is not very clear.

7. Similarly, for siRNA experiments it is not clear whether a 1 M stock solution (non-specified volume) was used, or the follicles were culture for 48 h in the presence of 1 M siRNA.

Minor points:
1. P4: "...TSC loss-of-function in mouse tissues and cultured fibroblasts show upregulated..." should read "...TSC loss-of-function in mouse tissues and cultured fibroblasts shows upregulated..."

2. The micrographs in Fig. 2H are not of very good quality. Please replace.

Editor comments:

1) A data availability section providing access to data deposited in public databases is missing. If you have not deposited any data, please add a sentence to the data availability section that explains that.

We have now explained in the data availability section that we have not deposited any data: "*This study includes no data deposited in external repositories.*"

2) Your manuscript contains statistics and error bars based on n=2. Please use scatter blots in these cases. No statistics should be calculated if n=2.
Thanks, done as requested.
We have now exchanged the graph in questions to show scatter blots (see new Fig 3D)

Referee #1:

Overall, this manuscript is a solid hypothesis-driven study where the authors make good use of their ex vivo human hair follicle culture model to uncover promising strategies to stimulate hair pigmentation during normal pigmented or hair greying follicles by inhibiting mTORC1. Therefore this work will be catching the attention and interest from general and specialized audiences.
We thank the reviewer for these very encouraging comments.

Minor concerns/comments:

According to the authors, the increase of melanogenesis by rapamycin induced mTORC1 inhibition is driven by stimulating intrafollicular tyrosinase activity and melanin transfer. And, this increase in melanogenesis is enhanced by a-MSH production and activation of MC1R. Interestingly, this is not primarily mediated by MITF. Here the authors measure a-MSH expression by (immuno-)histomorphometry and also block MC1R activity using ASIP, however it would be interesting to measure MC1R expression as well. Could the authors provide quantification of MC1R expression before and after mTORC1 inhibition?

Thank you for this suggestion. Looking at MC1R expression before and after rapamycin treatment would be interesting to investigate. Unfortunately, due to the limited amount of sections one can obtain from each hair follicle for immunohistology (5-6 sections), we cannot perform this additional analysis since we have exhausted all available sections for the different markers presented in our manuscript.
However, the expression level of MC1R in human hair follicles has been extensively characterized before by us and other (e.g., Ito et al 2005; Kauser et al, 2005, Böhm et al 2005). Since this additional analysis would not add substantial new insights into the mechanistic data already presented in the context of our study, this would not justify having to run new HF organ cultures. However, we now explicitly acknowledge in the revised Discussion (p. 14) that *"It would be intriguing to assess in a follow-up study whether rapamycin or TSC2 siRNA also impact on the levels of MC1R protein expression and/or signaling activity."*

In addition, this reviewer finds the sentence "Contrary to a widespread misconception, human hair greying does not primarily result from a dysfunction of HF melanocyte stem cells, but is initiated by a dysfunction of differentiated melanocytes in the HFPU" interesting and provocative but unsupported by the single reference provided (O'Sullivan et al, 2021). In fact, it opens the question whether mTORC1- or its inhibition- is also regulating the differentiation of HF melanocyte stem cells.

The authoritative review cited here (O'sullivan et al. 2021) explains extensively why human HF greying begins in and is driven by defective melanogenesis and melanin transfer in the HF pigmentary unit, with a dysfunction of HF melanocyte stem cells coming into play only fairly late during the hair greying process, namely when greying ultimately becomes irreversible. This had already been explained in detail in Paus, Pigm Cell Melanoma Res 2011, and has recently been underscored by our finding that even greying HFs can undergo extensive cycles of repigmentation and depigmentation (Rosenberg et al. Elife 2021). We now point the interested reader to all three references (p. 16).

Unfortunately, the reviewer's very intriguing question whether mTORC1- or its inhibition - also regulates the differentiation of human HF melanocyte stem cells, cannot currently not be satisfactorily answered in the human system, since no specific marker for HF melanocyte stem cells exists to date (in "fact, so-called "melanocyte stem cell markers" such as MITF, TRP1/2, or tyrosinase are by no means stem cell-specific). However, the interesting alternative hypothesis raised by this expert referee has encouraged us to now mention in the revised Discussion (p. 14): *"Once – as yet unavailable – truly selective markers for human HF melanocyte stem cells become available, it will deserve systematic scrutiny if mTORC1 inhibition by rapamycin also promotes the intrafollicular differentiation of melanocyte stem cells located in the bulge and/or of amelanotic melanoblasts located in the periphery of the proximal hair bulb epithelium (Tobin et al. 2011, O'Sullivan et al. 2021)."*

Given that their ex vivo model may not include the HF stem cell compartment, the authors could try to the measure tyrosinase activity of the more undifferentiated melanocytes found in the upper ORS layers after mTORC1 inhibition?

Tyrosinase activity is exclusively found in mature melanocytes where melanin production is active and even though ectopic pigmentation can be found in particular cases (e.g. stress) in ORS, tyrosinase activity is found in the HF pigmentary unit only (Tobin 2011, O'Sullivan et al. 2021). However, we have now specifically searched for evidence of tyrosinase activity outside of the HF pigmentary unit in HFs treated rapamycin: *No tyrosinase activity could be observed outside of the hair follicle pigmentary unit*. This has now been clarified in the revised Results section (p. 8)

Referee #2:

The body of work presented is extensive, and the data support the major conclusions of the paper. That being said, some details of the studies are lacking throughout the manuscript and, consequently, it is difficult to assess whether several conclusions drawn from some experiments are validated by the data shown, as detailed below.

1. Although the differences in p-S6 fluorescence intensity are obvious in Fig. 1, there is no description included concerning how images were acquired and fluorescence signals were quantified to ensure accuracy of measurements and conclusions. This aspect of the work needs to be addressed. Similar considerations are relevant for other experiments involving quantification of fluorescence signals.

In the original version of the manuscript, we had already indicated in the vehicle/control image for each marker the standardized reference area in which quantitative immunohistomophometry was performed. We have also clarified in the Material & Methods section how images were acquired and fluorescent signals were quantified (including corresponding references that refer to our standardized, well-published protocols): *"Images were acquired using the BZ-X800 All-in-one Fluorescence Microscope (Keyence Corporation, Osaka, Japan) maintaining a constant set exposure time throughout imaging. Images were taken at 200x magnifications. Image analysis and measurement of staining intensity were evaluated in well-defined reference areas (see vehicle pictures (dotted area)) using NIH ImageJ software (NIH, Bethesda, Maryland, USA), as previously described (Hardman et al, 2015; Tiede et al, 2021; Chéret et al, 2020; Gáspár et al, 2011). In brief, for measuring the staining intensity of our different markers, we first draw the reference area (see the dotted area on each figure) and measure the mean intensity. For analyzing the number of single and double positive cells, we have used the cell counter functions of Image J and individually pointed the positive (single and/or double)."* (p. 24)

2. Figure 2D appears to be incomplete, as micrographs of Catagen HF treated with rapamycin and Anagen HF treated with vehicle have been omitted. The description of the experimental methods appears to indicate that all HF used in these studies were in Anagen VI.

We had previously omitted micrographs of catagen HFs treated with rapamycin and anagen HF treated with vehicle for space constraints, since we considered these dispensable. However, as requested, we have now added the missing anagen and catagen HF images in the revised Fig 2D.

3. Although it is well known that melanocytes develop dendrites, the micrographs included do not allow evaluation of the abundance of dendrites in said melanocytes. Please include clear micrographs that demonstrate melanocyte dendricity in the tissue sections.

We thank the reviewer for this helpful suggestion. We have now replaced the corresponding images to demonstrate melanocyte dendricity more clearly in the tissue sections (see new Fig 2L).

4. The conclusions regarding the effect of rapamycin on melanosome transfer to HF keratinocytes (p.8) are not supported by the data shown, as the experiments presented are not designed to measure melanosome transfer from melanocytes to keratinocytes.

Her, we respectfully disagree with the referee. While transmission electron microscopy would be the optimal method of choice for visualizing melanosome transfer, gp100 IF (=demarcates premelanomes) is a well-recognized and sensitive tracker of melanosome transfer between melanocytes and keratinocytes (Singh et al 2008). We have now underscored this in the revised M+M (p. 23) and Results sections (p. 8), but acknowledge in the revised Discussion that

"*independent confirmation of this melanosome transfer effect by transmission electron microscopy would be desirable*" (p. 14).

5. The mechanistic experiments that included TSC silencing are very important. It is somewhat unusual that a relatively minor approximately 20% decrease in TSC2 abundance (as per immunofluorescence quantification shown in Fig. 3B, C would be associated with a major downstream effect. This should be further discussed.

Yes, this is indeed unusual and has intrigued us for a long time. In fact, in our extensive, published experience with gene silencing in organ-cultured scalp HFs (a method that we have pioneered in human hair research), a knockdown efficiency of 20-25%, and even of as little as 15%, can suffice to generate significant downstream functional effects in this highly sensitive *ex vivo* assay (Samuelov et al. JID 2012, Al-Nuaimi et al. JID 2014, Hardman et al. JID 2015, Cheret et al Nat Comm 2018, Mardaryev et al JID 2021). We have now clarified this in the revised Discussion, along with citing these references; here we now also emphasize that "*the major functional downstream effect observed despite the relatively modest knock-down efficiency highlights how tightly mTORC1 activity is regulated by TSC2 in human scalp HFs*" (p. 13).

6. The description of drug treatments is not clear and needs to be revised (P. 18). Specifically, the drugs were administered several times. However, it is not clear if a stock solution of 20 ng/ml rapamycin was used, or if the final concentration in the culture medium was 20 ng/ml. Similar considerations apply to ASIP and DMSO.

Thanks for urging us to provide greater clarity on this. We have now clarified this in the revised Materials and Methods section as follows: "*Rapamycin (Final concentration in each rapamycin-treated well: 20 ng/mL, Santa Cruz, sc-3504), Agouti Signaling Protein (ASIP)/Agouti protein (Final concentration in each agouti-treated well: 2 ug/ml, R&D system, 9094-AG) or vehicle (Final concentration in each vehicle-treated well: 0.1% DMSO) (Sigma-Aldrich) was administrated on day 1, 3 and 5 during medium change.*" (p. 19)

Further, it is not clear whether there were changes in the culture medium, so that rapamycin was always present at the same concentration, or whether there were simply additions to the existing culture medium, in which case the drug and vehicle concentrations varied from day 1 to day 7. The micrographs appear to indicate that the HF were cultured in the presence of 20 ng/ml rapamycin, but the description in the "Methods" section is not very clear.

The medium was completely replaced every other day (at day 1, 3 and 5). We have now clarified this in the revised Material and Methods section (see also comment above), where we have also pointed out that 20 ng/ml rapamycin corresponds to the final concentration: "*Rapamycin (Final concentration in each rapamycin-treated well: 20 ng/mL, Santa Cruz, sc-3504)…*" (p. 19)

7. Similarly, for siRNA experiments it is not clear whether a 1 uM stock solution (non-specified volume) was used, or the follicles were culture for 48 h in the presence of 1 uM siRNA.

Hair follicles were each cultured in the presence of 1 uM siRNA or non-targeting oligos for 48 hrs. We have now clarified this in the revised Material and methods: "*Final concentration of 1 µM*

of siTSC2 or siRNA control (NTO) was added 24 h after microdissection for 48 h in each of the corresponding wells." (p.20)

Minor points:

1. P4: "...TSC loss-of-function in mouse tissues and cultured fibroblasts show upregulated..." should read "...TSC loss-of-function in mouse tissues and cultured fibroblasts shows upregulated..."

Thank you for spotting this typo, which has been corrected as suggested (p. 4).

2. The micrographs in Fig. 2H are not of very good quality. Please replace.

Agreed. We have now replaced the corresponding images as requested (see new Fig 2H).

REFERENCES:

Böhm M, Eickelmann M, Li Z, Schneider SW, Oji V, Diederichs S, Barsh GS, Vogt A, Stieler K, Blume-Peytavi U, Luger TA. Detection of functionally active melanocortin receptors and evidence for an immunoregulatory activity of alpha-melanocyte-stimulating hormone in human dermal papilla cells. Endocrinology. 2005 Nov;146(11):4635-46.

Chéret J, Bertolini M, Ponce L, Lehmann J, Tsai T, Alam M, Hatt H, Paus R. Olfactory receptor OR2AT4 regulates human hair growth. Nat Commun. 2018 Sep 18;9(1):3624.

Chéret J, Gherardini J, Bertolini M, Paus R. Fluoxetine promotes human hair follicle pigmentation ex vivo: serotonin reuptake inhibition as a new antigreying strategy? Br J Dermatol. 2020 Jun;182(6):1492-1494.

Gáspár E, Nguyen-Thi KT, Hardenbicker C, Tiede S, Plate C, Bodó E, Knuever J, Funk W, Bíró T, Paus R. Thyrotropin-releasing hormone selectively stimulates human hair follicle pigmentation. J Invest Dermatol. 2011 Dec;131(12):2368-77.

Hardman JA, Tobin DJ, Haslam IS, Farjo N, Farjo B, Al-Nuaimi Y, Grimaldi B, Paus R. The peripheral clock regulates human pigmentation. J Invest Dermatol. 2015 Apr;135(4):1053-1064.

Ito N, Ito T, Kromminga A, Bettermann A, Takigawa M, Kees F, Straub RH, Paus R. Human hair follicles display a functional equivalent of the hypothalamic-pituitary-adrenal axis and synthesize cortisol. FASEB J. 2005 Aug;19(10):1332-4.

Kauser S, Thody AJ, Schallreuter KU, Gummer CL, Tobin DJ. A fully functional proopiomelanocortin/melanocortin-1 receptor system regulates the differentiation of human scalp hair follicle melanocytes. Endocrinology. 2005 Feb;146(2):532-43.

Mardaryev A, van Lessen M, Alam M, Jimenez Acosta F, Bíró T, Paus R. Hair Follicle Chemosensation: TRPM5 Signaling Is Required for Anagen Maintenance. J Invest Dermatol. 2021 Sep;141(9):2300-2303.

Singh SK, Nizard C, Kurfurst R, Bonte F, Schnebert S, Tobin DJ. The silver locus product (Silv/gp100/Pmel17) as a new tool for the analysis of melanosome transfer in human melanocyte-keratinocyte co-culture. Exp Dermatol. 2008 May;17(5):418-26.

Ständer S, Böhm M, Brzoska T, Zimmer KP, Luger T, Metze D. Expression of melanocortin-1 receptor in normal, malformed and neoplastic skin glands and hair follicles. Exp Dermatol. 2002 Feb;11(1):42-51.

Tiede S, Hundt JE, Paus R. UDP-GlcNAc-1-Phosphotransferase Is a Clinically Important Regulator of Human and Mouse Hair Pigmentation. J Invest Dermatol. 2021 Dec;141(12):2957-2965.e5.

Tobin DJ. The cell biology of human hair follicle pigmentation. Pigment Cell Melanoma Res. 2011 Feb;24(1):75-88.

Dear Dr. Paus,

Thank you for the submission of your revised manuscript. I have now evaluated your response to the minor concerns raised by the referees and I have also gone through your manuscript to see what we still require from the editorial side before your manuscript can be officially accepted.

- Your manuscript will be published in our Reports section. To make this possible, please combine the Results and Discussion sections.

- Please add callouts to Fig 1C&D, EV3A-L and EV4A-E.

- Please move the Data availability section to the end of the Materials & Methods section.

- Please update the 'Conflict of interest' paragraph to our new 'Disclosure and competing interests statement'. For more information see https://www.embopress.org/page/journal/14693178/authorguide#conflictsofinterest

- Regarding the Author Contributions, we now use CRediT to specify the contributions of each author in the journal submission system. CRediT replaces the author contribution section, which therefore needs to be removed from the manuscript text. You can use the free text box in our system if you wish to provide more detailed descriptions. See also guide to authors https://www.embopress.org/page/journal/14693178/authorguide#authorshipguidelines.

- Please move Table 1 to after the figure legends.

- Please remove the section "The Paper Explained".

- Please remove "the section Funding sources" or merge it with the information in the Acknowledgment section.

- During our routine image analysis, which we perform on all revised manuscripts before publication, we noticed that the same images are shown in Figure 2J and Figure EV1D. Since these are representative images for the staining you did, please choose a different set of images for Fig EV1D.

- At EMBO Press we now ask authors to provide source data for the main figures. On January 25th, 2023 our source data coordinator Hannah Sonntag contacted you regarding the deposition of source data, i.e., the minimally processed data that was used to generate the figures. She attached a checklist detailing which data were required. I hope this e-mail was not filtered by your spam filter. Please submit the source data alongside your revised manuscript. If you need any further information, please let me know.

Additional information on source data and instruction on how to label the files are available <https://www.embopress.org/page/journal/14693178/authorguide#sourcedata>.

- I attach to this email a related manuscript file with comments by our data editors. Please address all comments and upload a revised file with tracked changes with your final manuscript submission.

- Finally, EMBO reports papers are accompanied online by A) a short (1-2 sentences) summary of the findings and their significance, B) 2-3 bullet points highlighting key results and C) a synopsis image that is 550x300-600 pixels large (width x height) in PNG for JPG format. You can either show a model or key data in the synopsis image. Please note that the size is rather small and that text needs to be readable at the final size. Please send us this information along with the revised manuscript.

We look forward to seeing a final version of your manuscript as soon as possible.

Kind regards,

Martina Rembold, PhD
Senior Editor
EMBO reports

The authors have addressed all minor editorial requests.

2nd Revision - Editorial Decision 24th Apr 2023

Jérémy Chéret
University of Miami Miller School of Medicine
Dr. Phillip Frost Dept. of Dermatology & Cutaneous Surgery
United States

Dear Dr. Chéret,

I am very pleased to accept your manuscript for publication in the next available issue of EMBO reports. Thank you for your contribution to our journal.

At the end of this email I include important information about how to proceed. Please ensure that you take the time to read the information and complete and return the necessary forms to allow us to publish your manuscript as quickly as possible.

As part of the EMBO publication's Transparent Editorial Process, EMBO reports publishes online a Review Process File to accompany accepted manuscripts. As you are aware, this File will be published in conjunction with your paper and will include the referee reports, your point-by-point response and all pertinent correspondence relating to the manuscript.

If you do NOT want this File to be published, please inform the editorial office within 2 days, if you have not done so already, otherwise the File will be published by default [contact: emboreports@embo.org]. If you do opt out, the Review Process File link will point to the following statement: "No Review Process File is available with this article, as the authors have chosen not to make the review process public in this case."

Should you be planning a Press Release on your article, please get in contact with emboreports@wiley.com as early as possible, in order to coordinate publication and release dates.

Thank you again for your contribution to EMBO reports and congratulations on a successful publication. Please consider us again in the future for your most exciting work.

Yours sincerely,

Martina Rembold, PhD
Senior Editor
EMBO reports

****

THINGS TO DO NOW:

Please note that you will be contacted by Wiley Author Services to complete licensing and payment information. The required 'Page Charges Authorization Form' is available here: https://www.embopress.org/pb-assets/embo-site/er_apc.pdf - please download and complete the form and return to embopressproduction@wiley.com

EMBO Press participates in many Publish and Read agreements that allow authors to publish Open Access with reduced/no publication charges. Check your eligibility: https://authorservices.wiley.com/author-resources/Journal-Authors/open-access/affiliation-policies-payments/index.html

You will receive proofs by e-mail approximately 2-3 weeks after all relevant files have been sent to our Production Office; you should return your corrections within 2 days of receiving the proofs.

Please inform us if there is likely to be any difficulty in reaching you at the above address at that time. Failure to meet our deadlines may result in a delay of publication, or publication without your corrections.

All further communications concerning your paper should quote reference number EMBOR-2022-56574V3 and be addressed to emboreports@wiley.com.

Should you be planning a Press Release on your article, please get in contact with emboreports@wiley.com as early as possible, in order to coordinate publication and release dates.

EMBO Press Author Checklist

Corresponding Author Name: Ralf Paus	
Journal Submitted to: EMBO Reports	
Manuscript Number: EMBOR-2022-56574-T	

USEFUL LINKS FOR COMPLETING THIS FORM
The EMBO Journal - Author Guidelines
EMBO Reports - Author Guidelines
Molecular Systems Biology - Author Guidelines
EMBO Molecular Medicine - Author Guidelines

Reporting Checklist for Life Science Articles (updated January 2022)
This checklist is adapted from Materials Design Analysis Reporting (MDAR) Checklist for Authors. MDAR establishes a minimum set of requirements in transparent reporting in the life sciences (see Statement of Task: 10.31222/osf.io/9sm4x). Please follow the journal's guidelines in preparing your manuscript.
Please note that a copy of this checklist will be published alongside your article.

Abridged guidelines for figures
1. Data
The data shown in figures should satisfy the following conditions:
- the data were obtained and processed according to the field's best practice and are presented to reflect the results of the experiments in an accurate and unbiased manner.
- ideally, figure panels should include only measurements that are directly comparable to each other and obtained with the same assay.
- plots include clearly labeled error bars for independent experiments and sample sizes. Unless justified, error bars should not be shown for technical replicates.
- if n<5, the individual data points from each experiment should be plotted. Any statistical test employed should be justified.
- Source Data should be included to report the data underlying figures according to the guidelines set out in the authorship guidelines on Data Presentation.

2. Captions
Each figure caption should contain the following information, for each panel where they are relevant:
- a specification of the experimental system investigated (eg cell line, species name).
- the assay(s) and method(s) used to carry out the reported observations and measurements.
- an explicit mention of the biological and chemical entity(ies) that are being measured.
- an explicit mention of the biological and chemical entity(ies) that are altered/varied/perturbed in a controlled manner.
- the exact sample size (n) for each experimental group/condition, given as a number, not a range;
- a description of the sample collection allowing the reader to understand whether the samples represent technical or biological replicates (including how many animals, litters, cultures, etc.).
- a statement of how many times the experiment shown was independently replicated in the laboratory.
- definitions of statistical methods and measures:
  - common tests, such as t-test (please specify whether paired vs. unpaired), simple χ2 tests, Wilcoxon and Mann-Whitney tests, can be unambiguously identified by name only, but more complex techniques should be described in the methods section;
  - are tests one-sided or two-sided?
  - are there adjustments for multiple comparisons?
  - exact statistical test results, e.g., P values = x but not P values < x;
  - definition of 'center values' as median or average;
  - definition of error bars as s.d. or s.e.m.

Please complete ALL of the questions below.
Select "Not Applicable" only when the requested information is not relevant for your study.

Materials

Newly Created Materials	Information included in the manuscript?	In which section is the information available? (Reagents and Tools Table, Materials and Methods, Figures, Data Availability Section)
New materials and reagents need to be available; do any restrictions apply?	Not Applicable	

Antibodies	Information included in the manuscript?	In which section is the information available? (Reagents and Tools Table, Materials and Methods, Figures, Data Availability Section)
For **antibodies** provide the following information:<br>- Commercial antibodies: RRID (if possible) or supplier name, catalogue number and or/clone number<br>- Non-commercial: RRID or citation	Yes	Materials and Methods

DNA and RNA sequences	Information included in the manuscript?	In which section is the information available? (Reagents and Tools Table, Materials and Methods, Figures, Data Availability Section)
**Short novel DNA or RNA including primers, probes**: provide the sequences.	Yes	Primers are listed in the Materials and Methods

Cell materials	Information included in the manuscript?	In which section is the information available? (Reagents and Tools Table, Materials and Methods, Figures, Data Availability Section)
**Cell lines:** Provide species information, strain. Provide accession number in repository **OR** supplier name, catalog number, clone number, and/**OR** RRID.	Not Applicable	
**Primary cultures:** Provide species, strain, sex of origin, genetic modification status.	Not Applicable	
Report if the cell lines were recently **authenticated** (e.g., by STR profiling) and tested for mycoplasma contamination.	Not Applicable	

Experimental animals	Information included in the manuscript?	In which section is the information available? (Reagents and Tools Table, Materials and Methods, Figures, Data Availability Section)
**Laboratory animals or Model organisms:** Provide species, strain, sex, age, genetic modification status. Provide accession number in repository **OR** supplier name, catalog number, clone number, **OR** RRID.	Not Applicable	
**Animal observed in or captured from the field:** Provide species, sex, and age where possible.	Not Applicable	
Please detail **housing and husbandry conditions**.	Not Applicable	

Plants and microbes	Information included in the manuscript?	In which section is the information available? (Reagents and Tools Table, Materials and Methods, Figures, Data Availability Section)
**Plants:** provide species and strain, ecotype and cultivar where relevant, unique accession number if available, and source (including location for collected wild specimens).	Not Applicable	
**Microbes:** provide species and strain, unique accession number if available, and source.	Not Applicable	

Human research participants	Information included in the manuscript?	In which section is the information available? (Reagents and Tools Table, Materials and Methods, Figures, Data Availability Section)
If collected and within the bounds of privacy constraints report on age, sex and gender or ethnicity for all study participants.	Not Applicable	

Core facilities	Information included in the manuscript?	In which section is the information available? (Reagents and Tools Table, Materials and Methods, Figures, Data Availability Section)
If your work benefited from core facilities, was their service mentioned in the acknowledgments section?	Not Applicable	

Design

Study protocol	Information included in the manuscript?	In which section is the information available? (Reagents and Tools Table, Materials and Methods, Figures, Data Availability Section)
If study protocol has been **pre-registered, provide DOI in the manuscript**. For clinical trials, provide the trial registration number **OR** cite DOI.	Not Applicable	
Report the **clinical trial registration number** (at ClinicalTrials.gov or equivalent), where applicable.	Not Applicable	

Laboratory protocol	Information included in the manuscript?	In which section is the information available? (Reagents and Tools Table, Materials and Methods, Figures, Data Availability Section)
Provide DOI OR other citation details if **external detailed step-by-step protocols** are available.	Yes	Corresponding previously published experiemental design details have been cited in the Materials and Methods

Experimental study design and statistics	Information included in the manuscript?	In which section is the information available? (Reagents and Tools Table, Materials and Methods, Figures, Data Availability Section)
Include a statement about **sample size** estimate even if no statistical methods were used.	Yes	Materials and Methods
Were any steps taken to minimize the effects of subjective bias when allocating animals/samples to treatment (e.g. **randomization procedure**)? If yes, have they been described?	Yes	Materials and Methods
Include a statement about **blinding** even if no blinding was done.	Not Applicable	
Describe **inclusion/exclusion criteria** if samples or animals were excluded from the analysis. Were the criteria pre-established?<br><br>If sample or data points were omitted from analysis, report if this was due to attrition or intentional exclusion and provide justification.	Yes	Results
For every figure, are **statistical tests** justified as appropriate? Do the data meet the assumptions of the tests (e.g., normal distribution)? Describe any methods used to assess it. Is there an estimate of variation within each group of data? Is the variance similar between the groups that are being statistically compared?	Yes	Figures legends and Materials and Methods

Sample definition and in-laboratory replication	Information included in the manuscript?	In which section is the information available? (Reagents and Tools Table, Materials and Methods, Figures, Data Availability Section)
In the figure legends: state number of times the experiment was **replicated** in laboratory.	Yes	Figures legends
In the figure legends: define whether data describe **technical or biological replicates**.	Yes	Figures legends

Ethics

Ethics	Information included in the manuscript?	In which section is the information available? (Reagents and Tools Table, Materials and Methods, Figures, Data Availability Section)
Studies involving **human participants**: State details of **authority granting ethics approval** (IRB or equivalent committee(s), provide reference number for approval.	Not Applicable	Materials and Methods
Studies involving **human participants**: Include a statement confirming that **informed consent** was obtained from all subjects and that the experiments conformed to the principles set out in the WMA Declaration of Helsinki and the Department of Health and Human Services Belmont Report.	Not Applicable	
Studies involving **human participants:** For publication of **patient photos**, include a statement confirming that consent to publish was obtained.	Not Applicable	
Studies involving experimental **animals**: State details of **authority granting ethics approval** (IRB or equivalent committee(s), provide reference number for approval. Include a statement of compliance with ethical regulations.	Not Applicable	
Studies involving **specimen and field samples:** State if relevant **permits** obtained, provide details of authority approving study; if none were required, explain why.	Not Applicable	

Dual Use Research of Concern (DURC)	Information included in the manuscript?	In which section is the information available? (Reagents and Tools Table, Materials and Methods, Figures, Data Availability Section)
Could your study fall under dual use research restrictions? Please check biosecurity documents and list of **select agents and toxins** (CDC): https://www.selectagents.gov/sat/list.htm	Not Applicable	
If you used a select agent, is the security level of the lab appropriate and reported in the manuscript?	Not Applicable	
If a study is subject to dual use research of concern regulations, is the name of the **authority granting approval and reference number** for the regulatory approval provided in the manuscript?	Not Applicable	

Reporting

The MDAR framework recommends adoption of discipline-specific guidelines, established and endorsed through community initiatives. Journals have their own policy about requiring specific guidelines and recommendations to complement MDAR.

Adherence to community standards	Information included in the manuscript?	In which section is the information available? (Reagents and Tools Table, Materials and Methods, Figures, Data Availability Section)
State if relevant guidelines or checklists (e.g., **ICMJE, MIBBI, ARRIVE, PRISMA**) have been followed or provided.	Not Applicable	
For **tumor marker prognostic studies,** we recommend that you follow the **REMARK** reporting guidelines (see link list at top right). See author guidelines, under 'Reporting Guidelines'. Please confirm you have followed these guidelines.	Not Applicable	
For **phase II and III randomized controlled trials**, please refer to the **CONSORT** flow diagram (see link list at top right) and submit the CONSORT checklist (see link list at top right) with your submission. See author guidelines, under 'Reporting Guidelines'. Please confirm you have submitted this list.	Not Applicable	

Data Availability

Data availability	Information included in the manuscript?	In which section is the information available? (Reagents and Tools Table, Materials and Methods, Figures, Data Availability Section)
Have **primary datasets** been deposited according to the journal's guidelines (see 'Data Deposition' section) and the respective accession numbers provided in the Data Availability Section?	Yes	Data Availability Section
Were **human clinical and genomic datasets** deposited in a public access-controlled repository in accordance to ethical obligations to the patients and to the applicable consent agreement?	Not Applicable	
Are **computational models** that are central and integral to a study available without restrictions in a machine-readable form? Were the relevant accession numbers or links provided?	Not Applicable	
If publicly available data were reused, provide the respective **data citations in the reference list**.	Not Applicable	
